# Protecting cells at the genetic level and simulating unauthorized access via a biohackathon

**DOI:** 10.1126/sciadv.aeb8556

**Published:** 2026-04-01

**Authors:** Dowan Kim, Ishita Kumar, Mohamed I. Hassan, Luisa F. Barraza-Vergara, Christopher A. Voigt, Corey J. Wilson

**Affiliations:** ^1^ Georgia Institute of Technology, School of Chemical & Biomolecular Engineering, 311 Ferst Drive, Atlanta, GA 30332, USA.; ^2^ Massachusetts Institute of Technology, Department of Biological Engineering, Synthetic Biology Center, Cambridge, MA 02139, USA.

## Abstract

The protection of high-value cell lines (assets) relies on physical security by limiting access to samples. We present a cybersecurity-inspired platform that protects biological assets at the genetic level. This technology uses a permutation lock design where an asset can only be decrypted using an authentication code *r* from a search space composed of *n* objects on a defined keypad. Here, the genetic asset is designed as a scrambled DNA sequence, and the code is a temporal pattern of small molecules that regulate sets of recombinases that can unscramble a DNA sequence into the desired final sequence. In this work, a “blue team” designed and built an encrypted (scrambled) DNA sequence, and a “red team” sought to break the code through an ethical hacking exercise. Two iterations of testing revealed a 0.2% (2 in 990) chance of gaining access to the asset by random search, which is on par with the theoretical goal of 0.1% (1 in 990).

## Teaser

Genetic-level biological security technology is deployed, inspired by cybersecurity red team versus blue team strategies.

## INTRODUCTION

A record number of unauthorized shipments and use of biological materials have been flagged by the Center for Disease Control and Prevention, Department of Homeland Security, and other authorities in recent years (*1*–*4*). Congruent with these events, intelligence communities globally have identified myriad attempts to smuggle high-value cells and other sensitive biological materials in efforts of industrial theft or espionage (*5*–*8*). For the most part, high-value cells and other sensitive biological materials are solely protected via physical security measures, e.g., access control, perimeter security, surveillance, and the like. The key weakness of physical security measures is that, once circumvented, there are typically no measures in place to protect valuable cells from theft, abuse, or unauthorized use (*9*–*11*). We posit that the gaps in the physical security measures used to protect valuable cells can be corrected or mitigated by way of intrinsic security measures that protect biological assets directly at the genetic level, even after physical security measures have been circumvented.

Many contemporary valuable cells and biological materials [e.g., plants, organoids (*12*), and tissues] fundamentally come in the form of engineered cells that contain native and heterologous biosynthesis pathways (*13*–*16*), recombinant proteins (*17*–*21*), and other synthetic or natural DNA sequences (*22*–*27*). At the most fundamental level, the valuable genetic sequence(s) contained in high-value cell lines can be generally represented as one or more engineered DNA sequence(s) composed from a string of genetic parts that can express a gene of interest (GoI) or produce a noncoding RNA sequence. The most recent global valuation of said high-value genetic assets is estimated at more than $1.5 trillion USD and is projected to reach $8.0 trillion USD by 2035 (*28*–*31*). Scientists and engineers have initiated the transition to intrinsic biosecurity measures, notably in the context of biocontainment strategies (*32*–*41*). Biocontainment approaches have the potential to mitigate the release of high-value cells that contain a genetic asset; however, given the correct growth conditions, the valuable (or sensitive) DNA sequence(s) remains unprotected. On balance, identifying the permissive environment for a biocontainment system may not necessarily be trivial and could require searching a near infinite number of growth conditions. Conjecturing from the position of addressing the gaps in physical security measures and how to better complement biocontainment approaches, we posit that the next generation of biological security measures will enable direct protection of a genetic asset via the application of some form of DNA scrambling (encryption) and unscrambling (decryption) technology (*42*–*49*) guided by cybersecurity design and test strategies (*50*–*53*).

**Fig. 1.**
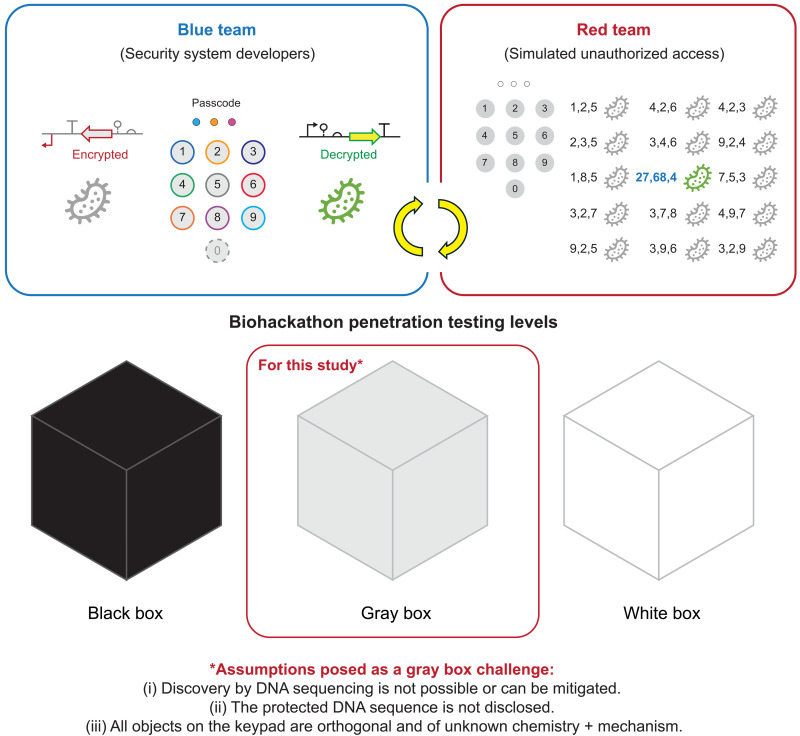
Biological security scenario “biohackathon” for designing, building, testing, and learning. Development of security systems to protect cells at the genetic level via a scenario-based design and test strategy accomplished by a blue team versus red team exercise. The blue team is the technical group responsible for developing the security system. Here, the security system is based on a permutation lock design where an asset can only be decrypted using an authentication code *r* from a search space composed of *n* objects on a defined keypad. The red team is the technical group responsible for simulating unauthorized access of the security system (i.e., ethical hacking*) usually conducted as a gray box challenge to facilitate testing and learning. *Ethical hacking is a process used to identify security vulnerabilities with legal permission from the system developers. Biohackathon penetration testing levels: Black box: The red team has no prior knowledge of the security system, and there are no restrictions on how the system can be hacked. This is the closest exercise to a real-world scenario; however, this is likely the most difficult system to objectively test and learn from. Gray box: The red team is provided with partial information regarding a specific security system to facilitate testing and learning. White box: The red team has full operational details and can extensively learn and probe the entire security system without restrictions. In this study, we make three assumptions posed as a gray box challenge: (i) Discovery by DNA sequencing is not possible or can be mitigated. (ii) The protected DNA sequence is not disclosed. (iii) All objects on the keypad are orthogonal and of unknown chemistry + mechanism.

**Fig. 2.**
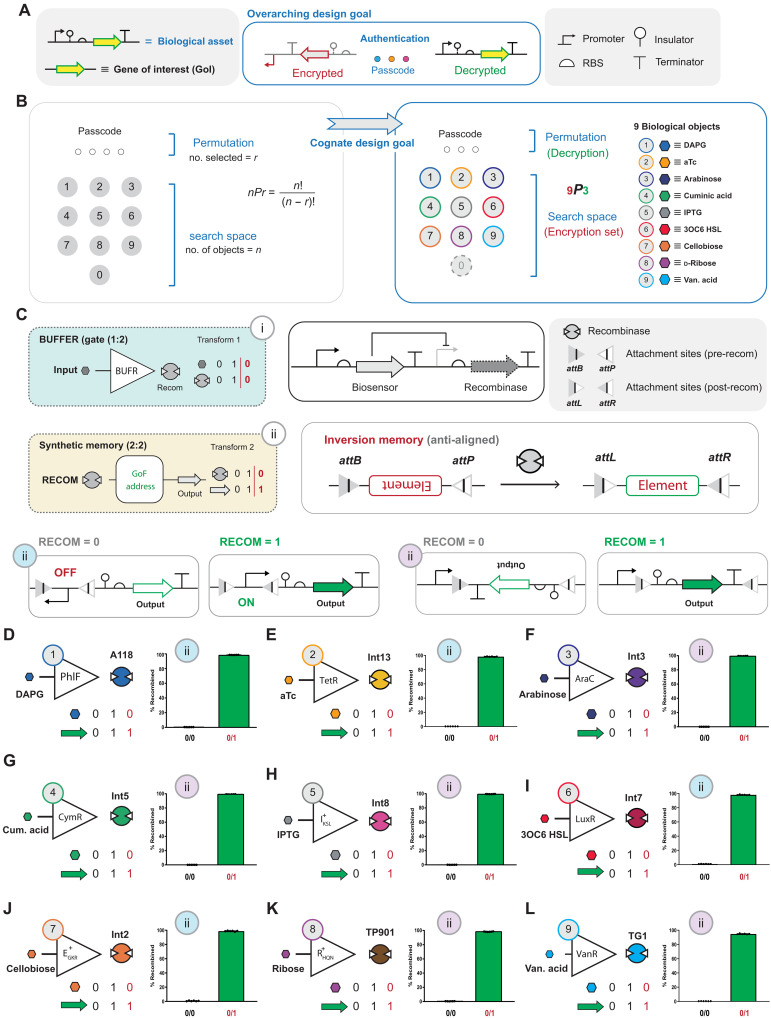
Illustration of biological (genetic) asset encryption to decryption and *nPr* object engineering. (**A**) The biological asset represented as a DNA sequence composed to express a GoI, and consists of a promoter, genetic insulator, ribosome binding site (RBS), GoI, and terminator. The goal is to encrypt the DNA sequence, making it decryptable only with the correct authentication code. (**B**) The security technology is predicated on an *nPr* permutation lock design, where *n* represents the number of objects, *r* sets the length of the chemical passcode, and *nPr* represents the search space. Authentication via the correct chemical passcode results in the decryption of the asset. (**C**) The design of a one-digit object requires two operations: (i) a biosensor that regulates a recombinase (RECOM), abstractly represented as a BUFFER (BUFR) operation; and (ii) synthetic gain-of-function (GoF) genetic memory via the induced inversion of a set of anti-aligned recombinase attachment sites (*attB* and *attP*) cognate to the recombinase BUFFER. The outcome upon removal of the input signal is represented by red bars, zeros (0), and ones (1). GoF synthetic memory via the induced inversion of a promoter is designated as a blue #ii, whereas inversion of a *gfp* expression cassette minus the promoter is designated as a purple #ii. (**D** to **L**) Fluorescence-activated cell sorting (FACS) analysis of nine successfully engineered one-digit biological objects. All one-digit objects are orthogonal in terms of chemical inducers and recombinase biochemistry. GoF memory is permanent and inheritable such that the *gfp* output state persists after the input is removed (denoted by red-colored 0/1), and 0/0 represents the OFF state of the circuit. The percentage of population recombination upon transient induction (0/1) is given as a bar graph for each object. Source data are provided in table S1. Data represent the average of *n* = 6 biological replicates.

Here, we aim to develop genetic-level security systems via a scenario-based (blue team versus red team) design and test strategy (Fig. 1), commonly used in cybersecurity developments (*50*–*53*). The blue team is responsible for designing and constructing the security system for a defined genetic asset in the form of an encrypted (scrambled) DNA sequence (Fig. 2A), whereas the red team is responsible for conducting the ethical hacking exercise to decrypt (unscramble) the encrypted genetic asset. The design for the security technology is analogous to a passcode protected smartphone (Fig. 2B) and relies on two components: (i) entries in the form of a permutation string (i.e., an authentication code) with a defined length *r* and (ii) a keypad with a fixed set of objects *n* to create a nontrivial permutation search space *nPr*. For example, on a cellphone a six-digit authentication code from a keypad selection composed of 0, 1, 2, 3, 4, 5, 6, 7, 8, and 9 would result in 10*P*6 or 151,200 possible permutations, of which only one code would be valid. In other words, this would result in a 0.0007% chance of gaining access to the system by random search. This calculation is based on a permutation space without repetition of an entry used in the authentication code, e.g., object 3 can only be used once in the passcode.

In this study, the blue team developed an encrypted genetic asset contained within *Escherichia coli* cells that can only be decrypted via a permutation of chemical inputs (Fig. 2B). The structure of the initial “biological keypad” was composed from nine one-digit objects (*n* = 9) achieved by mapping nine orthogonal biosensors (*54*–*57*) to nine orthogonal recombinases (*47*, *48*, *58*–*60*). In turn, an expansion of the keypad search space was achieved via the development of additional objects that require the concurrent entry of two chemicals to regulate the production of additional recombinases, i.e., two-digit objects. The additional two-digit objects allowed the expansion of the search space within the initial keypad from *n* = 9 to *n* = 45 objects, or 45*Pr*.

Next, the blue team systematically constructed scrambled DNA sequences composed from fundamental elements via leveraging recombinase-based strategies (*43*, *47*, *48*). However, in this study, the resulting encrypted DNA sequences were engineered to be responsive to defined objects represented within the biological keypad search space. Using these building blocks, the blue team composed scrambled DNA sequences that could be decrypted via two (*r* = 2) or three (*r* = 3) small molecule entries. This resulted in a permutation lock search space with an upper limit greater than 85,000 temporal patterns, given a 45*P*3 security system.

Last, the blue team transferred an 11*P*3 biological security system (i.e., a search space on the order of 10^3^) to an independent red team for comprehensive testing via an ethical hacking exercise. The ethical hacking exercise was conducted as a gray box challenge, in which the red team had limited knowledge of the security system (Fig. 1 and fig. S1). In brief, the constraints assume that (i) the asset is a nondisclosed DNA sequence, (ii) discovery via DNA sequencing can be mitigated, and (iii) all objects on the keypad are orthogonal and of unknown chemistry as well as cognate mechanism. In this challenge, the objective is to conduct a code breaking exercise, i.e., a “biohackathon.” that is tractable but nontrivial, with the goal of identifying any technical flaws within the engineered security system or operating procedure. The red team narrowed the permutation space by 99% in ~30 days, resulting in an ~1.0% chance of gaining access to the asset by random search. The blue team used the data from said assessment to make improvements to the security protocol. A second iteration of testing by the red team applying the revised protocol resulted in an ~0.2% chance of gaining access to the asset by random search, on par with the intended ~0.1% that was projected from the permutation calculation.

## RESULTS

### Engineering a nine (*n* = 9)–object biological keypad via one-digit genetic memory

The blue teams overarching objective focused on designing a form of biological authentication in the context of an *nPr* permutation lock to facilitate access to an encrypted DNA sequence, which expresses a GoI once decrypted (Fig. 2A). The putative biological keypad is designed to have each object represented by a chemical input. The design goal required that the blue team account for history or permutation, i.e., the order in which the inputs occur. To accomplish this, each chemical input corresponded to a biosensor, mapped to a recombinase, abstractly represented as a BUFFER gate (Fig. 2C). In turn, each recombinase could act on a set of attachment sites. The alignment of a set of attachment sites determines the operation, i.e., DNA inversion or deletion, which can be engineered to facilitate gain-of-function (GoF) or loss-of-function inheritable memory (fig. S2). For each object on the putative keypad the blue team’s objective focused on engineering a binary memory operation, where 0 input corresponds to 0 output and 1 input results in inversion of a given set of attachment sites such that the removal of the input retains an output state of 1 (Fig. 2C). Herein, we will refer to a BUFFER mapped to a single memory operation as a one-digit object entry.

To structure each one-digit object of the keypad, the blue team identified nine orthogonal biosensors strategically selected from natural (*54*) and synthetic (*55*) sources (fig. S3). Next, the blue team identified nine orthogonal recombinases (*47*, *48*, *58*–*60*) and assigned each biosensor to a given recombinase to form nine orthogonal BUFFER operations. To complete the construction of the one-digit object operations, the blue team initially allocated five BUFFER operations to invert a promoter and four BUFFER operations to invert an expression cluster, both designed as GoF memory (Fig. 2, C to L, and fig. S4). Promoter and expression cluster operations are designated as short-frame memory (~300 base pairs) and long-frame memory (~1000 base pairs), respectively. Memory frame-length is strategically designed to facilitate DNA sequence encryption, as discussed in the next section. The blue team postulated that differences in recombination frame-length will have an impact on recombination efficiency. However, all one-digit objects could be engineered with prescribed function. Namely, all objects achieved (i) minimal recombination in the OFF state (less than 2% recombination) and (ii) maximum recombination in the ON state (greater than 98% recombination) upon transient induction in less than 24 hours. To accomplish this, the blue team constructed libraries for each memory operation composed of variable promoter strength correlated to biosensor expression, variation in ribosome binding site (RBS) strength with respect to recombinase expression, start codon degeneracy cognate to the recombinase, and variation in recombinase lifetime modulated via degradation tags (also see fig. S4). The justification for said performance objectives is predicated on the requirements needed to form robust multiple entry decryption of an asset in *E. coli* under batch growth conditions, as discussed in the next section. In other words, the expectation is that any combination of two or more sets of orthogonal recombinase attachment sites used to encrypt a defined DNA sequence should theoretically result in 0% of the population producing the asset without authentication.

The blue team measured the performance of each one-digit object operation via fluorescence-activated cell sorting (FACS) Fig. 2 (D to L). Congruent with the design goal, all nine one-digit biological objects resulted in near digital performance, measured by percentage of single-cell recombination events. After one-digit object optimization, the blue team measured the kinetics of registering short-frame and long-frame recombination (fig. S5). In general, short-frame recombination is faster than long-frame recombination, and, with few exceptions, all one-digit objects achieved ~99% recombination after ~12 hours based on the conditions tested (see Materials and Methods). In addition, the blue team tested the orthogonality of the objects represented in the initial keypad and confirmed that one-digit objects were devoid of cross-induction (fig. S6). To demonstrate that the engineering workflow is generalizable, the blue team successfully remapped two of the biosensors to different recombinases, forming alternate one-digit objects (fig. S7). In summary, the blue team successfully developed *n* = 9 objects setting the first boundary condition for the primary search space at 9*Pr*.

### *nP*2 asset encryption via one-digit objects

**Fig. 3.**
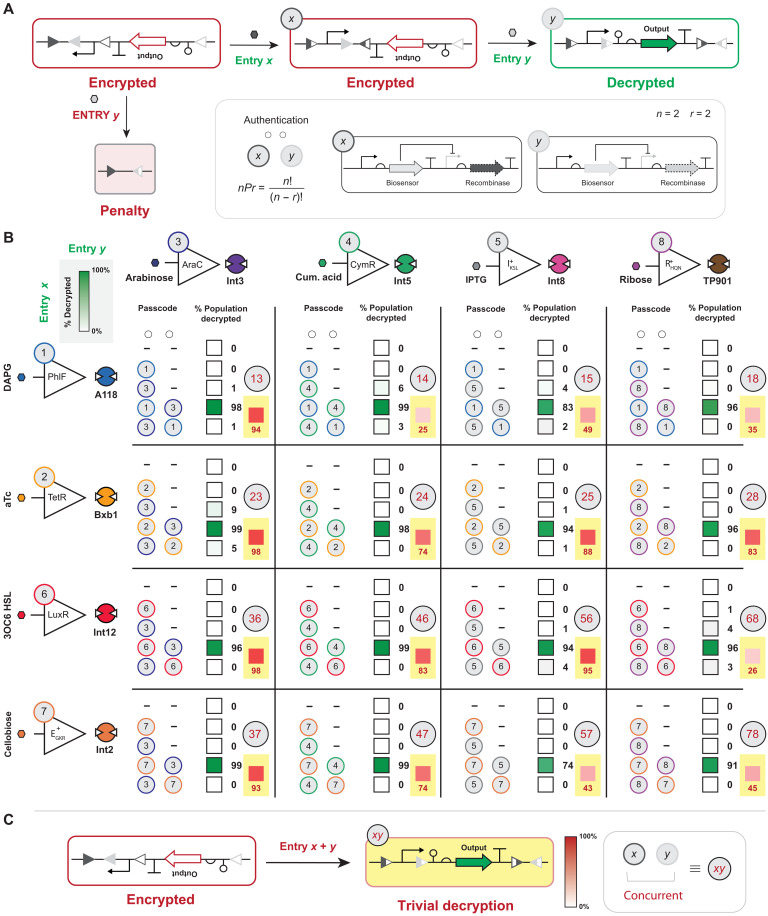
*nP*2 biological (genetic) permutation locks. (**A**) Schematic of the genetic structure and mechanism for an *nP*2 permutation lock is shown. The encrypted circuit is designed with two disparate *att* sites that are nested to form a sequential rearrangement of the asset upon exposure to cognate recombinases. The correct authentication code (i.e., input *x* followed by input *y*) results in the decryption of the genetic asset, whereas the incorrect input sequence results in the deletion of the genetic asset. (**B**) Sixteen *nP*2 permutation locks were tested via all possible entry permutations, i.e., no inducer, inducer *x* only, inducer *y* only, inducer (*x* followed by *y*), and inducer (*y* followed by *x*). The percentage of the decrypted population by each authentication code is represented on a green color scale, with the actual percentage noted on the right of the box. A dash (−) indicates no inducer was applied, otherwise each input was introduced sequentially (see Materials and Methods). Any observation over 5% from an incorrect authentication code is likely the result of spurious genetic decryption events. (**C**) The encrypted circuit can also be decrypted by introducing both inputs simultaneously. Concurrent induction of input *x* and *y* results in partial decryption, leading to circuit infiltration. The red box with a yellow background in (B) indicates trivial-decryption testing, where entries *x* and *y* were induced concurrently represented as a gray circle with the entries given in red. Source data are provided in table S1. Data represent the average of *n* = 6 biological replicates.

In this study, we defined the biological asset as a simple DNA sequence composed to express a GoI, i.e., a promoter, genetic insulator, RBS, green fluorescent protein (*gfp*), and terminator (Fig. 2A). The blue team proposed a general design for an *nP*2 lock to protect the genetic asset that can exist in two encrypted states or a single decrypted state (Fig. 3A). The definition for genetic encryption used in this study is congruent with that given by Purcell *et al.* (*43*). DNA sequence encryption was achieved via strategically arranging sets of recombinase attachment sites in the context of said genetic elements that compose the asset (also see fig. S8). The objective in this section is to demonstrate that the blue team could systematically construct encrypted functional DNA sequences that could be faithfully decrypted by different sets of two entry permutations. As generally designed, the encrypted functional DNA sequence used two sets of attachment sites (i.e., level 2 security) such that (i) the recombination of the set corresponding to entry *x* results in the inversion of a promoter; (ii) whereas the set corresponding to entry *y* facilitates the inversion of a cluster of elements, i.e., insulator, RBS, *gfp*, and terminator; and (iii) the second recombination event cognate to entry *y* can only occur after the first (entry *x*) recombination event. All two-entry permutation (*x*,*y*) locks designed in this section were composed from the set of extant one-digit objects developed in Fig. 2. In addition, the encrypted DNA sequences were designed such that an out-of-sequence permutation (i.e., *y*, *x*) results in the deletion of the asset (Fig. 3A). The blue team designed built and tested 16 iterations of *nP*2 permutation locks that can be decrypted using two one-digit object entries in a prescribed order (Fig. 3B). Consistent with the blue team’s design goals, all functional DNA sequences remained encrypted until the correct sequence of inputs was introduced. Notably, all level 2 encrypted DNA sequences retained an OFF-state population at or near 0% unless authenticated with the correct chemical passcode, on the basis of the conditions used for single-cell analysis (see Materials and Methods). Moreover, as the encryption level increases, the number of genetic states will also increase, e.g., level 2 encryption corresponds to four genetic states, level 3 encryption corresponds to seven genetic states and so forth. The blue team posited that, as the encryption level increases (i.e., greater than level 2), the OFF state of incomplete or incorrect chemical authentication of the population will theoretically be 0%, barring any issues with the design composability of functional DNA sequences from simpler parts.

### Expanding the biological keypad via engineered two-digit objects

**Fig. 4.**
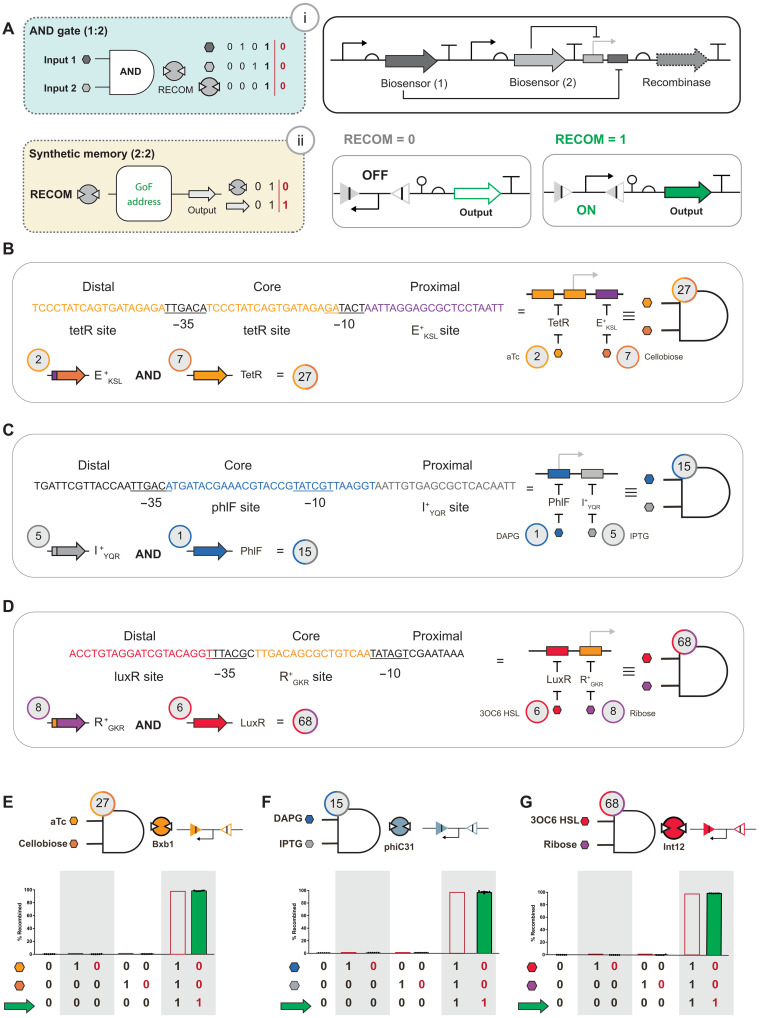
Engineering two-digit biological objects for keypad expansion. (**A**) Schematic illustration for the general design of a two-digit biological object pairing natural and synthetic biosensors. In summary, the mechanism requires the induction of both transcription factors to facilitate the expression of a given recombinase. The expression of said recombinase is mapped to a GoF memory operation. (**B** to **D**) Hybrid synthetic promoters engineered relative to consensus sets of hexamer elements at positions −35 and −10. Each hybrid synthetic promoter required the strategic placement of disparate biosensor DNA binding sites at the core, proximal, or distal positions. Said hybrid promoters were derived from the general natural biosensor promoters, with a synthetic DNA binding element inserted at varying positions. (B) Hybrid promoter responsive to TetR and E^+^_KSL_ (#27) is shown and requires aTc and cellobiose to activate expression of a GoI. (C) A hybrid promoter for PhlF and I^+^_YQR_ (#15) requires DAPG and IPTG to express the GoI. (D) Hybrid promoter responsive to LuxR and R^+^_GKR_ (#68) requires 3OC6 HSL and ribose to express the GoI. (**E** to **G**) Performance of the two-digit biological keys is demonstrated. (E) The TetR and E^+^_KSL_ (#27) hybrid promoter is mapped to recombinase Bxb1. (F) The PhlF and I^+^_YQR_ (#15) hybrid promoter is mapped to recombinase phiC31. (G) The LuxR and R^+^_GKR_ (#68) hybrid promoter is mapped to recombinase Int12. The red zeros (0) and red ones (1) represent the outcome upon removal of the input signal. The red bars for 1,1,1 represent the theoretical maximum after approximately a 12-hour incubation period. Source data are provided in table S1. Data represent the average of *n* = 6 biological replicates. Error bars correspond to the SEM of these measurements.

One strategy to increase the security of any permutation lock is to increase the number of objects (*n*) used to define the search space. In the context of this study, an obvious solution that would enable an increased number of objects on the biological keypad would be to map additional biosensors to other recombinases. However, this would most certainly increase the metabolic burden (*61*) on the *E. coli* cell. To engineer an expanded set of objects with less metabolic burden on the cell, the blue team posited that two-digit objects could be composed that repurpose biosensors from the validated one-digit object set illustrated in Fig. 2. Specifically, the blue team postulated that hybrid synthetic promoters could be engineered to direct two (or more) biosensors from different classes, i.e., one natural (e.g., PhlF, TetR, and LuxR) and one synthetic (e.g., I^+^_YQR_, E^+^_KSL_, and R^+^_GKR_) (fig. S3). The resulting hybrid synthetic promoters when paired with cognate biosensors would objectively form AND Boolean logical operations that can regulate the expression of any of the recombinases from the initial set (or additional recombinases beyond the extant set) to compose two-digit genetic memory (Fig. 4A).

The blue team based the design of each hybrid synthetic promoter on the consensus sets of hexamer elements at positions −35 and −10. Hybrid promoter designs are predicated on the ability to replace a portion of the region near or within the hexamer elements, i.e., core, proximal, or distal (*62*–*64*). The workflow for the construction of a hybrid synthetic promoter started with the general layout for the promoter for a given natural biosensor. Next, the blue team placed a DNA binding element cognate to a given synthetic biosensor at an unencumbered core, proximal, or distal position within the putative promoter (Fig. 4, B and C). To test said workflow, the blue team designed a hybrid synthetic promoter to direct the binding of (i) the natural biosensor TetR and (ii) the synthetic biosensor E^+^_KSL_ (Fig. 4B). The resulting TetR/E^+^_KSL_ hybrid promoter was tested as a simple AND gate regulating the expression of GFP (fig. S9). The AND gate performed as expected such that expression of GFP was only observed with both inputs present, i.e., aTc (object #2) and cellobiose (object #7), herein designated as object #27. Using the same workflow, the blue team successfully designed, built, and tested two additional hybrid synthetic promoters corresponding to (i) object #1 [AND] object #5, in addition to (ii) object #6 [AND] object #8 (Fig. 4, C and D, and fig. S9). The PhlF/I^+^_YQR_ (object #15) element is designed using a similar layout to the TetR/E^+^_KSL_ (object #27) hybrid promoter, i.e., with the synthetic DNA binding element located at the proximal position. However, the LuxR/R^+^_GKR_ (object #68) hybrid synthetic promoter is designed with the synthetic DNA binding element at the core position.

Once the AND gate portion for each system was validated, the blue team optimized the expression and lifetime of a recombinase designated to a set of attachment sites aligned for the inversion of a promoter setup to facilitate a two-digit object GoF memory operation with similar performance metrics to one-digit objects (Fig. 4, E to G, and fig. S4). To demonstrate the generalizability of the workflow, the blue team mapped object #15 to a previously assigned recombinase (i.e., A118) and achieved similar performance to other two-digit object and one-digit object memory operations (fig. S9). Given all one-digit and two-digit objects are mapped to orthogonal recombinases, said results allowed the blue team to expand the *nP*2 permutation space by nearly a factor of two, i.e., from 9*P*2 or 72 permutations to 12*P*2 or 132 permutations. Studies have demonstrated that AND gates can be generated via pairs of natural biosensors (*54*, *65*) and via pairs of synthetic biosensors (*55*, *66*, *67*). Thus, the inclusion of hybrid synthetic promoters fills an important gap in our technical ability and facilitates the combination of any two objects. In the context of the nine one-digit objects that the blue team has developed, this results in 36 putative two-digit objects, i.e., digit *XY* = digit *YX*, without restrictions. In principle, accounting for said combinations, this results in a 45*P*2 search space or 1980 permutations, noting that not all AND gates have been experimentally validated.

### Enhanced *nP*2 asset protection via two-digit objects

**Fig. 5.**
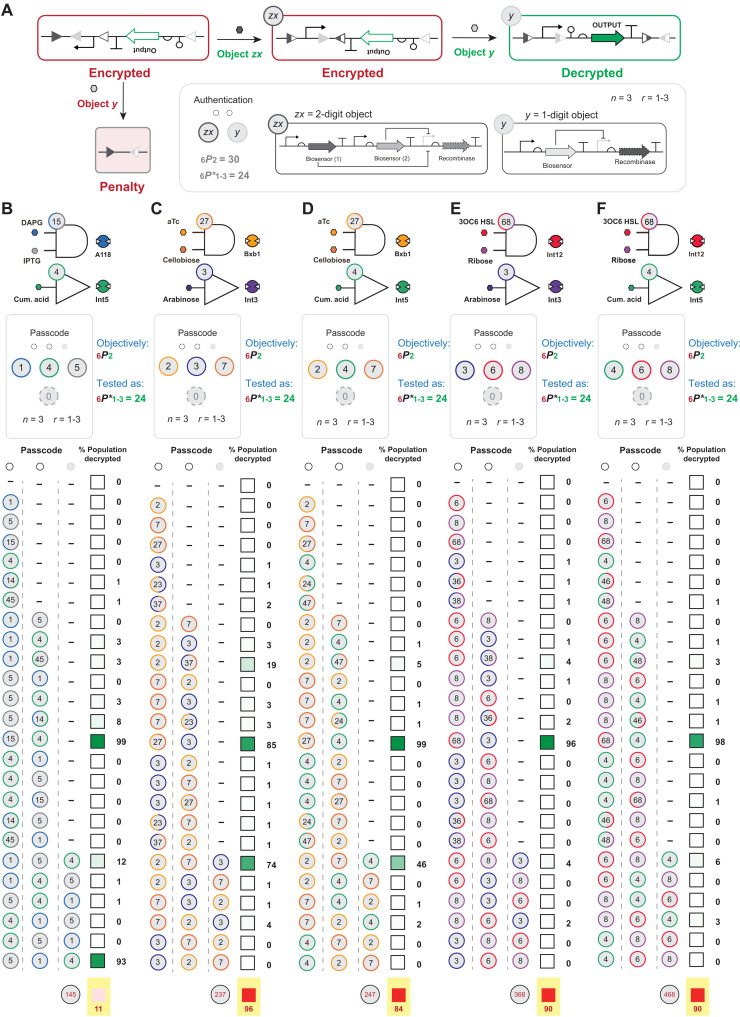
*nP*2 permutation locks with two-digit biological keys. (**A**) Schematic of an *nP*2 permutation lock that leverages one-digit, and two-digit objects is shown. Unlike previous *nP*2 permutation locks given in Fig. 2, this security system requires a two-digit object entry as part of the *r* = 2 permutation string. The encrypted circuit is designed with two disparate *att* sites that are nested to form a sequential rearrangement of the asset upon exposure to cognate recombinases. The correct authentication code (i.e., input *zx* followed by *y*) decrypts the DNA sequence, whereas an incorrect passcode (i.e., starting with *y*) results in the deletion of the genetic asset, objectively represented as 6*P*2. (**B** to **F**) Five examples of *nP*2 permutation locks using two-digit biological objects are engineered and tested as 6P*1-3 = 24: (B) two-digit #15 mapped to A118 and one-digit #4 mapped to Int5, (C) two-digit #27 mapped to Bxb1 and one-digit #3 mapped to Int3, (D) two-digit #27 mapped to Bxb1 and one-digit #4 mapped to Int5, (E) two-digit #68 mapped to Int12 and one-digit #3 mapped to Int3, and (F) two-digit #68 mapped to Int12 and one-digit #4 mapped to Int5. The percentage of the decrypted population by each authentication code is represented on a green color scale, with the actual percentage noted on the right of the box. Nomenclature (−) indicates no inducer was introduced; when applicable, each input was introduced sequentially, analogous to the schema used in Fig. 1B. [(B) to (F)] Bottom: Trivial-decryption test given as a red scaled box with a yellow background, i.e., all three objects were introduced concurrently for each security system with the decryption % given below each box. Source data are provided in table S1. Data represent the average of *n* = 6 biological replicates.

Next, the blue team designed an additional set of permutation locks for the representative level 2 encrypted DNA sequence given in Fig. 3A. However, in this iteration the blue team paired a one-digit object entry, with a two-digit object entry (Fig. 5A). Unlike conventional *nP*2 permutation locks that strictly use one-digit objects from a defined keypad, two-digit object entries facilitate an important and noncanonical expansion of the number of possible permutations within the same search space. For example, a keypad composed of *n* = 3 objects operated exclusively via one-digit object entries (i.e., 3*P*2) results in six permutations. In contrast, two-digit object entries allow the user to concurrently engage two objects as a distinct entry increasing the same keypad by an additional three objects, i.e., 6*P*2 resulting in 30 permutations. Even when the permutation selection is limited by the restriction that an object can only be used once exclusively in a permutation string this still results in 12 enumerated permutations for *r* = 2 (i.e., defined as 6P*2).

To test the practicality of this iteration of keypad entry on a level 2 encrypted DNA sequence constrained by a three object keypad, the blue team engineered five permutation locks that used a combination of one-digit and two-digit objects and exhaustively tested the permutation space for *r* = 1, 2, and 3. The blue team tested all 24 nonredundant permutations for each security system (Fig. 5, B to F). As with the level 2 encrypted DNA sequences that used one-digit object entries (illustrated in Fig. 3), in all cases, each permutation lock was reliably decrypted via the correct authentication code. Moreover, whenever the correct objects are engaged out of sequence, the system is not decrypted, validating the distinction between one-digit object and two-digit object entries. In two of the five examples, only the correct permutation string decrypted the DNA sequence (Fig. 5, E and F). In all other cases, no more than one incorrect permutation resulted in substantial (i.e., greater than 40%) decryption of the asset (Fig. 5, B to D). In each case where the asset is decrypted by an incorrect code involved an *r* = 3 entry sequence in which the first two entries were cognate to the two-digit object. Moreover, this is only observed for one of the permutations opposed to both, e.g., 2,7,4 resulted in ~46% decryption of the population, whereas 7,2,4 does not result in substantial decryption of the population, such that the correct authentication code is 27,4. This result implies that, in some cases, the ligand input history is retained because of a residual interaction between the small molecule and the biosensor due to insufficient dilution. In such cases, additional dilution between entries (or reduction) in input concentration resolved this issue (fig. S10). In general, this set of results demonstrated the scalability and fidelity of the blue team’s security designs using two-digit object entries.

### Advanced *nP*3 asset encryption

In addition to keypad expansion, increasing the length (*r*) of the authentication code is another strategy to increase the security of any permutation lock. For example, in the context of our biosecurity developments a 9*P*2 security system results in 72 possible permutations, whereas a 9*P*3 expands the search space to 504 unique variations. Moreover, including two-digit objects within the same fundamental keypad results in 1980 permutations with respect to 45*P*2, which increases to 85,140 permutations via a 45*P*3 application. Notably, excluding number overlap between one-digit and two-digit objects within a 45*P*3 search space results in 25,317 permutations, denoted as 45P*3. The restricted 45P*3 search space is 50 times larger than the primary 9*P*3 premutation space and more than 12 times larger than a permutation space from an unrestricted 45*P*2 security system.

**Fig. 6.**
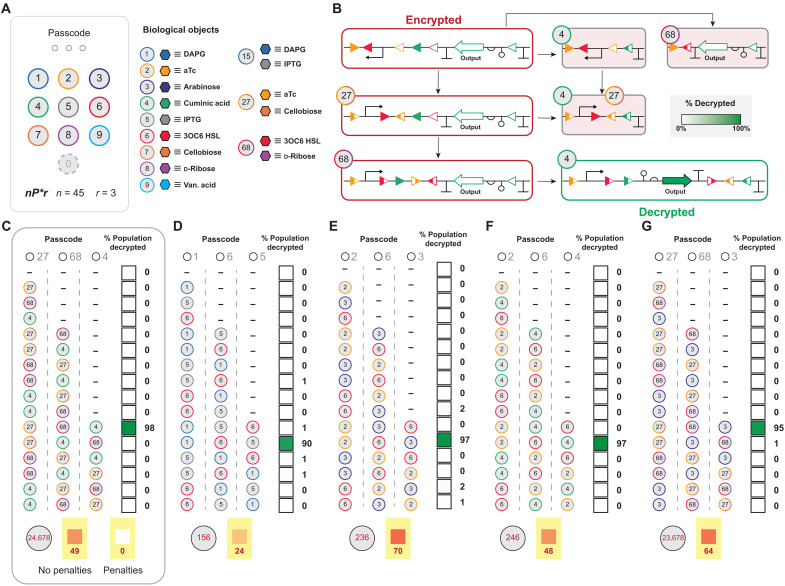
*nP3* biological (genetic) permutation locks. (**A**) Expanded biological keypad including two-digit objects. (**B**) Schematic of an *nP3* permutation lock, encrypted using 3 sets of *att* sites with decryption instructions shown—i.e., the encrypted DNA sequence is decrypted via premutation 27,68,4. Select incorrect permutations of said authentication code results in the deletion of the genetic asset as shown. The inset grey box with “% decrypted” is the scale used in C-G. (**C** to **G**) Five *nP3* permutation locks were engineered, two encrypted DNA sequences requiring only one-digit permutation entries, and three requiring both two-digit and one-digit entries. [(C) to (G)] Bottom: gray circles with red numbers represents trivial-decryption inputs, red box with yellow background are results when all objects were introduced concurrently. (C) Bottom: Trivial decryption was mitigated by incorporating advanced penalties (right yellow box); details are given in Fig. 7. Source data are provided in table S1. Data represent the average of *n* = 6 biological replicates.

To illustrate the application of expanded authentication codes, the blue team designed encrypted DNA sequences that require three entry (*r* = 3) decryption, in the context of the 45P*3 capacity. The general design of the encrypted DNA sequence is given in Fig. 6 (A and B). As with previous designs, each encrypted DNA sequence contains penalties that result in deletion of critical components of the asset upon incorrect entries. The blue team engineered a total of five encrypted DNA sequences with *nP*3 permutation authentication (Fig. 6, C to G), i.e., (i) three encrypted DNA sequences that can be decrypted by one-digit object permutation entries and (ii) two encrypted DNA sequences that require permutations that contain both one-digit object and two-digit object entries. In turn, the blue team tested ~0.2% of the permutation space (i.e., assuming a permutation space of 25,317 sequences) for each security system, including the correct decryption code. In all cases, each encrypted asset is decrypted at a level greater than 94% via the correct three entry chemical authentication code. Moreover, each *nP*3 security system displayed definitive OFF states (i.e., between 0 and 2% population decryption, under the conditions tested; see Materials and Methods) with incomplete or incorrect authentication for the tested portion of the search space.

### Trivial-decryption testing and mitigation

Next, the blue team tested each of the two entry systems from Figs. 3 and 5 for trivial decryption via introducing all cognate objects concurrently (see Fig. 3C for illustration). In all cases, each security system was trivially decrypted, which is anticipated given that no measures are designed to mitigate infiltration of the asset via introducing all objects simultaneously. However, the degree of trivial decryption for two entry systems varied from 11 to 98%. The blue team posited that the differences in trivial decryption can be attributed to asymmetry in recombination kinetics (fig. S5), although could not glean a clear design rule to take advantage of this observation. Likewise, all three entry systems were trivially decrypted via the same strategy (Fig. 6, C to G). Notably, the two entry systems experienced a higher level of trivial decryption (averaged at 70%) relative to the three entry systems (averaged at 51%). The blue team attributed the decrease in encrypted DNA sequence penetration to the increase in complexity going from four genetic states to a system composed of seven genetic states. If this precept holds, then eliminating system penetration (without additional complementary penalties) would require a system with greater than 20 genetic states. The current encryption limit is predicated on 12 sets of recombinase attachment sites; thus, designing an encrypted DNA register with more than 20 genetic states is certainly possible (*48*).

**Fig. 7.**
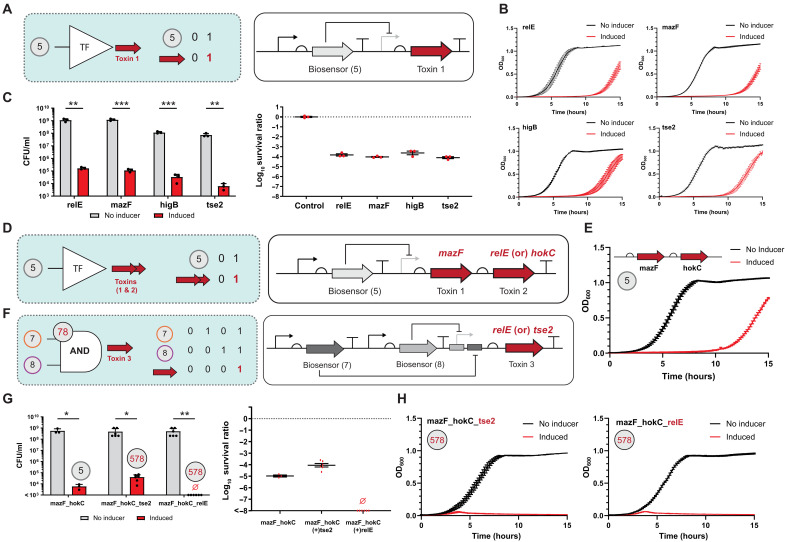
Advanced penalties complementary to *nP*3 permutation locks. Schematics and results of genetic kill switches (**A** to **H**) engineered to be compatible with the encrypted asset given in Fig. 6B. (A) Logic and wire diagram of a genetic kill switch where LacI (I^+^_YQR_, cognate to object #5) regulates the expression of a single toxin (i.e., *relE*, *mazF*, *higB*, or *tse2*). (B) Growth inhibition from single-toxin genetic kill switches. (C) Colony-forming units (CFUs) were measured to assess the performance of single-toxin genetic kill switches. The gray bar represents survival with no inducer, and the red bar represents survival in the induced state via IPTG, cognate to object #5. The log_10_ survival ratio, calculated as log_10_ (CFUs with inducer/CFUs without inducer) is shown. (D) Schematic of bicistron toxin regulation. I^+^_YQR_ regulates the expression of a bicistron toxin set, i.e., toxin 1 = *mazF* and toxin 2 = *relE* or *hokC*. (E) Growth inhibition result for the toxin 1 = *mazF* and toxin 2 = *hokC* bicistron genetic kill switch. (F) Schematic for the regulation of toxin 3 (*relE* or *tse2*) via an AND logic gate, i.e., R^+^_GKR_ (cognate to object #8) and E^+^_KSL_ (cognate to object #7). (G) CFUs and log_10_ survival ratio were measured to assess the performance of genetic kill switches composed of three toxins, and Ø indicates no colony growth. (H) Growth inhibition results for three toxin genetic kill switches are shown: mazF_hokC_tse2 (left) and mazF_hokC_relE (right). Source data are provided in table S1. Data represent the average of *n* = 4 or 6 biological replicates except (C) where *n* = 3. Error bars correspond to the SEM of these measurements. Statistical analysis was performed using two-tailed unpaired *t* tests (*P < 0.05; **P< 0.01; ***P < 0.001).

Rather than expanding the number of recombination states to mitigate trivial decryption, the blue team posited that an additional layer of security could be engineered in the form of penalties that are triggered when undesired combinations of objects are introduced concurrently. Here, the blue team’s objective is to design a system of complementary penalties that are activated upon the concurrent entry of undesired combinations of inputs. At the outset the blue team focused on mapping the #5 biosensor to four different toxins (*68*–*71*) with disparate modes of action (Fig. 7, A to C). Using a serial dilution assay, the blue team observed growth inhibition upon the addition of isopropyl-β-d-thiogalactopyranoside (IPTG), cognate to digit #5, showing a survival ratio of ~10^−4^ for each of the toxins. However, from the growth curve assays, the blue team noticed an escape population for every toxin, which recovered culture growth after ~12 to 15 hours (Fig. 7B). Using a metric of the optical density at 600 nm (OD_600_) at ~0.5, mazF has the slowest escape population (~15 hours), followed by relE (~14 hours), tse2 (~12.5 hours), and higB (~12 hours). This result suggests that mazF is the best single toxin for the intended purposes, whereas higB is the least effective toxin, under the conditions tested.

The blue team posited that a penalty that used multiple toxins to compensate for the neutralization of any single toxin could be engineered. To accomplish this, the blue team designed a digit #5 BUFFER operation mapped to a bicistron that codes for two toxins concurrently (Fig. 7, D and E, and fig. S11). Namely, the blue team designed a bicistron #5 BUFFER gate (i) that codes for mazF + relE and (ii) a second iteration that codes for mazF + hokC. The justification for the additional toxin was that hokC (*72*) offers an additional mode of action that may improve penalty performance. Between the two systems, the mazF + hokC has a better resistance to escape with a cognate decreased survival ratio of 10^−5^ (fig. S11). To further reduce the survival ratio, the blue team designed a three-input penalty system composed of a digit #5 BUFFER gate mapped to the mazF + hokC bicistron, paired with an #78 AND gate to regulate a third toxin (Fig. 7, D to F, and fig. S11). First, the blue team tested the #5 BUFFER bicistron paired with the #78 AND gate mapped to tse2. The mazF + hokC + tse2 trio mitigated escape; however, the survival ratio was still on the order of 10^−4^ (Fig. 7, G and H). Accordingly, the blue team tested the #5 BUFFER bicistron paired with the #78 AND gate mapped to relE. In addition to mitigating escape, the mazF + hokC + relE combination exhibited a survival ratio of 10^−8^, which meets the threshold for containment set by the National Institutes of Health (*73*, *74*). Next, the blue team engineered two additional penalties that induced the deletion of select fitness elements upon incorrect entries, i.e., (i) digit #1 facilitating the deletion of a plasmid selection marker and (ii) digit #3 facilitating the deletion of the replication of origin targeting the plasmid containing the asset (fig. S12). In summary, this resulted in penalties cognate to objects 1, 3, 5, and 78, which are complementary to authentication codes 27,68,4 (Fig. 6C) and 2,6,4 (Fig. 6F). Last, the blue team engineered complete iterations of the security system including all penalties relative to authentication code 27,68,4 (figs. S11 and S13). In turn, the blue team retested the 27,68,4 security system with penalties for trivial decryption (Fig. 6C). Consistent with the design goal, the inclusion of said penalties subdued trivial decryption such that ~0% of the population expressed the asset upon the concurrent exposure to all putative entries, on the basis of the limits set for the detection of GFP as single-cell events (see Materials and Methods).

### Preliminary biohackathon: 4*P*2 red team “code-breaking” security test

The blue team began composing a final security system represented as an *nP*3 encrypted DNA sequence, with a putative search space restricted to an upper limit of 85,140 permutations for *n* = 45. To ensure tractability in the full-scale exercise, the blue team posited an *n* << 45. Before transferring an *nP*3 security system to the red team for the ethical hacking exercise, the blue team supplied the red team with a preliminary 4*P*2 security system. The purpose of the 4*P*2 trial run was to validate the protocol and gray box constraints to be used in the full-scale biohackathon. The blue team developed a 4*P*2 permutation lock with a 7,4 authentication code and object set composed of 1,5,7,4, without additional penalties. In the preliminary 4*P*2 trial run, the red team was supplied with (i) an *E. coli* stock culture containing the encrypted asset, (ii) four undisclosed cognate chemical inputs given as premixed solutions in microcentrifuge tubes labeled with numeric identifiers, and (iii) a protocol outlining the constraints and conditions for the exercise, see methods given in Supplementary Protocol 1. Briefly, the red team was given the length of the permutation string (*r* = 2) and the number of objects represented within the search space (*n* = 4) and a detailed protocol. The code-breaking test was limited to trial-and-error sampling of the search space, i.e., the red team was not permitted to tamper with the system or use any form of DNA sequencing technology to realize the correct authentication code. The threshold for successful decryption of the asset is set at greater than 80% observation of the *E. coli* cell population in the ON state, i.e., constitutively expressing *gfp* via FACS analysis. The justification for this threshold is to test for decryption via the correct passcode in terms of the gray box constraints, opposed to partial decryption of the population by way of trivial decryption or other spurious decryption modalities. The time limit for conducting the preliminary ethical hacking exercise was restricted to a maximum of 14 days. Once completed, the red team was instructed to disclose the numeric passcode and corresponding FACS validation for grading (fig. S14). The red team successfully decrypted the asset via the exhaustive search of all 12 permutations represented in the 4*P*2 search space. Moreover, the red team did not report any obvious issues with the protocol or material transfer process.

### Biohackathon: 11*P*3 red team code-breaking security test

**Fig. 8.**
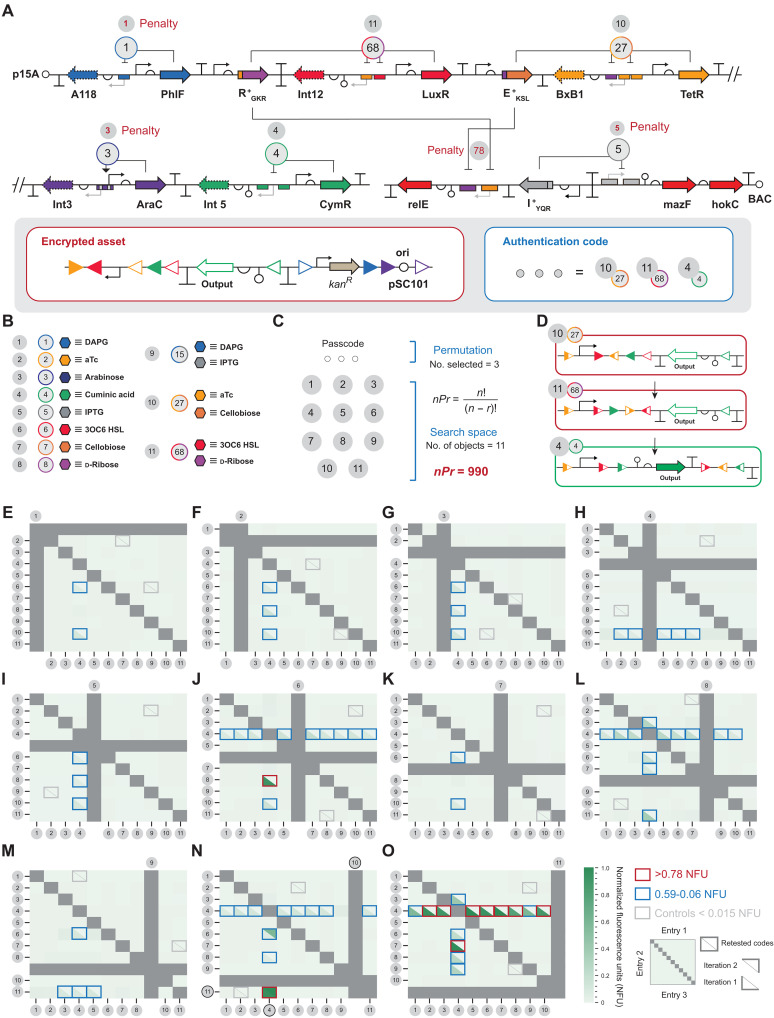
Ethical hacking of a biological asset. (**A**) Schematic of the protected asset provided to the red team for the security challenge. The asset is protected via an 11*P*3 premutation lock with penalties to prevent trivial decryption. (**B** and **C**) Schematic representation of the 11*P*3 permutation space for said exercise with a final search space composed of 990 unique entries. (**D**) Asset decryption shown for authentication code 27,68,4 encoded as 10,11,4. (**E** to **O**) Results from the ethical hacking exercise conducted by the red team. Fluorescence data were collected via plate reader, summarized as entry grids. The top of each grid corresponds to the first entry in the sequence, the *y* axis corresponds to the second entry, and the *x* axis corresponds to the third entry of the permutation. Fluorescence values are shown on a white-green scale; scale bar is given. From the 990 permutations represented in the search space, the red team identified 10 authentication codes that resulted in expression of the asset at normalized fluorescence units (NFU) > 0.78 for each sample, resulting in an ~1% chance of gaining access to the asset by random search. The correct authentication code (10,11,4) was inclusive in the set of 10 authentication codes identified by the red team. Gray boxes denote repeated entries not included in the permutation space. [(E) to (O)] Red, blue, and gray rimmed boxes: The results of the second iteration of 92 authentication codes using a pristine sample (genetically and phenotypically confirmed) resulting in an ~0.2% chance of gaining access to the asset by random search, on par with the intended ~0.1% that was projected from the permutation calculation. Source data are provided in table S1. Data represent the average of *n* = 3 biological replicates.

Next, the blue team transferred the final security system to the red team. Extrapolating from the logistics and restrictions used in the preliminary 4*P*2 exercise, an exhaustive search of a 45*P*3 security system would require ~2500 days to complete using the current protocol and restrictions. Accordingly, the blue team reduced the number of objects *n* by a factor of ~4 resulting in an 11*P*3 search space to increase the tractability of the security exercise, estimating that said security system can be exhaustively evaluated in ~1 month. For this exercise, the blue team selected the 27,68,4 encrypted DNA sequence illustrated in Fig. 6C as the foundation (also see fig. S13). The justification for this selection is threefold: (i) The cognate level 2 encrypted circuit 68,4 (illustrated in Fig. 5F) resulted in relatively high performance and is devoid of substantial system penetration via the entry of incorrect authentication codes. (ii) The cognate 27,4 level 2 encrypted circuit also has strong performance, with the exception of a moderate history related decryption event via 2,7,4 (Fig. 5D). However, given that 27 is the first entry in the permutation string, the blue team posited that the impact of this history related decryption event should not have any notable impact on the exercise. If need be, said issue can be mitigated via adjusting the concentration of the cognate inputs as evidenced in fig. S10. (iii) The entries cognate to the permutation string are complementary to the inputs that activate penalties (Fig. 7, A to H, and fig. S12) and have been shown to mitigate trivial decryption (Fig. 6C). The composed security system is given in Fig. 8 (A to D); for the purpose of the biohackathon, authentication code 27,68,4 is encoded as 10,11,4.

Once the final 11*P*3 security system was generated, the red team was supplied with a protocol (see Supplementary Protocol 2, iteration 1) and a material kit that contained cell stocks with and without penalties, designated as the validation strain and testing strain, respectively. Both security systems required the same authentication code; however, the validation strain contained penalties to prevent trivial decryption of the asset. The justification for using a testing strain and validation strain with synonymous authentication codes is to improve the tractability of the exercise given the substantially slower growth rate of the security system with penalties (fig. S13).

The red team conducted the initial evaluation of the 11*P*3 security system via the testing strain using the same constraints established for the preliminary challenge. Testing conducted by the red team resulted in 10 putatively decrypted systems with normalized fluorescence units (NFU) greater than 0.78 (Fig. 8, E to O, and fig. S15). While the red team did not identify the exact authentication code for the 11*P*3 security system, the correct authentication code was contained within the 10 preliminary authentication codes identified via the testing strain. Objectively, this resulted in an ~1% chance of gaining access to the asset by random search, opposed to the intended ~0.1% that was projected from the permutation calculation. While not completely successful, this is a notable accomplishment toward demonstrating the system cannot be easily decrypted. Rather than proceeding to the validation portion of the exercise, data were transferred to the blue team to reconcile why the security system did not function perfectly.

### Biohackathon iteration 2: Blue team analysis versus red team code-breaking security test

The blue team analyzed the data cognate to (i) the 10 authentication codes that resulted in major events corresponding to greater than 0.78 NFU, (ii) the 40 authentication codes that resulted in moderate events in the range of 0.59 to 0.10 NFU, and (iii) the 20 authentication codes that resulted in minor events corresponding to 0.09 to 0.06 NFU (Fig. 8, E to O). The blue team concluded that three factors affected the 11*P*3 security system and contributed to imperfect authentication and performance (fig. S15). The first factor is identified as an unpremeditated inversion of the set of attachment sites cognate to the first entry object #27 (encoded as object #10) most notably evidenced in the matrix given in Fig. 8O. Said perturbation to the system reduced the permutation string from a three-entry system to a two-entry system via eliminating the requirement for the first entry. The second factor is population heterogeneity in the initial growth culture at the point of the security challenge, resulting in variable levels of protein expression observed in putatively decrypted systems. Last, the third factor is leaky recombinase expression cognate to object #68 (encoded as object #11). The blue team posited that issues related to the first two factors can be resolved via adjustments to the sample preparation and improvements to the protocol, whereas the third factor requires modest redesign of the security system. Overall, the blue team posited that addressing factors 1 and 2 should have the greatest impact on improving the performance of the security system.

Accordingly, the blue team prepared new samples for the testing strain and validation strain and verified samples of each system genotypically to ensure that the materials used for the security challenge are pristine. Rather than have the red team repeat the entire exercise, the second iteration focused on retesting the 70 events that resulted in NFU between 1.00 and 0.06, plus 22 randomly selected authentication codes that did not decrypt the asset as controls (Fig. 8, E to O, and fig. S16). The red team evaluated the assigned 92 authentication codes via the testing strain using a revised protocol that focused on mitigating culture heterogeneity (see Supplementary Protocol 2, iteration 2). Retesting of said authentication codes affirmed the blue team’s supposition regarding factors 1 and 2. From the retested major events, only 1 of the 10 authentication codes decrypted the asset; notably, this is the correct authentication code. In kind, all 22 control authentication codes do not decrypt the asset. From the retested moderate and minor events, 59 of the 60 authentication codes failed to decrypt the asset, the exception being permutation 10,6,4 (however, not 10,8,4) affirming the blue team’s supposition regarding factor 3. Recall, the third factor is leaky recombinase expression cognate to object #68 (encoded as object #11). Notably, the blue team posited that component #8 of object #68 is primarily responsible for the leaky expression of the cognate recombinase, given that, in several cases, the system can be induced with object #6 exclusively. In turn, the red team evaluated the 10,11,4 authentication code that resulted in the sole major event, in addition to authentication code 10,6,4, via FACS analysis using the testing strain. The results from the population analysis were consistent with the plate-reader data, i.e., 10,11,4 and 10,6,4 resulted in ~100 and ~60% decryption of the population, respectively (also see fig. S16). An assessment of said authentication codes using the validation strain mirrored the results observed in the testing strain. The blue team regarded the second iteration of the security challenge as a success. Objectively, this resulted in an ~0.2% chance of gaining access to the asset by random search which is on par with the intended ~0.1% that was projected from the permutation calculation.

## DISCUSSION

Here, we present an intrinsic (genetic level) biological security technology inspired by cybersecurity principles applying a blue team and red team to respectively design-build and test genetic-level security systems. In this study, we present myriad prototypes of said technology using a generalizable asset in the form of a single GoI composed as an expression sequence. The final encrypted DNA sequences used in the security exercise were highly composable from the simpler optimized parts, with forecasted performances that are fateful to the intended design goals. Our technology is designed to maximally protect nondisclosed DNA sequences and cognate parts and can be applied complementary to biocontainment strategies. A critical part of the development of our security system is objective assessment of modes of failure, the identification of exploitable security flaws, and cognate security improvements. Likewise, we structured the discussion to address modes of failure, improvements, and the requirements for application through the lens of the constraints introduced in Fig. 1.

At the outset, we conjectured that direct discovery of the asset by sequencing can be mitigated. However, we did not provide an illustration of said mitigation strategies. In kind, there are likely myriad failure modes and security flaws that we have not discussed. Analogous to cybersecurity developments, the objective of this study and exercise is not to engineer a perfect security system at the outset. Rather, the goal is to develop a platform technology that allows for iterative improvements that are responsive to technological changes over time. In other words, the critical question is: Does the present technology represent a scalable and adaptable security system that can be iteratively improved? We believe that the answer is categorically yes, and we plan to systematically demonstrate improvements to our security platform technology in future biohackathons, which will include strategies to mitigate DNA sequencing. Accordingly, the disclosure of certain mitigation strategies and modes of failure is beyond the scope of this work.

The second constraint that we articulated for this security challenge is that the DNA sequence and primary structure are not disclosed. The obvious question is whether this constraint is practical. For many commercially available enzymes expressed in *E. coli* (and other chassis cells), the DNA sequences are not disclosed. For example, New England Biolabs Inc. (NEB) sells more than 265 restriction enzymes, without disclosure of the specific amino acid sequences. Accordingly, the nondisclosure constraint has practical relevance for existing and future intellectual property and cognate valuable cells. We qualify this by stating that full deployment will require a solution to mitigating DNA sequencing and the like.

The final constraint that we placed on this exercise was that all objects on the keypad are orthogonal and the chemistry and cognate mechanisms used to record events are not disclosed. Regarding the latter point, the concealment of the chemistry and cognate mechanisms of the object space and encryption details will require some form of DNA sequencing mitigation, paired with obfuscation of the input identities. In addition, objectively accomplishing full deployment of every object on a given keypad would require mapping each one-digit (BUFFER gate) and two-digit (AND gate) biosensing operation to an orthogonal recombinase. Now, there are 20 to 25 orthogonal recombinases known in bacteria and 30 to 40 across all organisms (*47*, *48*, *58*–*60*), which sets the technical limit for the object space without engineering new protein functions. In practice, building a system with all objects explicitly represented in a cell is unnecessary with the application of technologies to mitigate DNA sequencing, paired with the ability to obfuscate chemical entry details. In other words, without said details, the hacker would be forced to navigate a near infinite search space.

As deployed in this study one-digit and two-digit objects that overlap can only be used in the same authentication sequence with restrictions. Namely, one-digit objects must occur before cognate two-digit objects in a permutation string to avoid the unintended activation of a one-digit object with two-digit object engagement. For example, as applied permutation code 1,12 can be regarded as discrete, however, 12,1 of the same design would result in the concurrent engagement of 12 and 1 upon the first entry. Notably, we have developed discrete protection strategies (*47*, *49*) that should enable the utilization of one-digit and two-digit objects in a permutation string without said restrictions. Accordingly, we have the ability to realize an orthogonal biological keypad that can be operated via discrete one-digit and two-digit objects (concurrently insulated from disclosure). Note, protection and mitigation strategies will be applied in future biological keypad designs.

In addition to the above, a solution to detection limits is required to deploy this technology in a practical application. Namely, in this exercise, we used a high detection limit (approximately greater than 80% population decryption), which is not practical for most applications. We posit that increasing the genetic state space as it relates to the encryption level can be leveraged to reduce unintended decryption of a given asset objectively to zero. In other words, this would require increasing the permutation string to a value of *r* greater than three, allowing success to be defined in terms of a binary response opposed to setting the threshold to a nonzero population decryption value.

Another point of consideration is the use of GFP as a proxy for the asset. Typically, biological security in the context of biocontainment is measured in terms of cell viability or survival ratio. The key advantage of using GFP and FACS is that this modality directly reports on the state of the asset, which is not necessarily true when measuring cell viability or survival ratio. However, the major disadvantage of using GFP and FACS as the reporting modality under the conditions tested (see Materials and Methods) is that the detection limit is not necessarily low enough for some applications. In terms of our current workflows, the detection limit is an adjustable parameter that can be modified (to any threshold in principle) using our existing protocols. Moreover, when applied, most genetic assets will not be as easy to detect as GFP, which is advantageous for the deployment of our security technology.

While we present our security platform in the context of protecting a single GoI, we posit that we can concurrently protect more than one GoI (or DNA sequence cognate to noncoding RNA) contained within a single cell using an analogous strategy. In addition, the recombinases and biosensors used in this study can be regarded as universal; thus, many (if not all) operations can be deployed in other cells from different phyla (*75*, *76*) and kingdoms (*77*, *78*). Noting that the universal deployment will require validation of biosensor orthogonality to native processes, testing for toxicity within a given chassis cell, and the ability to transport ligands to the point of sensing in vivo.

This study represents a paradigm shift in the security of valuable cells, marking a transition from physical and operational security measures to informational security intrinsic to a genetic asset. With constraints (Fig. 1), our current technology results in a 0.001% (~1 in 85,000) chance of gaining access to the unencrypted sequence by random chance, assuming that the user has access to the chemical search space. Without access to the chemical search space, access to the asset is virtually impossible by random search. In addition to a shift in security archetype, our biological security platform technology introduces an important modality for testing, learning, and improving intrinsic security measures in the context of simulated unauthorized access via a biohackathon.

## MATERIALS AND METHODS

### Bacterial strains and media

Bacterial strains used in this study were NEB 10-beta [Δ*(ara-leu)7697 araD139 fhuA ΔlacX74 galK16 galE15 e14-*ϕ*80dlacZΔM15 recA1 relA1 endA1 nupG rpsL (Str^R^) rph spoT1* Δ*(mrr-hsdRMS-mcrBC)*; NEB] for cloning and assay and TransforMax EPI 300 [*F-mcrA* ∆*(mrr-hsdRMS-mcrBC)* ϕ*80dlacZ∆M15 ∆lacX74 recA1 endA1 araD139 ∆(ara-leu)7697 galU galK* λ*-rpsL nupG trfA dhfr*; VWR] for bacterial artificial chromosome (BAC) amplification. The general culture of *E. coli* strains was performed in LB Miller Medium (Fisher Scientific) at 37°C with shaking. Super Optimal broth with Catabolite repression medium (Fisher Scientific) was used for recovery, and LB Miller Agar (Fisher Scientific) was used for plasmid selection for transformation. Assays were performed in M9 minimal media [Na_2_HPO_4_ (6.8 g/liter), KH_2_PO_4_ (3.0 g/liter), NaCl (0.5 g/liter), and NH_4_Cl (1.0 g/liter)] supplemented with 2 mM MgSO_4_ (MilliporeSigma), 100 μM CaCl_2_ (MilliporeSigma), 0.2% w/v casamino acids (VWR Life Sciences), and 0.4% (w/v) glucose.

### Chemicals

The following chemicals were used as inducers: 2,4-diacetylphloroglucinol (DAPG; Acros Organics), anhydrotetracycline HCl (aTc; Alfa Aesar), l-arabinose (l-ara; Carbosynth), cuminic acid (cumin. acid; Sigma-Aldrich), IPTG (MilliporeSigma), 3-oxohexanoyl-homoserine lactone (3OC6 HSL; Sigma-Aldrich), cellobiose (Arcos Organics), d-ribose (Arcos Organics), and d-fucose (Carbosynth). The final concentrations used for each inducer were DAPG, 25 μM; aTc, 100 ng/ml; l-ara, 5 mM; cumin. acid, 100 μM; IPTG, 10 mM; 3OC6 HSL, 100 μM; cellobiose, 10 mM; d-ribose, 10 mM; and d-fucose, 10 mM. The following chemicals were used as antibiotics: chloramphenicol (25 μg/ml; VWR Life Sciences) and kanamycin (35 μg/ml; VWR Life Sciences).

### Cloning and plasmid construction

All primers were synthesized by Eurofins Genomics. Gene fragments were either amplified from previous studies or synthesized by IDT. Plasmid constructs were created using Gibson assembly and Golden Gate assembly (*79*). General cloning was performed by inverse polymerase chain reaction (PCR) on a C1000 Touch Thermal Cycler (Bio-Rad) followed by Kinase, Ligase and DpnI (KLD) reaction. Q5 polymerase (NEB) was used for PCR, KLD Enzyme Mix (NEB) and KLD Reaction Buffer (NEB) were used for KLD, NEBuilder HiFi DNA Assembly Master Mix (NEB) was used for Gibson assembly, and T4 DNA ligase (NEB), and Bsa I–HFv2 restriction enzyme (NEB) were used for Golden Gate assembly. All DNA constructs were purified via miniprep (Omega Bio-Tek) and verified by Sanger sequencing performed by Eurofins Genomics. A summary of all plasmids used in this study is given in fig. S17.

### Plasmid constructs

Recombinases and transcription factors were harbored in pLacI (Novagen) backbone with p15a origin of replication and chloramphenicol resistance plasmid. The output circuit plasmids containing the variable attachment sites were constructed in pZS*22-sfGFP plasmid reported by Richards *et al.* (*80*) with pSC101 origin of replication and kanamycin resistance. Toxin-regulation circuits were cloned in BAC plasmid [a gift from J. W. Lee (POSTECH)] containing carbenicillin resistance. They were cloned in engineered NEB 10-beta with six additional transcription factors (PhlF, TetR, LacI, CymR, VanR, and LuxR) integrated for efficient cloning with toxin and preventing the gene mutation and escape population through SOS system. Plasmid maps were provided in fig. S16.

### Recombinase library construction and screening

To tune the recombinase performance, the recombinase libraries were constructed with inducible promoters on the basis of biosensor performance, RBS libraries, start codon degeneracy, and degradation tag libraries of recombinases to make various recombination strengths to test the optimal recombination level with paired biosensors. RBS libraries were designed in RBS Calculator (De Novo DNA). Then, recombinase library constructs were cotransformed with corresponding output circuits. Ninety-six variants were picked and cultured in 200 μl of LB medium with antibiotics in clear flat-bottom 96-well plate (Corning) sealed with a Breathe-Easier membrane (Electron Microscopy Sciences). Then, variants were followed by recombinase memory assay and FACS analysis (see below). The variants that passed the optimal recombination criteria were isolated and the recombinase, and transcription factor plasmids were extracted by PCR and analyzed by Sanger sequencing. Last, variants were retested using the same recombinase memory assay.

### Recombinase memory assay

Recombinase-transcription factor pair plasmid and relevant output circuit plasmid were cotransformed and plated on LB agar with corresponding antibiotics (chloramphenicol and kanamycin). Then, six different colonies were randomly picked and cultured in 200 μl of LB medium with antibiotics in clear flat-bottom 96-well plate (Corning) sealed with a Breathe-Easier membrane (Electron Microscopy Sciences). After 8 hours of growth in shaker (Thermo Fisher Scientific MaxQ 4000) at 300 rpm at 37°C, cultures were diluted 1:200 into 200 μl of M9 minimal medium with antibiotics and with and without the inducers for corresponding recombinase and transcription factor pairs. Plates were sealed with a Breathe-Easy membrane (Electron Microscopy Sciences) and grown for 12 hours, then diluted 1:200 again into fresh M9 minimal medium with the same antibiotics and inducer conditions, and grown for an additional 12 hours. Cultures were diluted 1:200 into 200 μl of M9 minimal medium containing no inducers for memory and grown for 12 hours. After this, the cells were diluted 1:50 into phosphate buffer with kanamycin (2 mg/ml) to inhibit further protein production. After an hour of incubation at room temperature, cells were analyzed via FACS.

### Recombinase kinetic assay

To measure recombination over time, eight recombinase-transcription factor pairs plasmid and relevant output circuit plasmid were cotransformed and plated on LB agar with corresponding antibiotics (chloramphenicol and kanamycin). Individual colonies were inoculated in 200 μl of LB with chloramphenicol and kanamycin in 96-well plates sealed with a Breathe-Easier membrane and grown for 8 hours at 300 rpm at 37°C. Aliquots were then used to seed fresh 500 μl of minimal medium cultures with the appropriate inducer for a given circuit (1:200 dilution), in deep-well plates (Greiner 780271) sealed with a Breathe-Easy membrane. The inducing cultures were grown for a total of 24 hours. To maintain cells in exponential phase, the inducing cultures were seeded into fresh inducer-containing medium (1:100 dilution) at 12 hours. From the time point at 0 hours, cultures from LB were directly seeded into fresh minimal medium without inducer (1:200 dilution) in 96-well plate and analyzed by FACS. Every 4 hours from the inducing plate, cell cultures were diluted (1:200 dilution) into fresh minimal medium cultures without inducers. These cultures were then grown for 12 hours and analyzed by FACS.

### FACS analysis

Fluorescence analysis was performed with a CytoFLEX S (Beckman Coulter) flow cytometer. Cells were diluted 1:40 into phosphate-buffered saline (PBS) with kanamycin (2 mg/ml) and incubated for an hour at room temperature to inhibit further protein production. Cells were processed at a flow rate of 20 μl/min, gated by forward scatter and side scatter, and measured through the fluorescein isothiocyanate channel for GFP expression. The gating strategy and the gain setting are described in fig. S18. More than 50,000 events were collected for final analysis. All FACS experiments were collected via three to six biological replicates.

### Fluorescent assay

For all objects engineered in this study, we used an iteration of the fluorescence-based microwell plate assay established by Richards *et al.* (*80*). After the recombinase memory assay, the OD_600_ and the fluorescence value were measured by a plate reader (Molecular Devices SpectraMax M2e) using an excitation wavelength of 485 nm and an emission wavelength of 510 nm for GFP and excitation wavelength of 588 nm. Data were analyzed with SoftMax Pro Software (Molecular Devices). Wells containing M9 minimal medium and relevant antibiotics with no cell inoculations were used as blank controls, and the average OD_600_ and the fluorescence of the six blanks for each condition were subtracted from each sample. All fluorescence data were collected via six biological replicates.

### *nPr* permutation lock assay

*nPr* permutation lock assessment was conducted using an iteration of the recombinase memory assay described above. In brief, after 24 hours from the first entry cells were diluted 1:200 into 200 μl fresh M9 minimal medium with the second entry and cultured for another 24 hours with a transferring step at 12 hours. Passaging was continued as required. Last, the cell cultures were grown for 12 hours with fresh media without the inducer at 1:200 dilutions. Next, the cells were diluted 1:50 into PBS with kanamycin (2 mg/ml) to inhibit further protein production. After an hour of incubation at room temperature, cells were analyzed by FACS to determine population percentages.

### Quantification of plasmid and resistant marker deletion efficacy

Postdecryption, cells were induced by DAPG or l-ara to measure the efficacy of deletion. After 24 hours of induction with a passaging step at 12 hours, cells were then diluted 1:200 into minimal medium without kanamycin (minus inducers) and grown for 12 hours. After the final growth period, cells were analyzed by FACS to measure the deletion efficacy of the psc101 plasmid. Colony-forming units (CFUs) were counted for DAPG or l-ara conditions to determine the efficacy of the psc101 plasmid deletion. CFU measurements were performed as described in the cell viability assay (given below).

### Cellular burden assessment

Cellular burden was measured via growth curve kinetics. Cells were transformed with and without engineered plasmids and inoculated in 200 μl of LB with appropriate antibiotics in a 96-well plate sealed with a Breathe-Easier membrane. In turn, cells were grown in a shaking incubator for 8 hours at 300 rpm at 37°C. Resulting cultures were seeded into minimal medium (1:200 dilution) in a 96-well plate, and the OD_600_ was measured every 10 min via a plate reader (Molecular Devices SpectraMax M2e) at 37°C for 16 hours. Cells harboring the security system, security system with penalties, and empty plasmids were tested and compared via relative growth curves.

### Toxin regulation (kill switch) assessment

Regulated toxin circuits were constructed in BAC plasmids. To test toxin efficacy, NEB 10-beta competent cells were transformed with the BAC and plated on LB agar with carbenicillin. Individual colonies were inoculated in 200 μl of LB with carbenicillin in a 96-well plate sealed with a Breathe-Easier membrane. Cultures were grown in a shaking incubator for 8 hours at 300 rpm at 37°C. In turn, cells were seeded in minimal medium (1:200 dilution) in a 96-well plate with and without the cognate inducer. Postseeding, the OD_600_ was measured every 10 min for 16 hours via a plate reader (Molecular Devices SpectraMax M2e) at 37°C to determine toxin efficacy.

### Cell viability toxin (kill switch) assessment

To determine cell viability for a given toxin, precultured cells in LB from the toxin regulation assessment were diluted into minimal medium with carbenicillin to an OD_600_ of 0.2. Resulting cultures were serially diluted into sterile PBS with and without cognate inducers and were used to measure the viable CFUs. CFUs were counted by plating 10 μl of serially diluted cultures onto LB agar with the relevant antibiotics and incubated at 37°C overnight. The fraction of cell viability was calculated as the ratio of CFUs measured from induced PBS to the CFUs measured from the uninduced PBS.
