# Supplementary material for: Protecting cells at the genetic level and simulating unauthorized access via a biohackathon
Source: Sci Adv. 2026 Apr 1;12(14):eaeb8556. doi: 10.1126/sciadv.aeb8556 (PMC13041761; doi:10.1126/sciadv.aeb8556)
Supplement: Supplementary file 1 — Figs. S1 to S24 Supplementary Text Legends for tables S1 and S2 [file sciadv.aeb8556_sm.pdf]

Science Advances
AAAS

Supplementary Materials for

Protecting cells at the genetic level and simulating unauthorized access via a biohackathon

Dowan Kim *et al.*

Corresponding author: Corey J. Wilson, corey.wilson@chbe.gatech.edu

Sci. Adv. **12**, eaeb8556 (2026)
DOI: 10.1126/sciadv.aeb8556

The PDF file includes:

Figs. S1 to S24
Supplementary Text
Legends for tables S1 and S2

Other Supplementary Material for this manuscript includes the following:

Tables S1 and S2

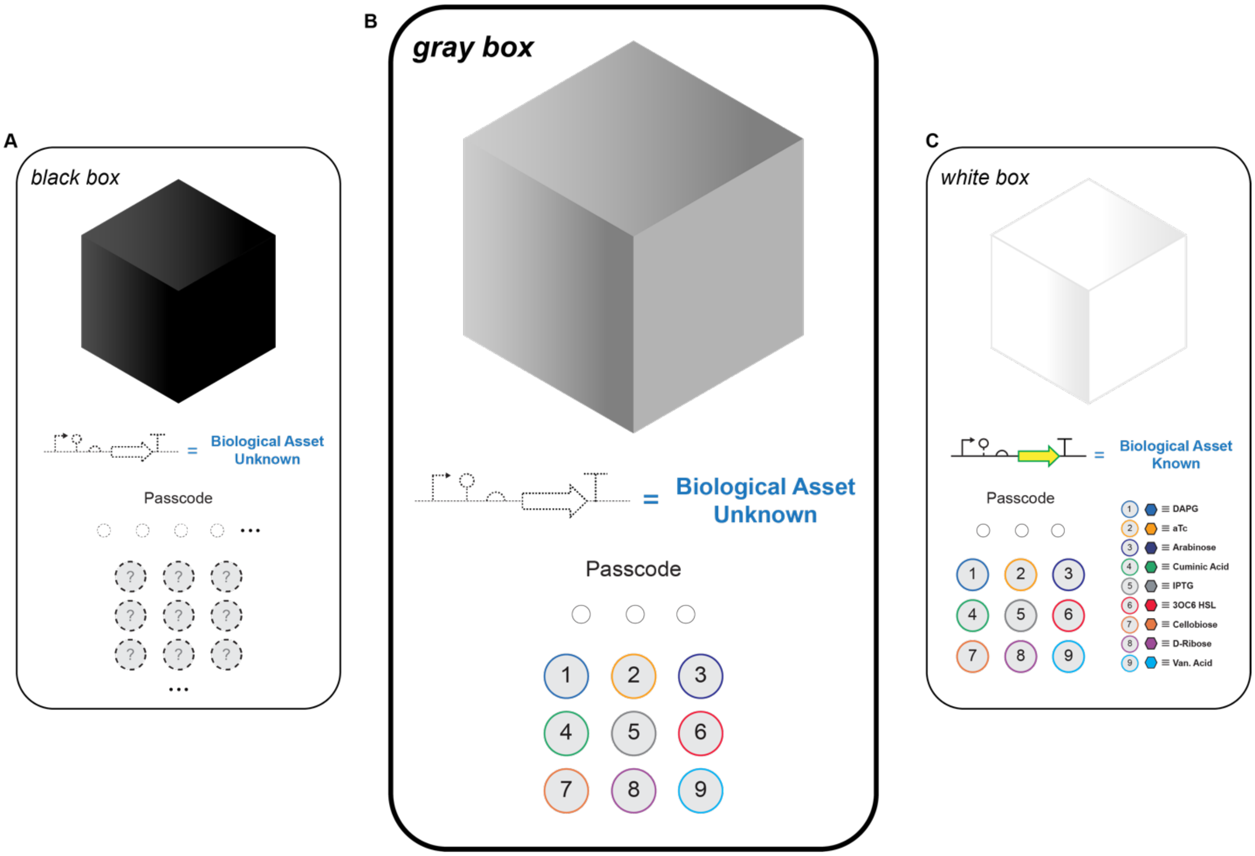

Fig. S1 | Biohackathon penetration testing levels. In cybersecurity, the terms *black box*, *gray box*, and *white box* describe the different levels of knowledge the red team has regarding the system they aim to hack or penetrate. When applied to our Biohackathon – where a biological asset (*i.e.*, an encrypted DNA sequence) is the subject of testing – said terms carry synonymous meanings:

(A) ***black box*** – The red team has no prior knowledge of the biological circuits or mechanisms and must identify weaknesses or vulnerabilities solely through experimental probing. For example, the red team would not have any knowledge of the number of objects n that compose the search space or information regarding the length of the permutation string *r*.

(B) ***gray box*** – The red team is provided with partial information regarding a specific security system to facilitate testing and learning. For example, the red team would be informed of the number of objects *n* that compose the search space and information regarding the length of the permutation string *r*. However, the chemical identities and genetic details would not be disclosed.

(C) ***white box*** – The red team has full access to the genetic designs, *nPr* information, operation details and can extensively learn and probe the entire security system without restrictions.

In our Biohackathon challenge, we implemented gray box testing (also see **Fig. 1**). The red team was provided with partial system knowledge, which included a set of undisclosed chemical inducers with labeled numbers defining the keypad, the length of the permutations string, cell cultures for testing and validation, assay protocols, and information about the output modality of the encrypted asset. However, the exact design details (*i.e.*, DNA sequence information) for the engineered asset and *nPr* details were not disclosed, and restrictions were placed on the methods allowed to ethically hack the system.

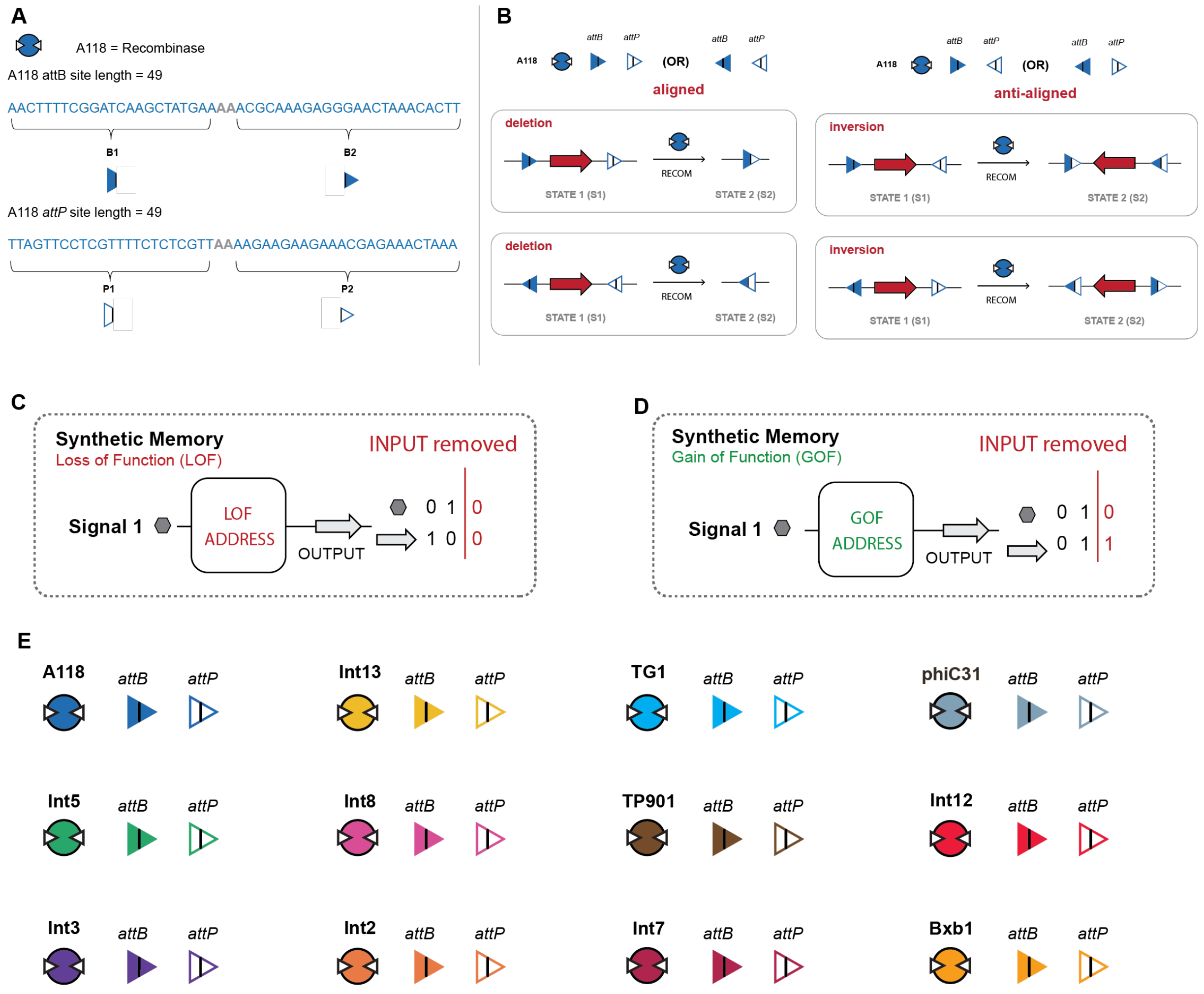

Fig. S2 | Illustrations and iconography for deletion and inversion synthetic memory. (A) A granular description of the set of A118 recombinase attachment sites in the aligned configuration. **(B)** The iconography for the two aligned orientations that result in deletion, and an iconography description of anti-aligned attachment sites that result in inversion for the A118 recombinase. Note: The icon for the recombinase is given as a monomer. The alignment of a set of attachment sites determines the operation – *i.e.*, inversion or deletion, which can be engineered to facilitate **(C)** loss-of-function (LoF) or **(D)** gain-of-function (GoF) inheritable memory. **(E)** The iconography for all recombinases and cognate attachment site used in this study.

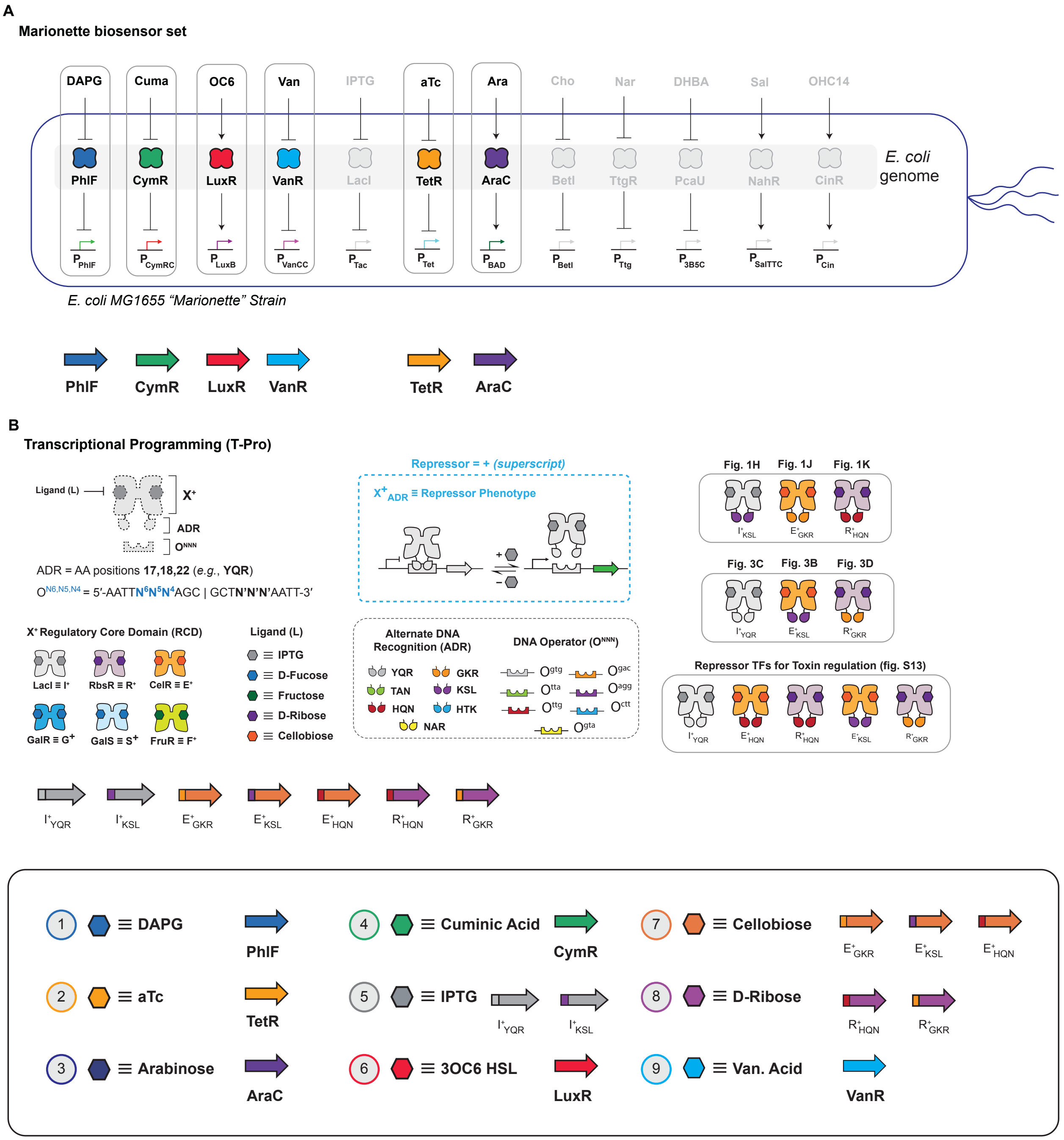

Fig. S3 | Natural and synthetic biosensor selection. In synthetic biology, substantial effort has been dedicated to identifying and developing sets of orthogonal biosensors – abstractly represented as BUFFER logic gates. A notable contribution is the Marionette biosensor set, which consists of natural transcription factors (TFs) that have been engineered for enhanced performance (*i.e.*, dynamic range) (54). This resulted in an orthogonal set of 12 biosensors with improve function **(A)** – *i.e.*, PhlF, CymR, LuxR, VanR, LacI, TetR, AraC, BetI, TtgR, PcaU, NahR, and CinR – all integrated into a single biosensor array within the genome of *E. coli* MG1655. From this set of BUFFER operations we selected PhlF, TetR, AraC, CymR, LuxR, and VanR to construct our biological keypad. Transcriptional Programming (T-Pro) leverages sets of engineered synthetic transcription factors along with cognate sets of synthetic promoters **(B)** that form a system (network) of BUFFER operations (55, 66). T-Pro synthetic TFs have two phenotypes: repressors (denoted with superscript "+") and anti-repressors (denoted

as superscript "A"). Each T-Pro synthetic TF is modularly designed from two key components: (i) a regulatory core domain (RCD), and (ii) an engineered DNA binding domain – denoted as alternate DNA recognition (ADR). The RCDs are abbreviated using a single letter nomenclature – *e.g.*, LacI = I, GalR = G, RbsR = R, CelR = E, and FruR = F. Each RCD is regulated by a cognate inducer – *e.g.*, IPTG for I, D-Fucose for G, D-Ribose for R, Cellobiose for E, and Fructose for F. ADR is predicated on an interaction with engineered DNA operators with the binding specificity determined by residues at position 17, 18 and 22 of an engineer DNA binding domain (*e.g.*, HQN for residues Y17**H**, Q18**Q**, and R22**N**). For example, I^{+}_{HQN} represents a LacI repressor with an HQN DNA binding domain. Said ADR is matched to a unique DNA operator – used to build a synthetic promoter – with cognate substitutions at positions 6, 5, and 4 on the left half-site. A typical DNA operator variant is described as 5′-AATT$N^6N^5N^4$AGC GCTN'N'N'AATT-3′ where $N^{\#}$ represents any nucleotide, and N' is required for the operator to be symmetric. Therefore, we abbreviate the engineered operator as O^{N6N5N4}. Accordingly, DNA binding domain HQN pairs with operator DNA element O^{ttg}. In this study we selected the following T-Pro BUFFER operations $I^{+}_{YQR,}$ I^{+}_{KSL}, E^{+}_{GKR}, E^{+}_{KSL}, E^{+}_{HQN}, R^{+}_{HQN} and R^{+}_{GKR}. which are orthogonal to the selected Marionette biosensor both in terms of input processing and DNA binding functions.

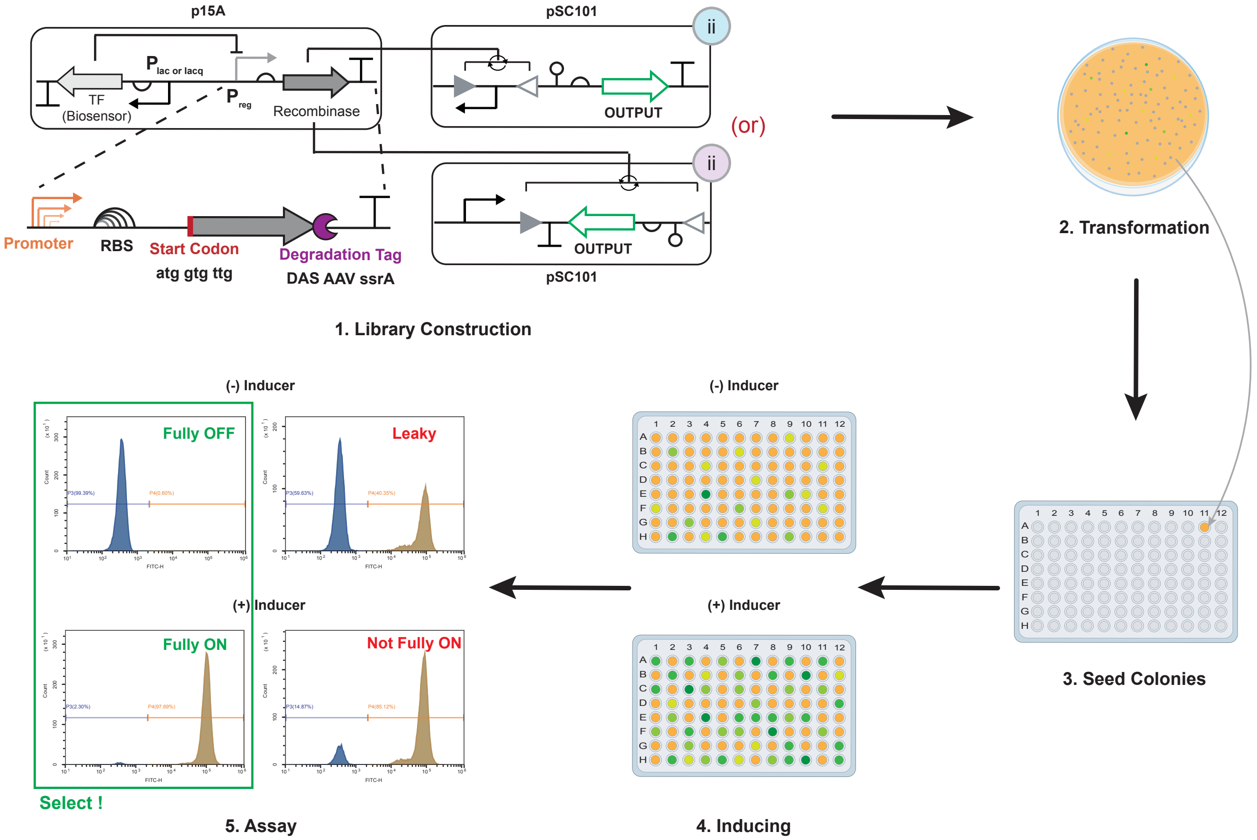

Fig. S4 | Engineering workflow for developing 1-digit objects. The schematic of the workflow for tuning an inducible recombinase as a 1-digit object is shown. A given transcription factor (TF) and cognate recombinase are located on a p15A plasmid. The circuit is designed such that the TF regulates the expression of the recombinase. A library is constructed to optimize the regulation, expression level, and lifetime of the recombinase. The library consists of combinations of inducible promoter variants, RBS elements of variable strength, start codon degeneracy, and C-terminal degradation tags of varying strengths. Each recombinase library is co-transformed with a pSC101 reporter containing a green fluorescent protein (GFP) expression cluster engineered as a gain-of-function memory system. In the workflow, individual colonies are evaluated with and without inducer (cognate to the biosensor) *via* a microwell plate assay and subsequently screened by flow cytometry. Design goals are as follows: (i) minimal recombination in the absence of ligand – targeting < 2% recombination of the population, (ii) maximum recombination in the presence of ligand – targeting > 98% recombination of the population, (iii) minimum to maximum recombination should occur within a prescribed period of < 24 hours.

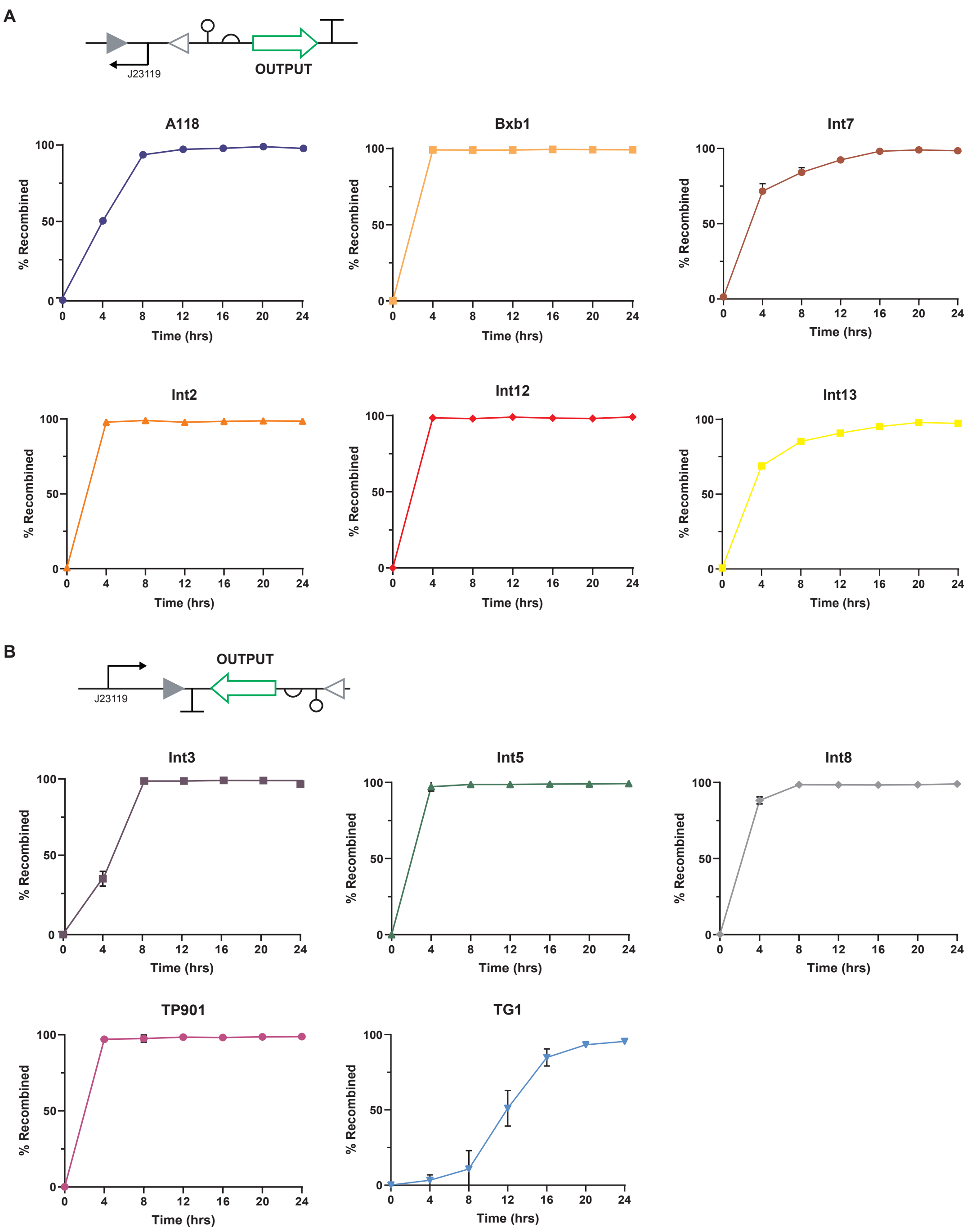

Fig. S5 | Kinetic assessment for 1-digit objects. (A) The recombination kinetics for six 1-digit objects designed as short-frame memory. The recombined population was measured *via* FACS every 4 hours from 0 hours to 24 hours of induction. **(B)** The recombination kinetics for five 1-digit objects designed as long-frame memory. The recombined population was measured *via* FACS every 4 hours from 0 hours to 24 hours of induction. In short-frame recombination pairs cognate to Bxb1, Int2, and Int12 displayed >99% recombination after 4 hours of induction, whereas A118, Int7, and Int13 required at least 12-16 hours of induction to fully recombine the circuits. In long-frame recombination pairs TP901 and Int5 showed more than 99% recombination after 4 hours of induction, whereas Int3 and Int8 required at least 8 hours of induction to fully recombine the circuits. TG1 recombinase showed the slowest recombination kinetics, where it reached >97% after 20 hours of induction. Source data are provided, **table S2**. Data represent the average of n = 6 biological replicates. Error bars correspond to the SEM of these measurements.

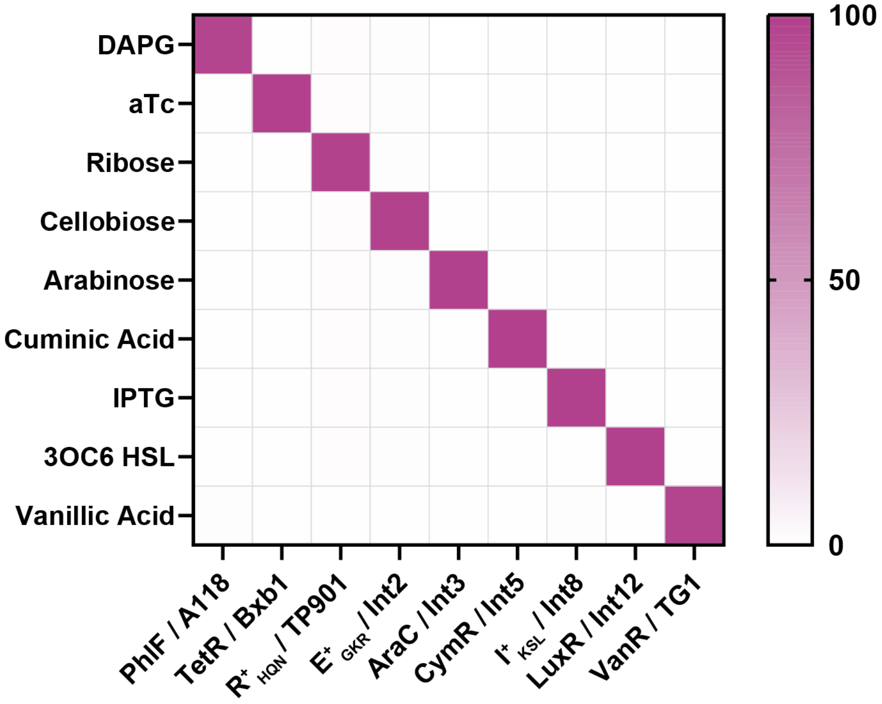

Fig. S6 | Orthogonality testing of the 9 objects represented in the primary keypad. The orthogonality test for $n = 9$ 1-digit objects with the % recombination color labelled in shades of purple scaled from 0 to 100%. Source data are provided, **table S2**. Data represent the average of $n = 6$ biological replicates.

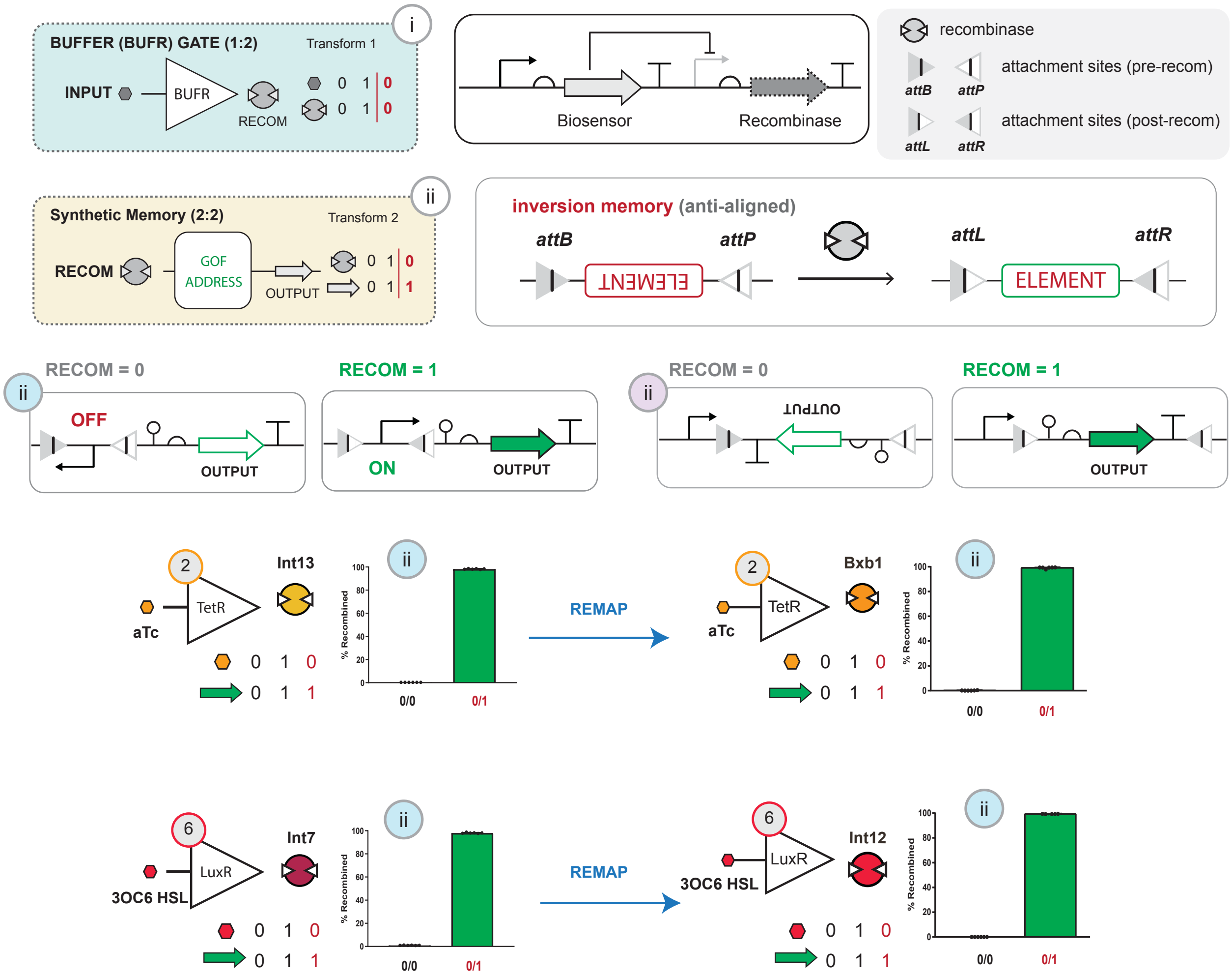

Fig. S7 | Remapping BUFFER gates to additional recombinases (remapped 1-digit objects). (top) The engineering strategy used to construct a 1-digit object. The design requires two operations: (i) a biosensor that regulates a recombinase, abstractly represented as a BUFFER operation, and (ii) synthetic gain-of-function (GoF) genetic memory facilitated *via* the induced inversion of a set of anti-aligned attachment sites (*attB* and *attP*) cognate to the BUFFER regulated recombinase. GoF synthetic memory *via* the induced inversion of a promoter (short-frame memory) is designated as a blue #ii, whereas inversion of a *gfp* expression cassette minus the promoter (long-frame memory) is designated as a purple #ii. (bottom) remapped 1-digit objects are orthogonal in terms of chemical inducers and recombinase biochemistry (also see **Fig. 2**). Gain-of-function memory is permanent and inheritable such that the *gfp* output state persists after the input is removed (denoted by red-colored 0/1), and 0/0 represents the OFF state of the circuit. The percentage of population recombination upon transient induction (0/1) is given as a bar graph for each object. Source data are provided, **table S2**. Data represent the average of n = 6 biological replicates. Error bars correspond to the SEM of these measurements.

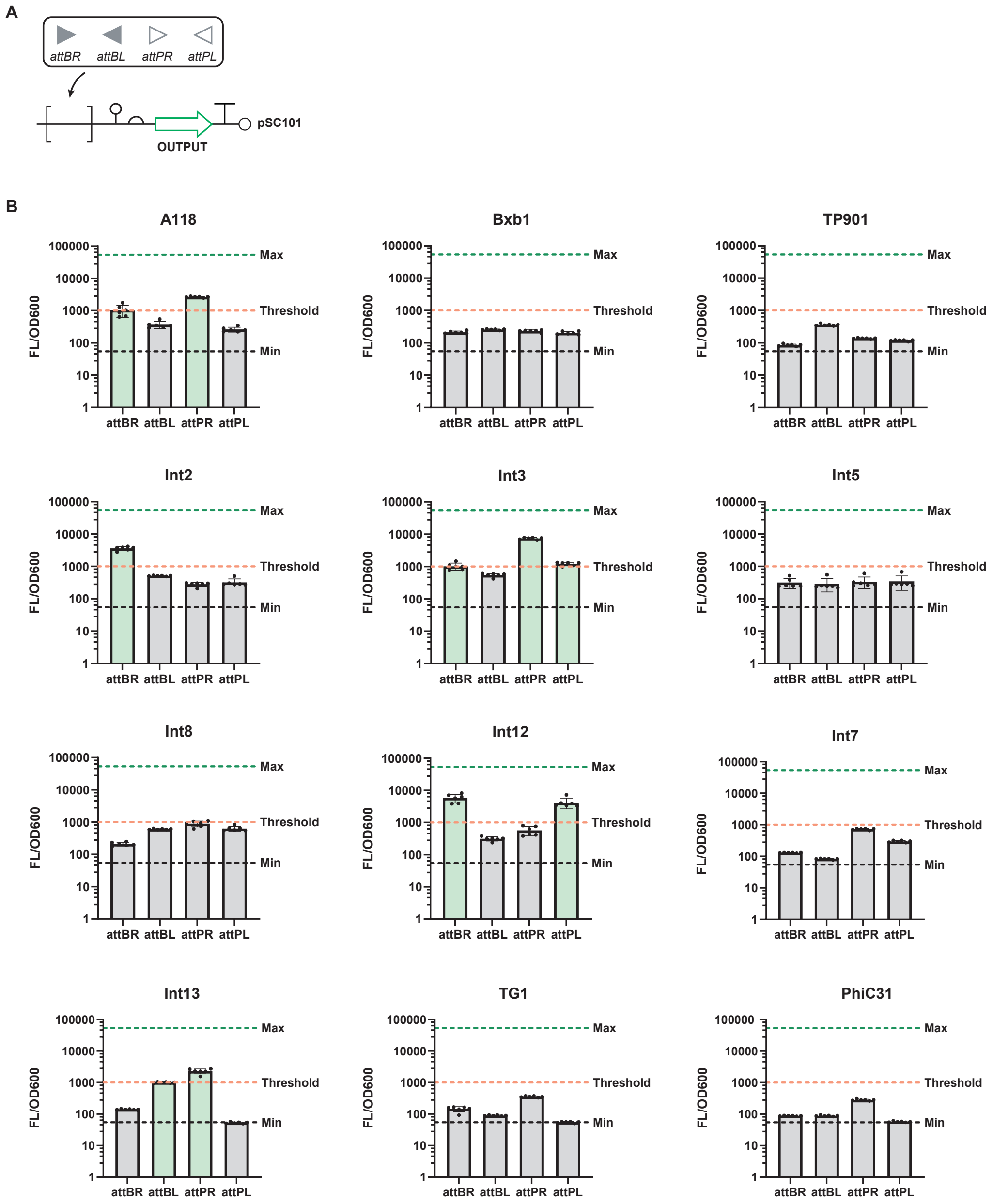

Fig. S8 | Testing cryptic promoter activities in *att* sites for recombinase-based encryption. Attachment sites can behave as weak promoters thus the arrangement of attachment sites is critical to preventing the leaky expression of the gene of interest when it is not induced. To address this issue, we aligned the *attB* and *attP* sites based on their cryptic promoter activities to minimize the basal expression of the output gene by *att* sites upon an incorrect passcode entry. **(A)** The general circuit design for testing cryptic promoter activities of *att* sites. The *att* sites facing the right direction are named *attXR*, and those facing the left direction are named *attXL*. Single *att* sites are inserted upstream of the GFP output gene as pseudo-promoters. **(B)** The expression levels of *att* sites circuits are shown. The value of 1000 FL/OD600 is determined as a threshold. The Max value represents the GFP expression with a J23119 promoter, and the Min value represents the GFP expression without a promoter. Circuits showing GFP expression above the threshold are determined to have cryptic promoter activity. The *att* sites showing no promoter activity are colored gray, while those showing promoter activity are colored light green. Source data are provided, **table S2**. Data represent the average of $n = 6$ biological replicates. Error bars correspond to the SEM of these measurements.

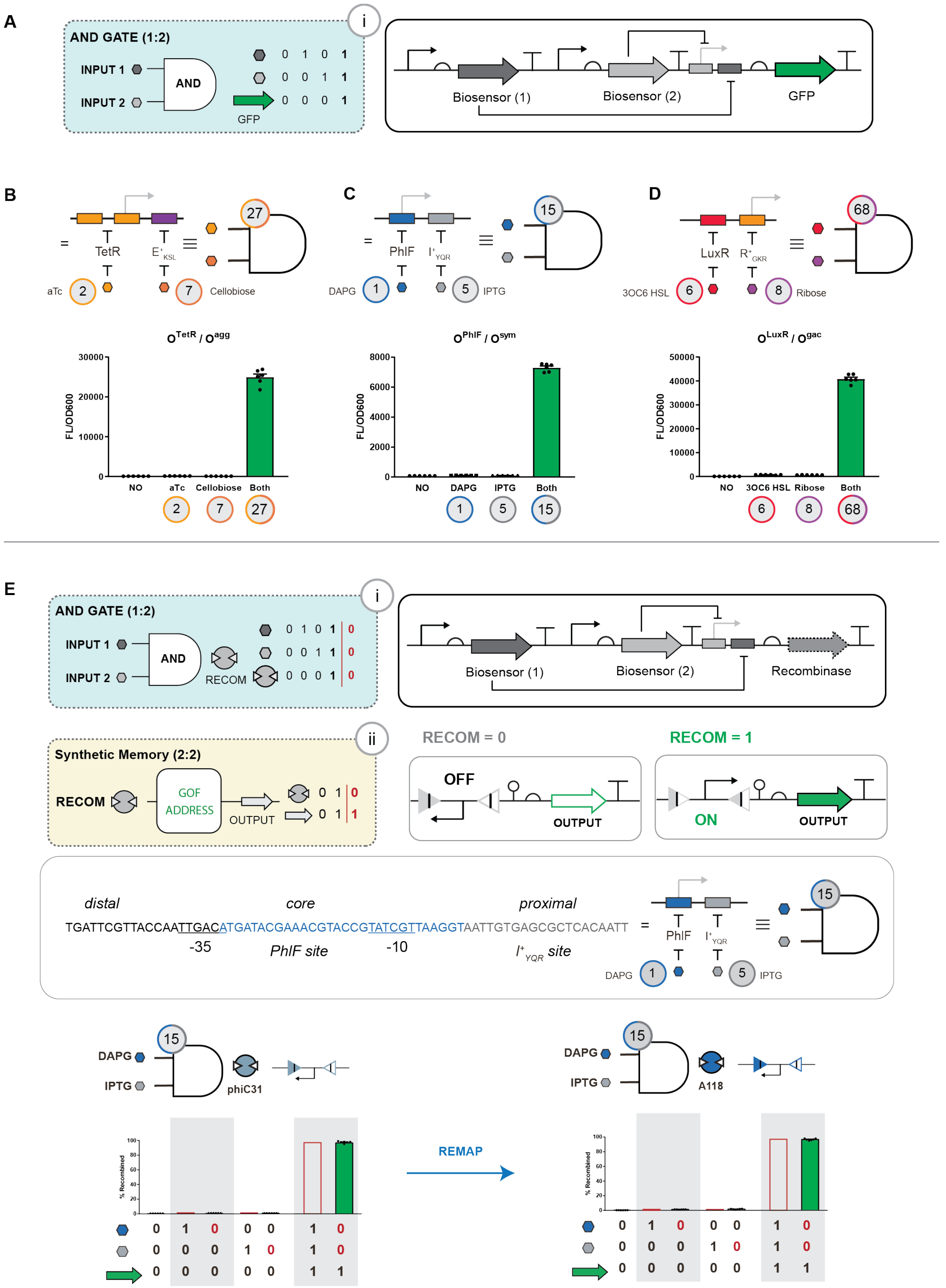

Fig. S9 | Testing hybrid synthetic promoters as simple AND gates & 2-digit object remapping. (A) Schematic illustration for the general design of a 2-digit biological AND gate pairing T-Pro and Marionette biosensors (also see **Fig. 4**). In summary, the mechanism requires the induction of both transcription factors to facilitate the expression of a gene of interest (GoI). **(B)** Hybrid promoter responsive to TetR and E^{+}_{KSL} (#27) is shown, requires aTc and cellobiose to activate expression of a GoI = GFP. **(C)** A hybrid promoter for PhlF and I^{+}_{YQR} (#15) requires DAPG and IPTG to express GFP. **(D)** Hybrid promoter responsive to LuxR and R^{+}_{GKR} (#68) requires 3OC6 HSL and ribose to express GFP. **(E)** The PhlF and I^{+}_{YQR} (#15) hybrid promoter remapped to the A118 recombinase. Source data are provided, **table S2**. Data represent the average of n = 6 biological replicates. Error bars correspond to the SEM of these measurements.

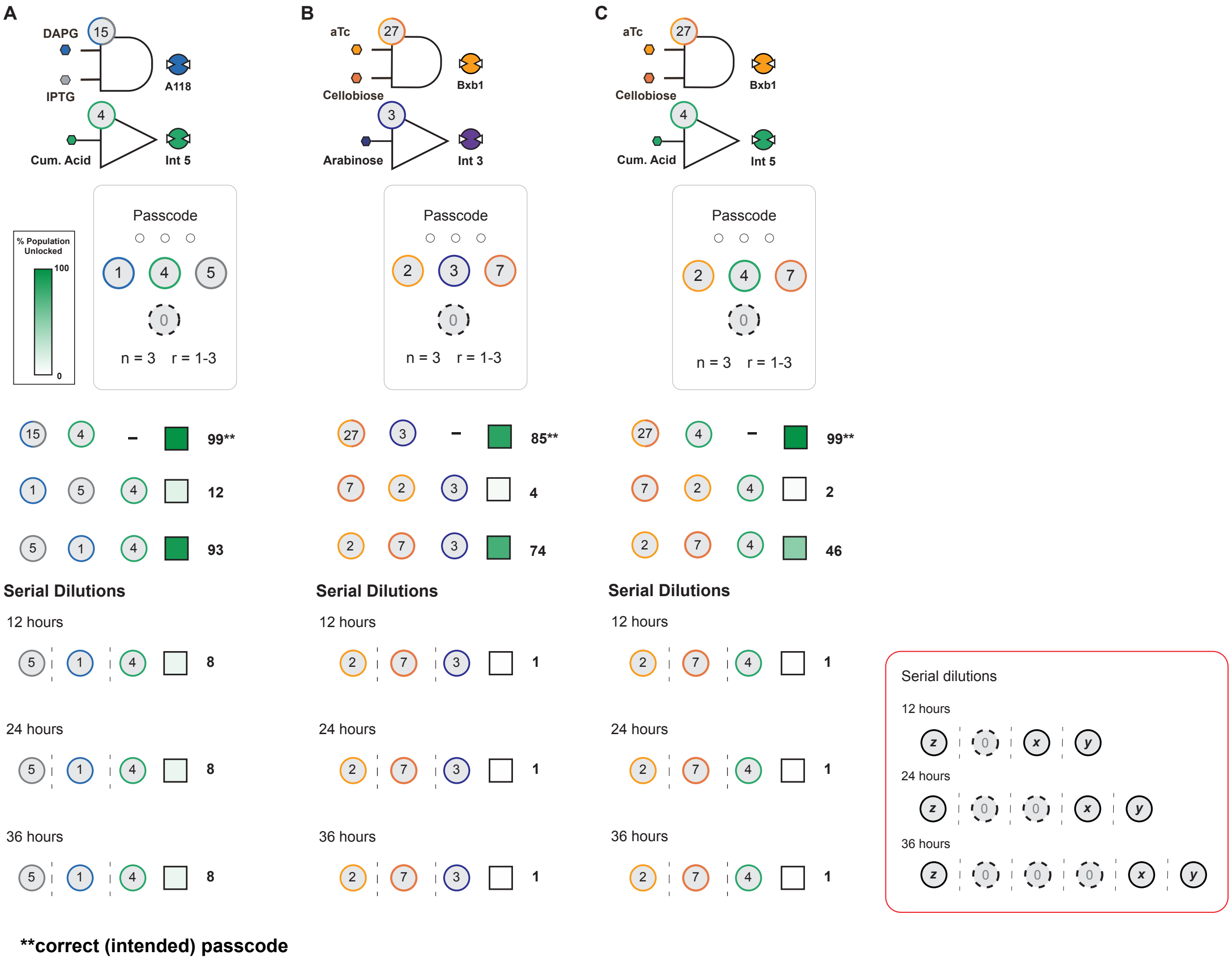

Fig. S10 | Reconciling inexact decryption of *nP2* permutation locks requiring a 2-digit object. Retesting three of the 2-input permutation locks from **Fig 5**, in which the asset was decrypted by an unintended string involved a three-entry sequence in which the first two entries were cognate to the 2-digit object. This was only observed for one of the permutations opposed to both – *e.g.*, 2,7,4 resulted in 46% decryption of the population, whereas 7,2,4 did not result in substantial decryption of the population, such that the correct decryption key is 27,4. The following *nP2* security systems displayed substantial trivial penetration: **(A)** 2-digit (#15) mapped to A118 and 1-digit (#4) mapped to Int5. **(B)** 2-digit (#27) mapped to Bxb1 and 1-digit (#3) mapped to Int3. **(C)** 2-digit (#27) mapped to Bxb1 and 1-digit (#4) mapped to Int5. In each case the correct (intended) passcode is the 2-digit object (*zx*) followed by a 1-digit OBJECT *y*; however, certain 1-digit exclusive permutations decrypted the circuit as described above. The blue team posited that the first input was not sufficiently diluted when the second input was added, allowing the expression of the recombinase assigned to the 2-digit object. To test this, we serially diluted the cells for 12, 24, and 36 hours after the first INPUT (inset red box). The percentage of decrypted population after each serial dilution is represented on a color scale, with the actual percentage noted on the right of the box. Source data are provided, **table S2**. Data represent the average of *n* = 4 or 6 biological replicates.

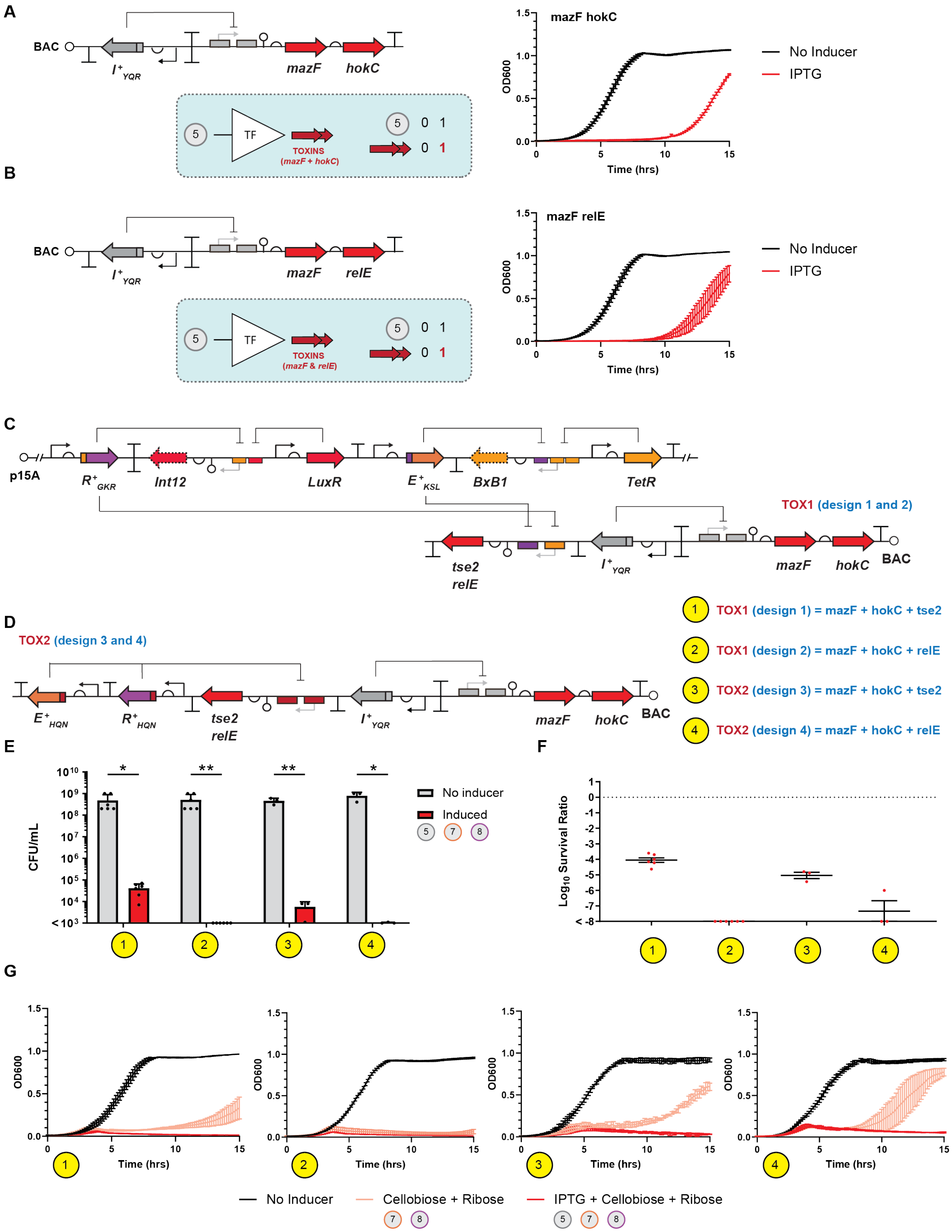

Fig. S11 | Penalties to mitigate trivial-decryption. (A-B) Schematic of genetic kill switches composed of two toxins designed as a bicistron regulated by I^{+}_{YQR} – *i.e.*, cognate to digit #5 (IPTG). The *mazF* toxin is fixed in both designs, such that either the *hokC* or *relE* gene is located downstream relative to *mazF* on the bicistron. Genetic kill switches with *mazF* + *hokC* **(A)** and *mazF* + *relE* **(B)** black data points represent the system without inducer, and red data points represent the system in the presence of digit #5 (IPTG). Between the two systems the mazF + hokC had a better resistance to escape with a cognate decreased

survival ratio of 10^{-5} – relative to single toxin designs given in **Fig. 7 A-B**. Thus, the blue team used the *mazF* + *hokC* set of toxins for designs herein. **(C)** Schematic of the TOX1 circuit design illustrating three toxin regulation. R^{+}_{GKR} (cognate to digit #8) and E^{+}_{KSL} (cognate to digit #7) are used to regulate the expression of a third toxin (*i.e.*, *relE* or *tse2*) as an AND gate. **(D)** Schematic of the TOX2 design for the regulation of three toxins. Here the biosensors cognate to digit #8 (R^{+}_{HQN}) and digit #7 (E^{+}_{HQN}) used to regulate the third toxin (*relE* or *tse2*) have orthogonal DNA binding functions relative to the set used to regulate recombinase expression – *i.e.*, R^{+}_{GKR} and E^{+}_{KSL} see **Fig. 2** for details. **(E)** CFU counts for TOX1 and TOX2 genetic kill switches. The gray bar represents the system without inducer, and the red bar represents the system with inducers – *i.e.*, IPTG (#5), cellobiose (#7), and ribose (#8). **(F)** The $\log_{10}$ survival ratio, calculated as $\log_{10}$ (CFUs with inducer / CFUs without inducers) is shown. **(G)** Growth inhibition results for the bicistron genetic kill switch, combined with *relE* or *tse2* – deployed as a TOX1 or TOX2 design – (i) black lines represent the system without inducer, (ii) light red lines represent the system with cellobiose (#7) + ribose (#8), and (iii) red lines represent the system with IPTG (#5), cellobiose (#7), and ribose (#8). All features considered, design 2 performed the best – *i.e.*, the TOX1 design with mazF + hokC + relE combination exhibited a survival ratio of 10^{-8}, which meets the threshold for containment set by the National Institutes of Health. NOTE: for TOX1 (design 2) the system with cellobiose (#7) + ribose (#8) is more resistant to escape – relative to the system given in **A** – due to leaky expression of the mazF + hokC bicistron; however, after 15 hours said system experiences escape. Whereas the same system with IPTG (#5), cellobiose (#7), and ribose (#8) does not experience escape > 15 hours. Source data are provided, **table S2**. Data represent the average of $n = 6$ biological replicates except designs 3 and 4 **(F)** where $n = 3$. Error bars correspond to the SEM of these measurements. Statistical analysis was performed using two-tailed unpaired t tests (P value: < .05 (*), < .01(**), < .001(***)).

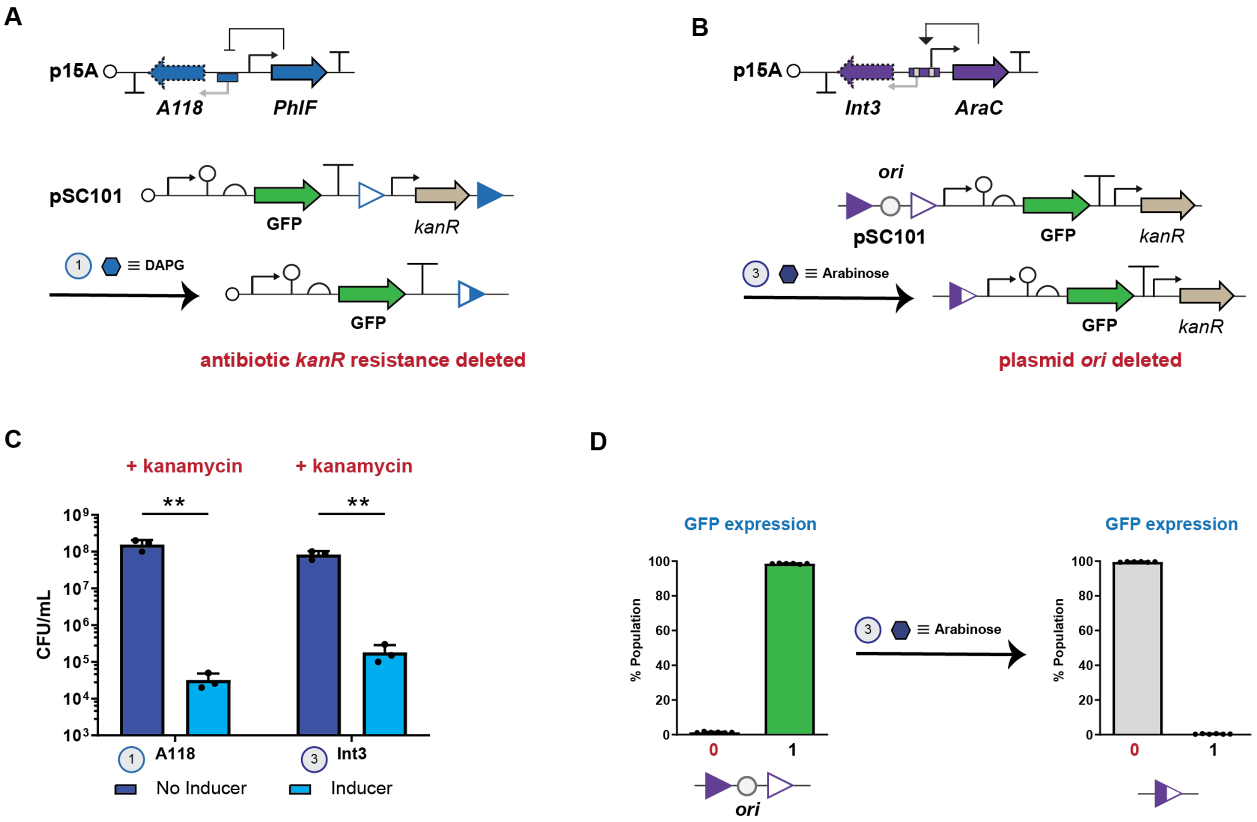

Fig. S12 | Additional penalties to mitigate trivial-decryption. (A) Schematic of the *kanR* gene deletion circuit, induced *via* object #1 – *i.e.*, induction of A118 facilitates the recombination event. Aligned *attB* and *attP* sites cognate to A118 flank the *kanR* cassette; such that, induction *via* object #1 results in the loss of kanamycin resistance on a plasmid that is integral to the security system. **(B)** Schematic of the Int3 origin of replication deletion. Aligned *attB* and *attP* site cognate to Int3 (*i.e.*, object #3) flank the origin of replication, such that, induction *via* object #3 results in the loss of an entire plasmid that is integral to the security system. **(C)** Results for the engineered penalties given in **A** and **B**. measured using a serial dilution method, reported as colony forming units (CFUs) /mL. Both systems were cultured with kanamycin. **(D)** Additional results for penalty **B** shown as the percentage of cells expressing GFP constitutively such that 0 indicates cells not expressing GFP, and 1 indicates cells expressing GFP. Left bar graph is data for cells with the *ori* element; right bar graph are cells post deletion without the *ori* element induced *via* object #3 – *i.e.*, cells no longer replicate the pSC101 plasmid. Source data are provided, **table S2**. Data represent the average of n = 6 biological replicates for **D** and n = 3 biological replicates for **C**. Error bars correspond to the SEM of these measurements.

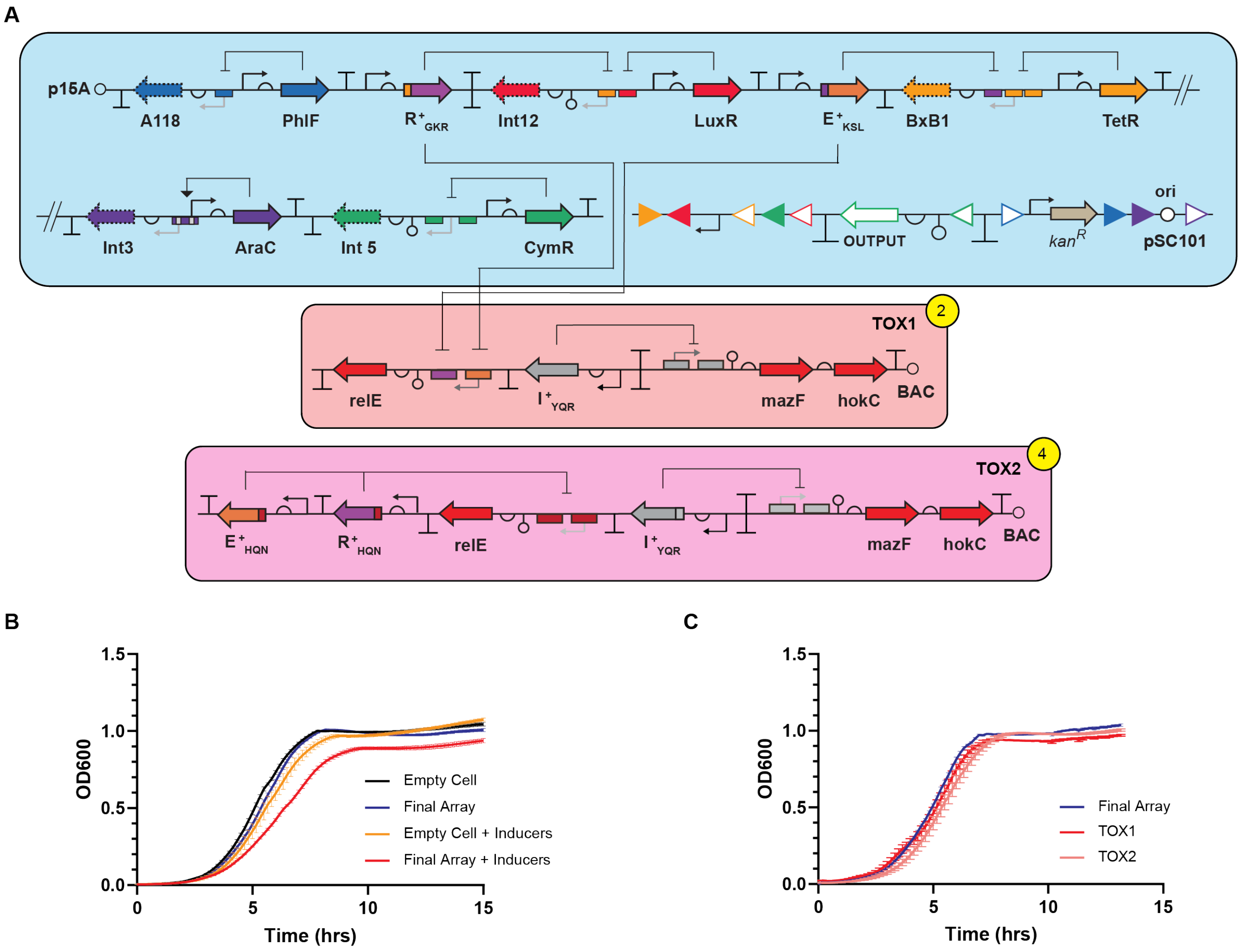

Fig. S13 | Analysis of cellular burden for complete iterations of the security system. (A) Schematic of the final array + fitness penalties with TOX1 (design 2) or TOX2 (design 4) penalties. **(B)** Comparisons of growth curves for (i) empty DH10beta cells, (ii) the final array + fitness penalties without triad toxin penalties, and (iii) said chassis cells with and without inducers. **(C)** Comparisons of growth curves of (i) the final array + fitness penalties, and (ii) the final array+ fitness penalties with TOX1 (design 2) or TOX2 (design 4) penalties. Source data are provided, **table S2**. Data represent the average of n = 6 biological replicates. Error bars correspond to the SEM of these measurements. For the final exercise the blue team selected the 27,68,4 encrypted circuit illustrated in **Fig. 6C** as the foundation. The justification for this selection was three-fold: (i) the cognate level-2 encrypted circuit 68,4 (illustrated in **Fig. 5F**) resulted in relatively high performance – devoid of substantial system penetration *via* the entry of incorrect authentication codes; (ii) The cognate 27,4 level-2 encrypted circuit also had strong performance, with the exception of a moderate history related decryption event *via* 2,7,4 (**Fig. 5D**). However, given that 27 is the first entry in the permutation string, the blue team posited that the impact of this history related decryption event would not have any notable impact on the exercise. If need be, said issue can be mitigated *via* adjusting the concentration of the cognate inputs as evidenced in **fig. S10**. (iii) The entries cognate to the permutation string are complementary to the inputs that activate penalties (**Fig. 7H-O** and **fig. S12**) and have been shown to mitigate trivial-decryption (**Fig. 6C**).

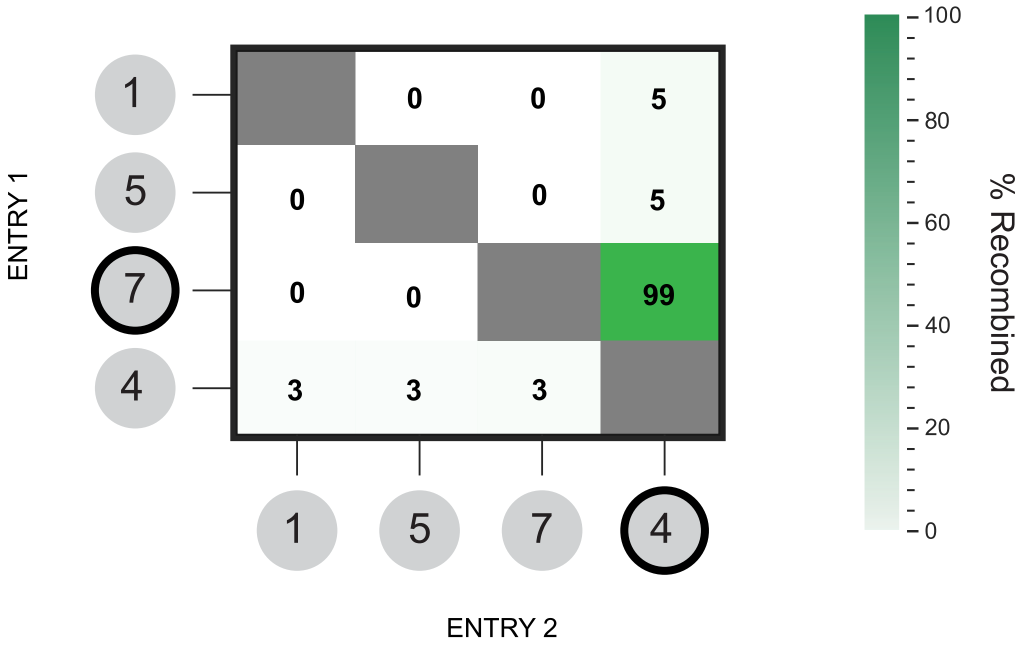

Fig. S14 | $_4P_2$ red team penetration testing results. The blue team selected the 7,4 authentication code permutation lock given in **Fig. 3** to conduct a $_4P_2$ trial run of the biohackathon. The search space consisted of 4 objects (*i.e.*, objects #1, #5, #7, #4). The red team was restricted to 14 days to complete the trial exercise. The red team's results are shown above, with the percentage of the decrypted population for each permutation represented on a color scale, and the actual percentage noted in the center of the box. The red team successfully followed the provided protocol and identified the correct authentication code. Source data are provided, **table S2**. Data represent the average of n = 3 biological replicates.

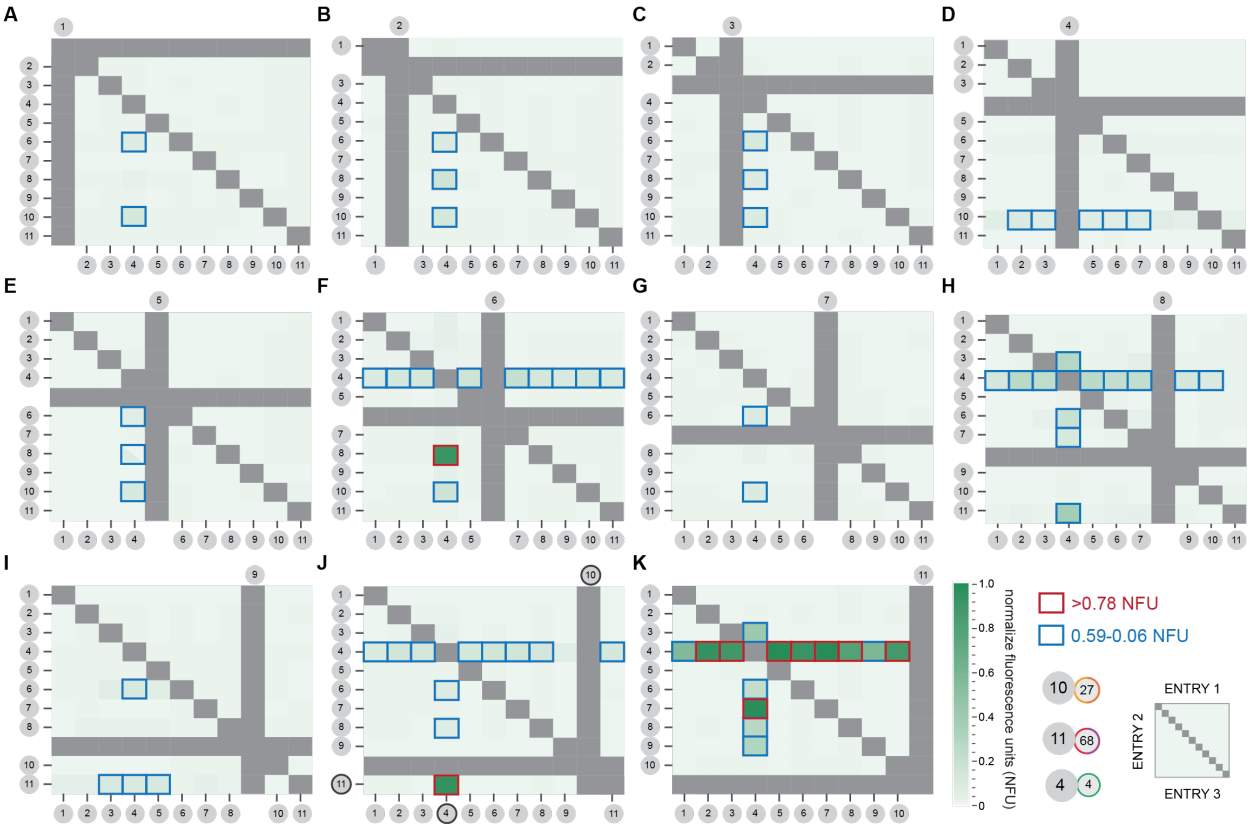

Fig. S15 | Biohackathon: ***11P3*** **red team penetration testing - blue team analysis of iteration 1.** The most notable decryption events occurred in the matrix initiated by object #11 (*i.e.*, decoded as #68) which corresponds to the second entry in the permutation string (**K**, **Fig. 8O**). In particular, substantial decryption occurred when said entry was followed by the introduction of object #4 (*i.e.*, decoded as #4) which corresponds to the third entry in the permutation string. This 2-entry permutation resulted in >0.78 NFU in 7 out of the 9 samples. The remaining 2 samples in this cohort exhibited moderate events at levels between 0.59-0.56 NFU. In the same matrix (*i.e.*, **K**, **Fig. 8O**), the only other samples that resulted in notable (*i.e.*, major or moderate) events occurred when the third entry was object #4 (*i.e.*, decoded as #4). Here 1 out of the 9 samples displayed a major event, whereas 4 out of 9 samples exhibited moderate events (*i.e.*, at levels between 0.22-0.41 NFU). The results from the analysis of the matrix given in **K**, **Fig. 8O** indicate that the cell stock likely experienced an unpremeditated inversion of the set of attachment sites cognate to the first entry object #10 (see **inset**). Said perturbation to the system reduced the permutation string from a 3-entry system to a 2-entry system. Given the heterogeneity in the observed GFP fluorescence intensity the blue team presumed the initial (unintended) recombination event was not uniform throughout the cell culture. In support of this supposition (*i.e.*, an unpremeditated inversion of the set of attachment sites cognate to the first entry object #10 – decoded as #27) the matrix given in (**F**, **Fig. 8J)** also experienced a major event corresponding to the sample transiently exposed to the premutation string 6,8,4 (decoded as 6,8,4). The caveat in this case being a history related decryption event *via* 6,8 which mimics #68 (encoded as #11), which corresponds to the second entry in the permutation string. In addition, a minor event occurred *via* 6,4 in the same matrix implying a minor induction event cognate to object #68. Likewise, an analogous minor induction occurred *via* the complementary single digit (*i.e.*, 8,4 and 8, - ,4) was observed in the matrix given in **H**, **Fig. 8L**. In support of the aforesaid supposition (regarding minor events related to object #68) the matrix given in **D**, **Fig. 8H** also exhibited minor decryption events (0.09-0.07 NFU) potentially cognate to

68 – though out of sequence, implying an incomplete deletion event cognate to object 4 as the first entry and related ligand history. However, given that is the only event where the permutation is incorrect, and all events are minor the blue team regarded this result as inconclusive. The matrix given in **J**, **Fig. 8N** contains the correct authentication code (*i.e.*, 10,11,4). The sample cognate to this entry was categorized as a major decryption event. This result implies that at least some fraction of the population potentially contained the fully encrypted circuit. In addition, in the same matrix permutations (i) 10, 4, - ; (ii) 10, 6, 4; and (iii) 10, 8, 4 resulted in moderate to minor decryption events (*i.e.*, 0.15 – 0.07 NFU) – likely due to minor leaky production of the recombinase cognate to object #68 (encoded as #11). Notably, authentication codes 1,6,4 and 1,6,8 (**A**, **Fig. 8E**); 7,6,4 and 7,8,4 (**G**, **Fig. 8K**); 9,6,4 and 9,6,8 (**I**, **Fig. 8M**) imply that the system can be induced with object #6 (cognate to #68); however, object #8 (also cognate to #68) is insufficient to induce the system alone. This suggests that component #8 of object #68 is primarily responsible for the leaky expression of the cognate recombinase.

Conclusions: From the analysis of the data generated by the red team, the blue team concluded that 3 factors impacted the *11P3* security system and contributed to imperfect authentication and performance:

(i) The first factor was identified as an unpremeditated inversion of the set of attachment sites cognate to the first entry (*i.e.*, object #27 - encoded as entry #10). Said perturbation to the system reduced the permutation string from a 3-entry system to a 2-entry system *via* eliminating the requirement for the first entry. This event likely occurred during the material transfer or during the initial sample prep conducted by the red team. Note, sample contamination cannot be ruled out.

(ii) The second factor was population heterogeneity in the initiation grow culture, resulting in variable levels of protein expression observed in putatively decrypted systems. Sample heterogeneity likely occurred during the sample prep by the red team – though sample contamination at the point of origin cannot be ruled out.

(iii) The third factor was leaky recombinase expression cognate to object #68 (encoded as object #11). Notably, the blue team posited that component #8 of object #68 is primarily responsible for the leaky expression of the cognate recombinase – given that in several cases the system can be induced with object #6 exclusively.

In general, the final circuit was highly composable from the simpler parts and operations developed in this study. The blue team posited that the first two factors could be resolved by adjustments to the sample preparation and improvements to the protocol. Whereas the third factor would require minor circuit tuning. Overall, the blue team posited that addressing factors one and two would have the greatest impact on improving the performance of the security system.

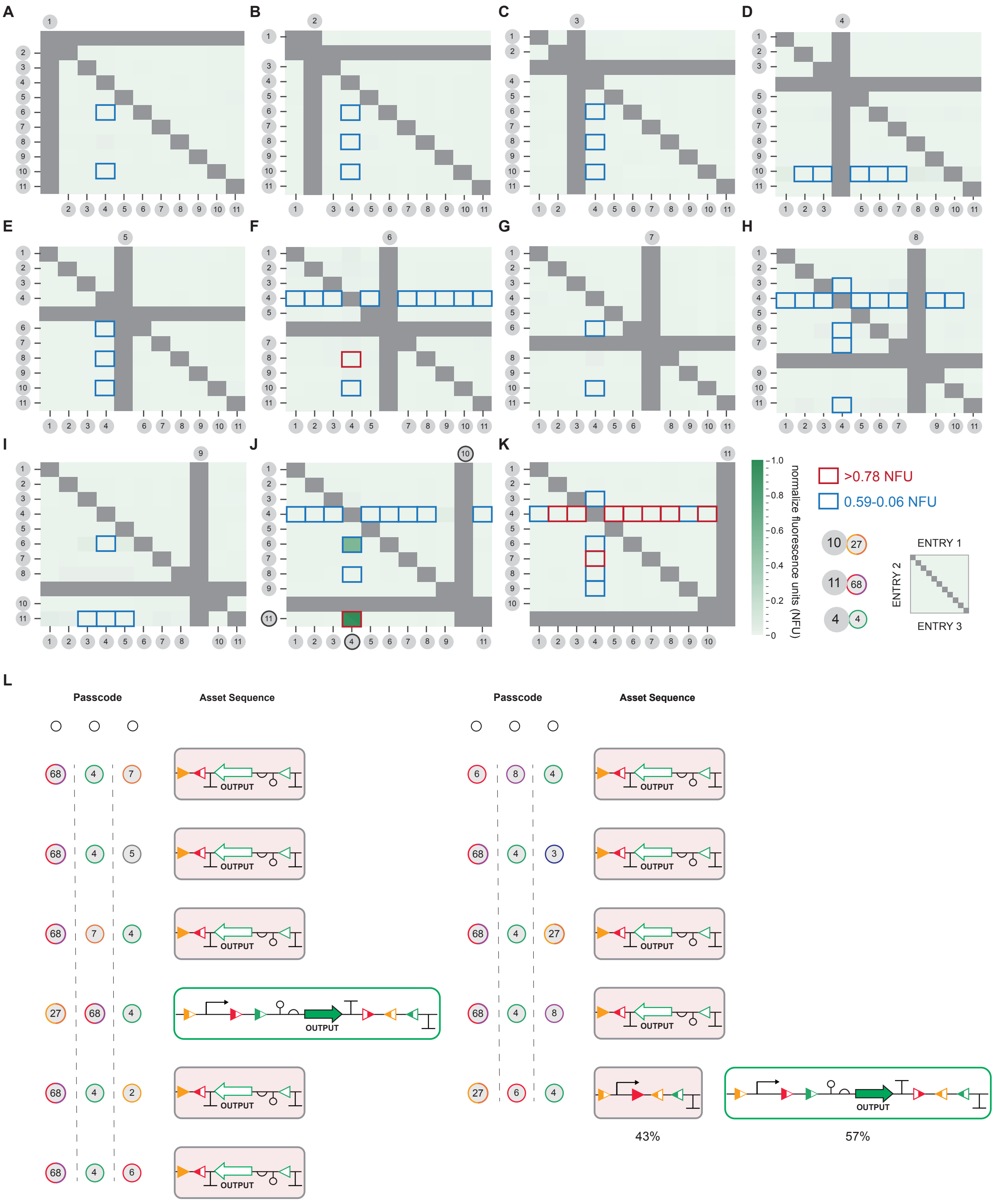

Fig. S16 | Biohackathon: iteration 2. In the second iteration of the *11P3* security exercise in which the red team used a revised protocol to evaluate: (i) the 10 authentication codes that resulted in major events, (ii) the 40 authentication codes that resulted in moderate events, (iii) the 20 authentication codes that resulted in minor events, and (iv) 22 authentication codes that did not decrypt the asset as controls (see **A-K**). The

red team evaluated the 70 authentication codes plus 22 randomly selected controls *via* the testing strain. Of the 92 authentication codes 91 performed as expected based on the design of the security system. Notably, permutation 10,6,4 resulted in a partial decryption of the asset (*i.e.*, 0.65 NFU) – in contrast to full decryption of the asset *via* the correct authentication code 10,11,4 (*i.e.*, 1.0 normalized units) and no decryption of the asset *via* the control authentication codes (*i.e.*, averaged at 0.02 normalized units). This observation affirmed the blue team's supposition regarding factor three – *i.e.*, leaky recombinase expression cognate to object #68 (encoded as object #11). Moreover, the second iteration provided more granularity (and conclusive results) regarding the basis of the performance issue for object #68 in the context of the final security system. Note, the correct authentication code 10,11,4 is decoded as 27,68,4 – herein we will discuss the security system in the context of the decoded objects to improve clarity as said issue is decomposed.

From the second iteration, authentication code 27,6,4 partially decrypted the asset; however, authentication code 27,8,4 did not decrypt the circuit. In the context of object #68 (*i.e.*, the correct second entry) this observation implies that BUFFER operation #8 is not fully repressing the expression of the cognate recombinase alone. Said observation is consistent with data from the first iteration specifically authentication codes 1,6,4 vs. 1,6,8 (**A**); 7,6,4 vs. 7,8,4 (**G**); 9,6,4 vs. 9,6,8 (**I**).

(L) Iteration 2 sequence validation of the 10 authentication codes that resulted in major events from iteration 1, in addition to authentication code 27,6,4. Note, the phenotypes of all samples were validated *via* FACS. For the sequence validation 2 CFUs for each of the 11 authentication codes were sequenced. The correct authentication code 27,68,4 yielded ~100% decryption of the asset, whereas the other 9 authentication codes from this cohort yielded ~0% decryption of the asset. Authentication code 27,6,4 partially decrypted the asset at a 57% ON and 43% OFF population. FACS data correlated with sequencing data. Source data are provided, **table S2**.

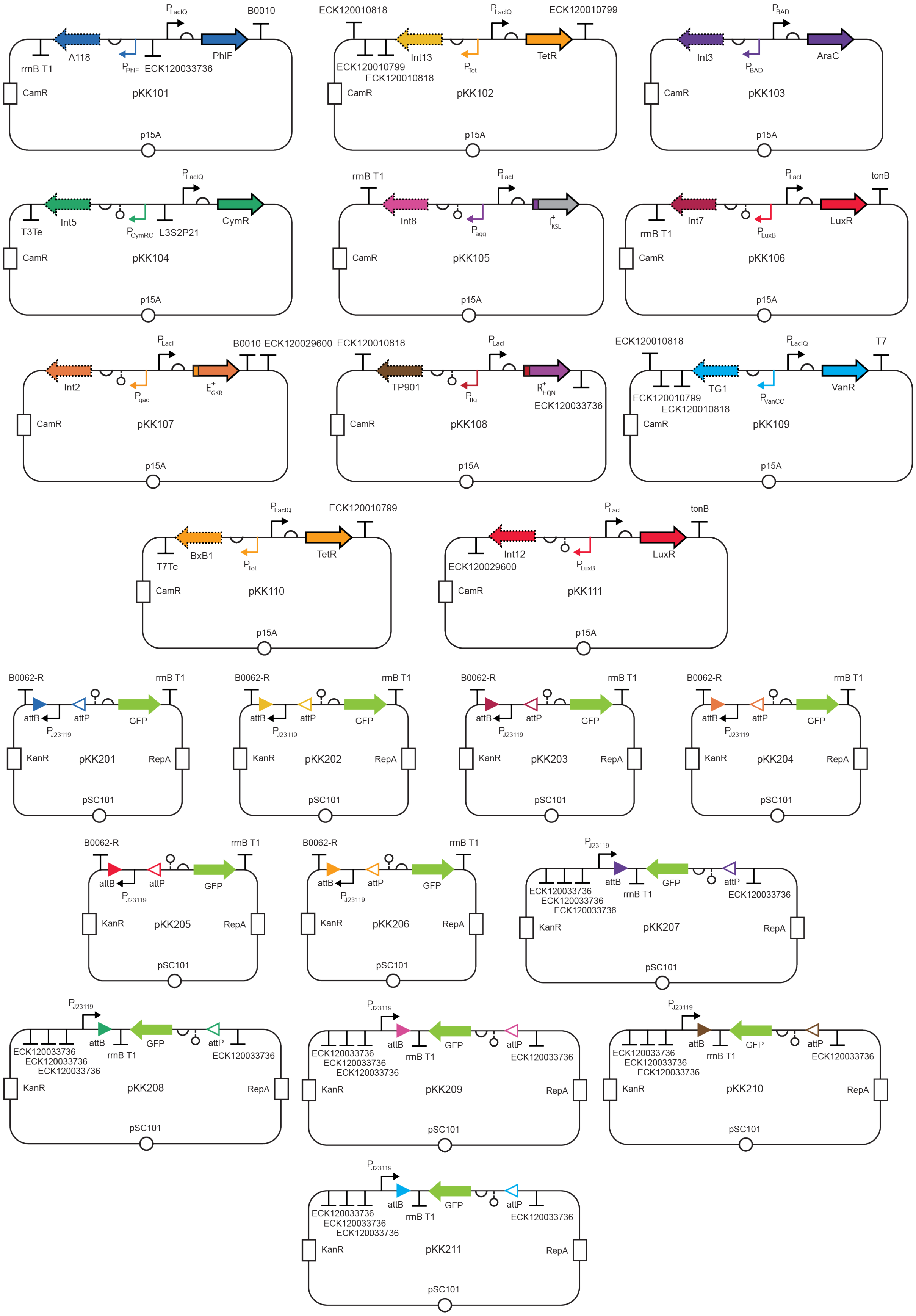

PLacIQ
B0010
A118
PhlF
rrnB T1
PPhlF
ECK120033736
CamR
pKK101
p15A
ECK120010818
PLacIQ
ECK120010799
Int13
TetR
ECK120010799
ECK120010818
PTet
CamR
pKK102
p15A
PBAD
Int3
AraC
PBAD
CamR
pKK103
p15A
PLacIQ
Int5
CymR
T3Te
PCymRC
L3S2P21
CamR
pKK104
p15A
rrnB T1
PLacI
Int8
I+KSL
Pagg
CamR
pKK105
p15A
PLacI
tonB
Int7
LuxR
rrnB T1
PLuxB
CamR
pKK106
p15A
PLacI
B0010 ECK120029600
Int2
Pgac
E+GKR
CamR
pKK107
p15A
ECK120010818
PLacI
TP901
R+HQN
Pttg
ECK120033736
CamR
pKK108
p15A
ECK120010818
PLacIQ
T7
TG1
VanR
ECK120010799
ECK120010818
PVanCC
CamR
pKK109
p15A
PLacIQ
ECK120010799
BxB1
TetR
T7Te
PTet
CamR
pKK110
p15A
PLacI
tonB
Int12
LuxR
ECK120029600
PLuxB
CamR
pKK111
p15A
B0062-R
rrnB T1
attB
attP
GFP
PJ23119
KanR
pKK201
RepA
pSC101
B0062-R
rrnB T1
attB
attP
GFP
PJ23119
KanR
pKK202
RepA
pSC101
B0062-R
rrnB T1
attB
attP
GFP
PJ23119
KanR
pKK203
RepA
pSC101
B0062-R
rrnB T1
attB
attP
GFP
PJ23119
KanR
pKK204
RepA
pSC101
B0062-R
rrnB T1
attB
attP
GFP
PJ23119
KanR
pKK205
RepA
pSC101
B0062-R
rrnB T1
attB
attP
GFP
PJ23119
KanR
pKK206
RepA
pSC101
PJ23119
attB
GFP
attP
ECK120033736
ECK120033736
ECK120033736
rrnB T1
ECK120033736
KanR
pKK207
RepA
pSC101
PJ23119
attB
GFP
attP
ECK120033736
ECK120033736
ECK120033736
rrnB T1
ECK120033736
KanR
pKK208
RepA
pSC101
PJ23119
attB
GFP
attP
ECK120033736
ECK120033736
ECK120033736
rrnB T1
ECK120033736
KanR
pKK209
RepA
pSC101
PJ23119
attB
GFP
attP
ECK120033736
ECK120033736
ECK120033736
rrnB T1
ECK120033736
KanR
pKK210
RepA
pSC101
PJ23119
attB
GFP
attP
ECK120033736
ECK120033736
ECK120033736
rrnB T1
ECK120033736
KanR
pKK211
RepA
pSC101

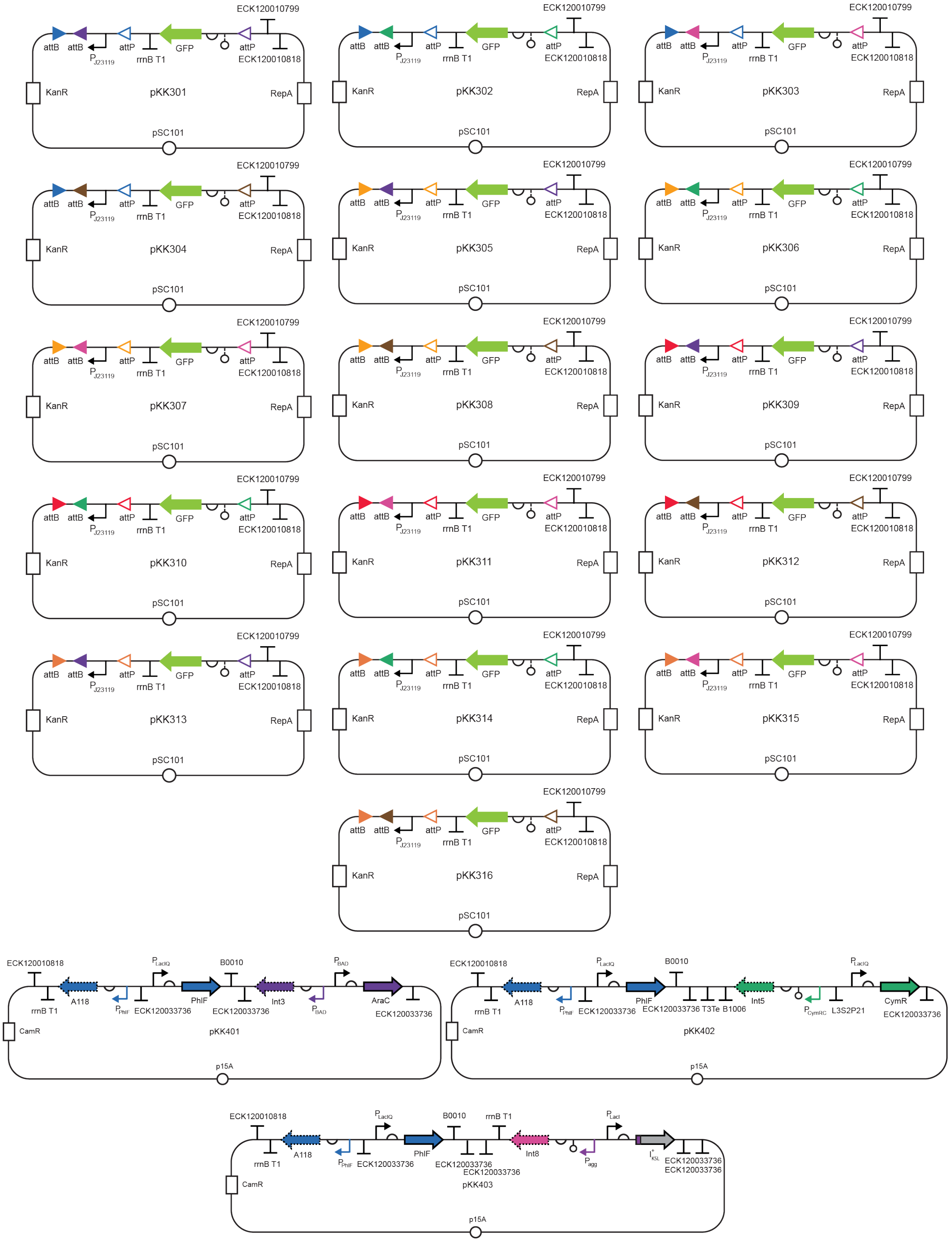

ECK120010799
attB
attB
PJ23119
attP
rrnB T1
GFP
attP
ECK120010818
KanR
pKK301
RepA
pSC101
ECK120010799
attB
attB
PJ23119
attP
rrnB T1
GFP
attP
ECK120010818
KanR
pKK302
RepA
pSC101
ECK120010799
attB
attB
PJ23119
attP
rrnB T1
GFP
attP
ECK120010818
KanR
pKK303
RepA
pSC101
ECK120010799
attB
attB
PJ23119
attP
rrnB T1
GFP
attP
ECK120010818
KanR
pKK304
RepA
pSC101
ECK120010799
attB
attB
PJ23119
attP
rrnB T1
GFP
attP
ECK120010818
KanR
pKK305
RepA
pSC101
ECK120010799
attB
attB
PJ23119
attP
rrnB T1
GFP
attP
ECK120010818
KanR
pKK306
RepA
pSC101
ECK120010799
attB
attB
PJ23119
attP
rrnB T1
GFP
attP
ECK120010818
KanR
pKK307
RepA
pSC101
ECK120010799
attB
attB
PJ23119
attP
rrnB T1
GFP
attP
ECK120010818
KanR
pKK308
RepA
pSC101
ECK120010799
attB
attB
PJ23119
attP
rrnB T1
GFP
attP
ECK120010818
KanR
pKK309
RepA
pSC101
ECK120010799
attB
attB
PJ23119
attP
rrnB T1
GFP
attP
ECK120010818
KanR
pKK310
RepA
pSC101
ECK120010799
attB
attB
PJ23119
attP
rrnB T1
GFP
attP
ECK120010818
KanR
pKK311
RepA
pSC101
ECK120010799
attB
attB
PJ23119
attP
rrnB T1
GFP
attP
ECK120010818
KanR
pKK312
RepA
pSC101
ECK120010799
attB
attB
PJ23119
attP
rrnB T1
GFP
attP
ECK120010818
KanR
pKK313
RepA
pSC101
ECK120010799
attB
attB
PJ23119
attP
rrnB T1
GFP
attP
ECK120010818
KanR
pKK314
RepA
pSC101
ECK120010799
attB
attB
PJ23119
attP
rrnB T1
GFP
attP
ECK120010818
KanR
pKK315
RepA
pSC101
ECK120010799
attB
attB
PJ23119
attP
rrnB T1
GFP
attP
ECK120010818
KanR
pKK316
RepA
pSC101
ECK120010818
rrnB T1
A118
PPhlF
ECK120033736
PLacIQ
PhlF
B0010
ECK120033736
Int3
PBAD
PBAD
AraC
ECK120033736
CamR
pKK401
p15A
ECK120010818
rrnB T1
A118
PPhlF
ECK120033736
PLacIQ
PhlF
B0010
ECK120033736 T3Te B1006
Int5
PCymRC
L3S2P21
PLacIQ
CymR
ECK120033736
CamR
pKK402
p15A
ECK120010818
rrnB T1
A118
PPhlF
ECK120033736
PLacIQ
PhlF
B0010
ECK120033736
ECK120033736
rrnB T1
Int8
Pagg
PLacI
ECK120033736
ECK120033736
CamR
pKK403
p15A

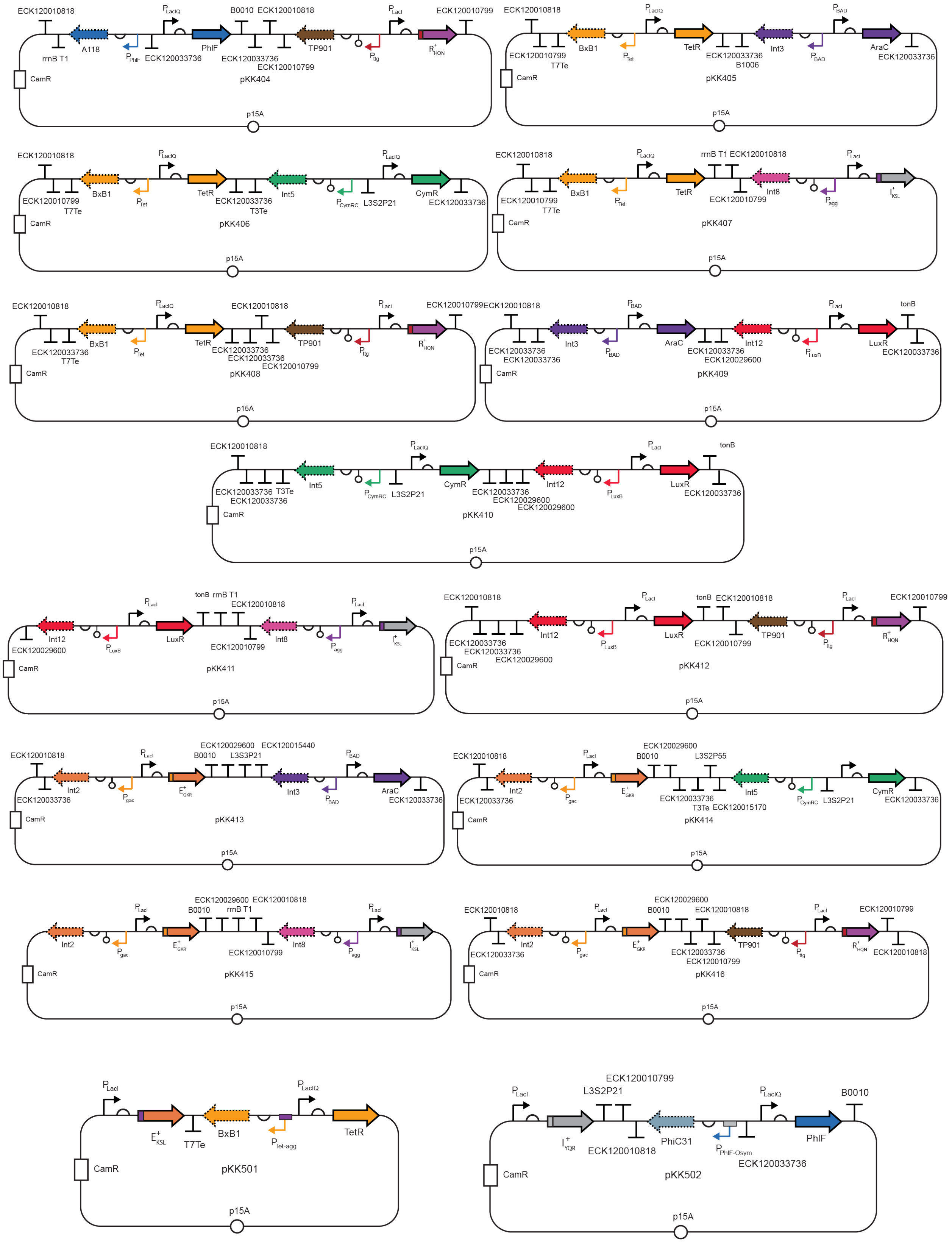

ECK120010818
PLacIQ
B0010 ECK120010818
PLacI
ECK120010799
rrnB T1
A118
PPhlF
ECK120033736
PhlF
ECK120033736
ECK120010799
TP901
Pttg
R+HQN
CamR
pKK404
p15A
ECK120010818
PLacIQ
PBAD
ECK120010799
T7Te
BxB1
PTet
TetR
ECK120033736
B1006
Int3
PBAD
AraC
ECK120033736
CamR
pKK405
p15A
ECK120010818
PLacIQ
PLacIQ
ECK120010799
T7Te
BxB1
PTet
TetR
ECK120033736
T3Te
Int5
PCymRC
L3S2P21
CymR
ECK120033736
CamR
pKK406
p15A
ECK120010818
PLacIQ
rrnB T1 ECK120010818
PLacI
ECK120010799
T7Te
BxB1
PTet
TetR
ECK120010799
Int8
Pagg
I+KSL
CamR
pKK407
p15A
ECK120010818
PLacIQ
ECK120010818
PLacI
ECK120010799ECK120010818
ECK120033736
T7Te
BxB1
Ptet
TetR
ECK120033736
ECK120033736
ECK120010799
TP901
Pttg
R+HQN
CamR
pKK408
p15A
PBAD
PLacI
tonB
ECK120033736
ECK120033736
Int3
PBAD
AraC
ECK120033736
ECK120029600
Int12
PLuxB
LuxR
ECK120033736
CamR
pKK409
p15A
ECK120010818
PLacIQ
PLacI
tonB
ECK120033736 T3Te
ECK120033736
Int5
PCymRC
L3S2P21
CymR
ECK120033736
ECK120029600
ECK120029600
Int12
PLuxB
LuxR
ECK120033736
CamR
pKK410
p15A
PLacI
tonB rrnB T1
ECK120010818
PLacI
Int12
ECK120029600
PLuxB
LuxR
ECK120010799
Int8
Pagg
I+KSL
CamR
pKK411
p15A
ECK120010818
PLacI
tonB ECK120010818
PLacI
ECK120010799
ECK120033736
ECK120033736
ECK120029600
Int12
PLuxB
LuxR
ECK120010799
TP901
Pttg
R+HQN
CamR
pKK412
p15A
ECK120010818
PLacI
ECK120029600 ECK120015440
B0010 L3S3P21
PBAD
ECK120033736
Int2
Pgac
E+GKR
Int3
PBAD
AraC
ECK120033736
CamR
pKK413
p15A
ECK120010818
PLacI
ECK120029600
B0010
L3S2P55
ECK120033736
Int2
Pgac
E+GKR
ECK120033736
T3Te ECK120015170
Int5
PCymRC
L3S2P21
CymR
ECK120033736
CamR
pKK414
p15A
PLacI
ECK120029600 ECK120010818
B0010
rrnB T1
PLacI
Int2
Pgac
E+GKR
ECK120010799
Int8
Pagg
I+KSL
CamR
pKK415
p15A
ECK120010818
PLacI
ECK120029600
B0010
ECK120010818
PLacI
ECK120010799
ECK120033736
Int2
Pgac
E+GKR
ECK120033736
ECK120010799
TP901
Pttg
R+HQN
ECK120010818
CamR
pKK416
p15A
PLacI
PLacIQ
E+KSL
T7Te
BxB1
PTet-agg
TetR
CamR
pKK501
p15A
ECK120010799
PLacI
L3S2P21
PLacIQ
B0010
I+YQR
ECK120010818
PhiC31
PPhlF-Osym
ECK120033736
PhlF
CamR
pKK502
p15A

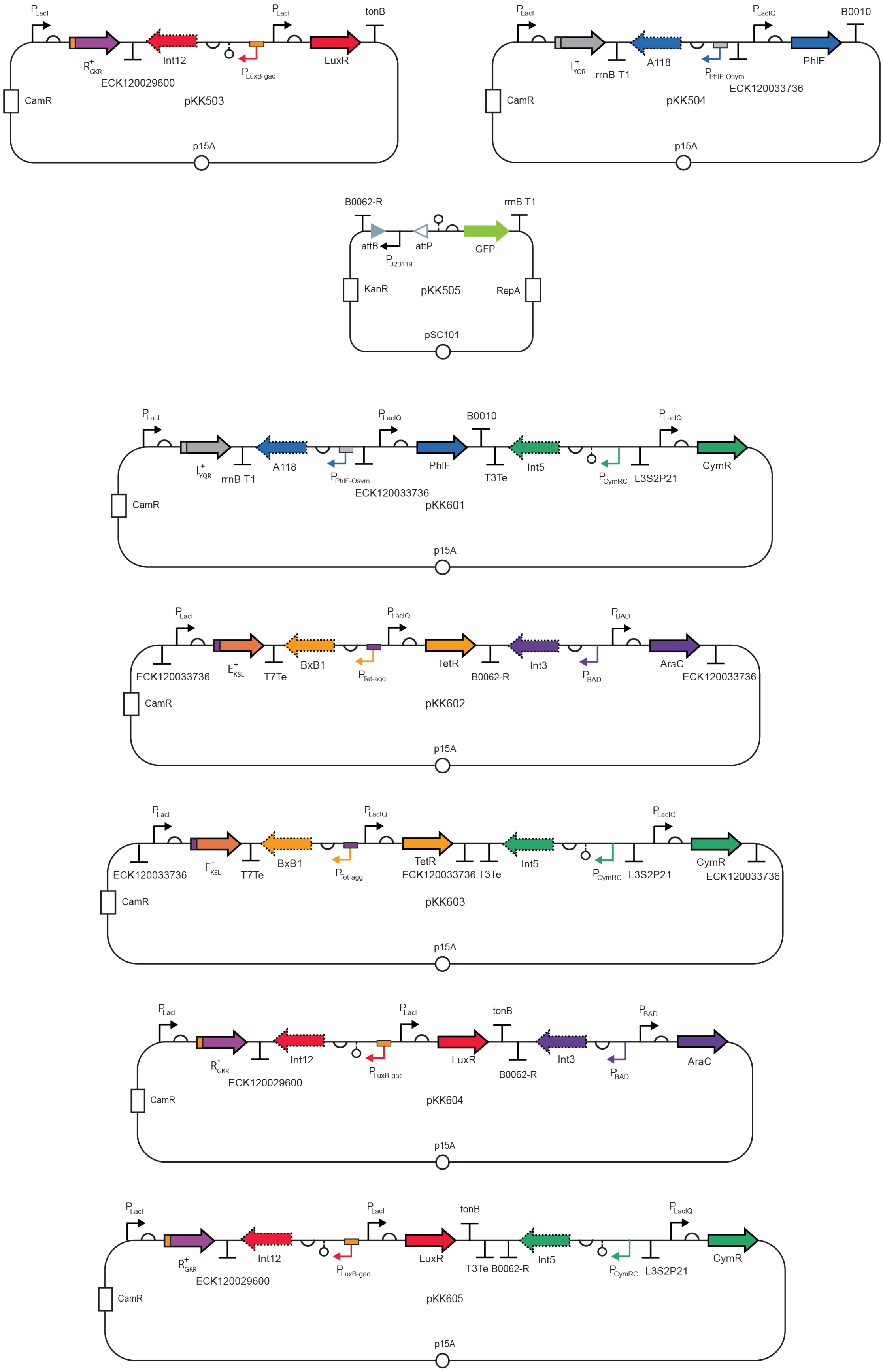
P_{LacI}
P_{LacI}
tonB
R^{+}_{GKR}
Int12
LuxR
ECK120029600
$P_{LuxB\text{-}gac}$
CamR
pKK503
p15A
P_{LacI}
P_{LacIQ}
B0010
I^{+}_{YQR}
rrnB T1
A118
PhlF
$P_{PhlF\text{-}Osym}$
ECK120033736
CamR
pKK504
p15A
B0062-R
rrnB T1
attB
attP
GFP
P_{J23119}
KanR
pKK505
RepA
pSC101
P_{LacI}
P_{LacIQ}
B0010
P_{LacIQ}
I^{+}_{YQR}
rrnB T1
A118
PhlF
Int5
CymR
T3Te
$P_{PhlF\text{-}Osym}$
P_{CymRC}
L3S2P21
ECK120033736
CamR
pKK601
p15A
P_{LacI}
P_{LacIQ}
P_{BAD}
BxB1
TetR
Int3
AraC
ECK120033736
E^{+}_{KSL}
T7Te
$P_{Tet\text{-}agg}$
B0062-R
P_{BAD}
ECK120033736
CamR
pKK602
p15A
P_{LacI}
P_{LacIQ}
P_{LacIQ}
E^{+}_{KSL}
BxB1
TetR
Int5
CymR
ECK120033736
T7Te
$P_{Tet\text{-}agg}$
ECK120033736 T3Te
P_{CymRC}
L3S2P21
ECK120033736
CamR
pKK603
p15A
P_{LacI}
P_{LacI}
tonB
P_{BAD}
R^{+}_{GKR}
Int12
LuxR
Int3
AraC
ECK120029600
$P_{LuxB\text{-}gac}$
B0062-R
P_{BAD}
CamR
pKK604
p15A
P_{LacI}
P_{LacI}
tonB
P_{LacIQ}
R^{+}_{GKR}
Int12
LuxR
Int5
CymR
ECK120029600
$P_{LuxB\text{-}gac}$
T3Te B0062-R
P_{CymRC}
L3S2P21
CamR
pKK605
p15A

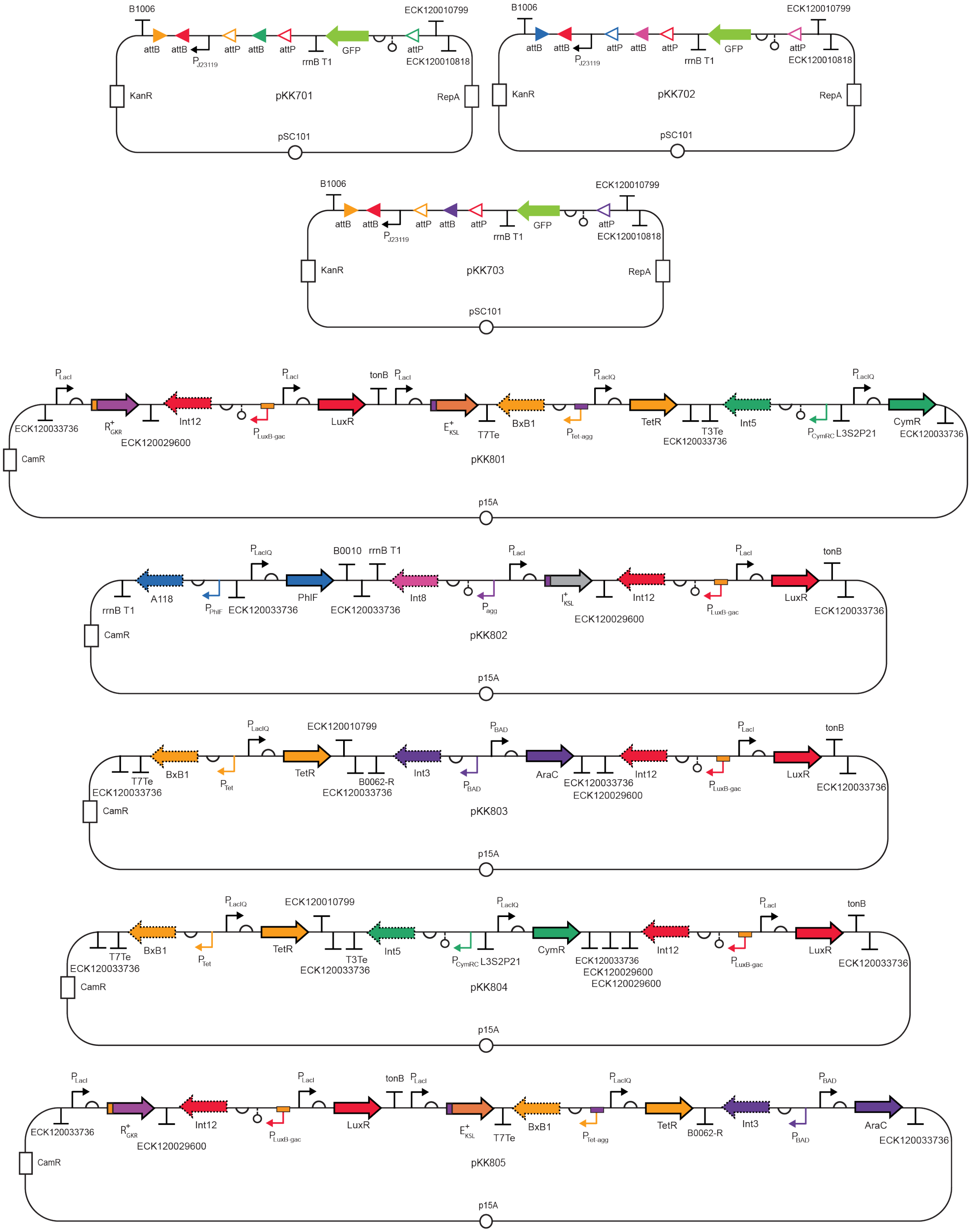

B1006
attB
attB
P_{J23119}
attP
attB
attP
rrnB T1
GFP
ECK120010799
attP
ECK120010818
KanR
pKK701
RepA
pSC101
B1006
attB
attB
P_{J23119}
attP
attB
attP
rrnB T1
GFP
ECK120010799
attP
ECK120010818
KanR
pKK702
RepA
pSC101
B1006
attB
attB
P_{J23119}
attP
attB
attP
rrnB T1
GFP
ECK120010799
attP
ECK120010818
KanR
pKK703
RepA
pSC101
P_{LacI}
ECK120033736
R^{+}_{GKR}
Int12
ECK120029600
$P_{LuxB\text{-}gac}$
P_{LacI}
LuxR
tonB
P_{LacI}
E^{+}_{KSL}
T7Te
BxB1
P_{LacIQ}
$P_{Tet\text{-}agg}$
TetR
T3Te
ECK120033736
Int5
P_{LacIQ}
P_{CymRC}
L3S2P21
CymR
ECK120033736
CamR
pKK801
p15A
rrnB T1
A118
P_{PhlF}
P_{LacIQ}
ECK120033736
PhlF
B0010
rrnB T1
ECK120033736
Int8
P_{agg}
P_{LacI}
I^{+}_{KSL}
ECK120029600
Int12
$P_{LuxB\text{-}gac}$
P_{LacI}
LuxR
tonB
ECK120033736
CamR
pKK802
p15A
T7Te
ECK120033736
BxB1
P_{Tet}
P_{LacIQ}
TetR
ECK120010799
B0062-R
ECK120033736
Int3
P_{BAD}
P_{BAD}
AraC
ECK120033736
ECK120029600
Int12
$P_{LuxB\text{-}gac}$
P_{LacI}
LuxR
tonB
ECK120033736
CamR
pKK803
p15A
T7Te
ECK120033736
BxB1
P_{Tet}
P_{LacIQ}
TetR
ECK120010799
T3Te
ECK120033736
Int5
P_{CymRC}
L3S2P21
P_{LacIQ}
CymR
ECK120033736
ECK120029600
ECK120029600
Int12
$P_{LuxB\text{-}gac}$
P_{LacI}
LuxR
tonB
ECK120033736
CamR
pKK804
p15A
P_{LacI}
ECK120033736
R^{+}_{GKR}
ECK120029600
Int12
$P_{LuxB\text{-}gac}$
P_{LacI}
LuxR
tonB
P_{LacI}
E^{+}_{KSL}
T7Te
BxB1
$P_{Tet\text{-}agg}$
P_{LacIQ}
TetR
B0062-R
Int3
P_{BAD}
P_{BAD}
AraC
ECK120033736
CamR
pKK805
p15A

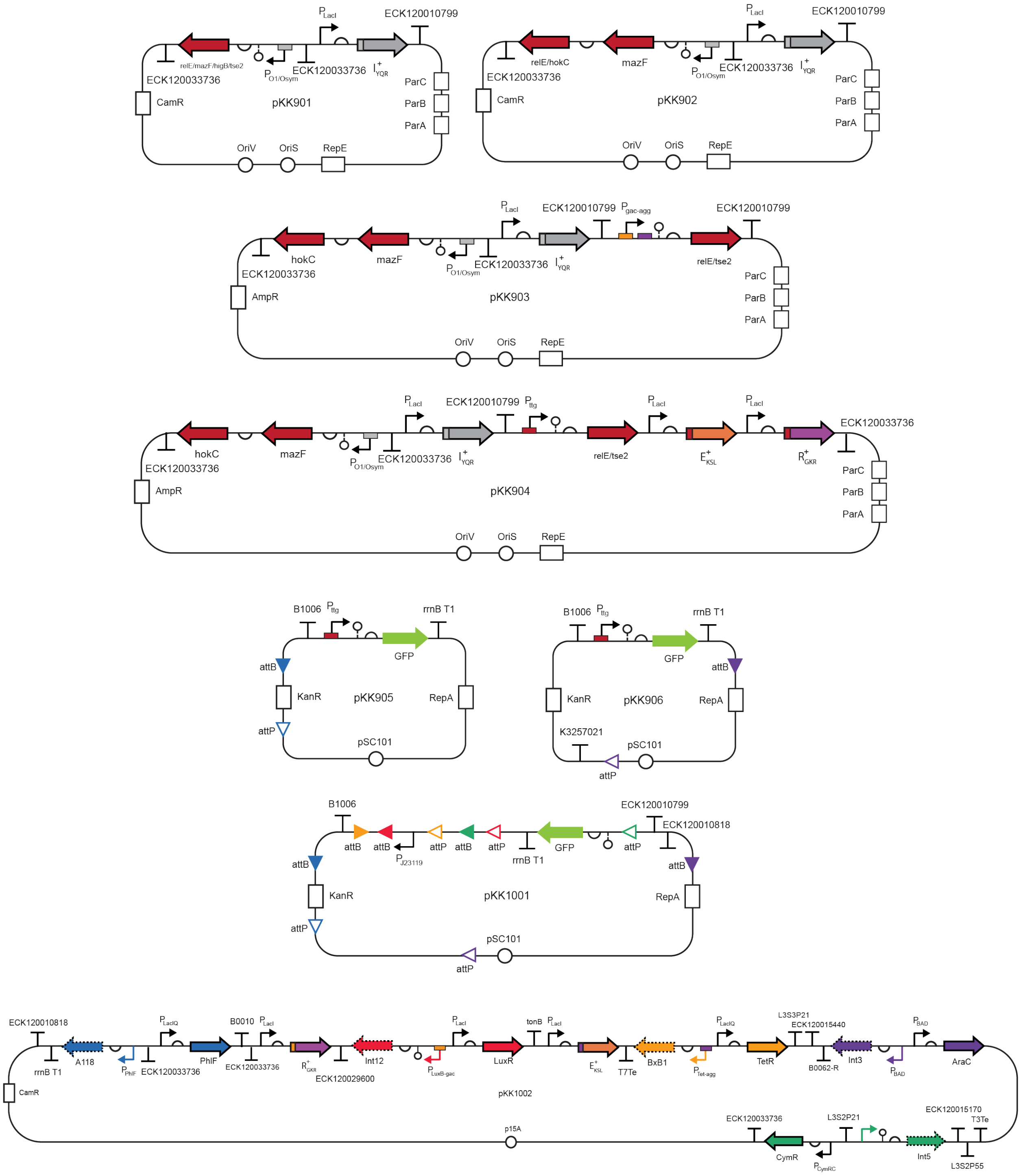

Fig. S17 | Relevant plasmid maps for this study. Sequences can be found as GenBank accession numbers PX738379- PX738464, https://www.ncbi.nlm.nih.gov/genbank/.

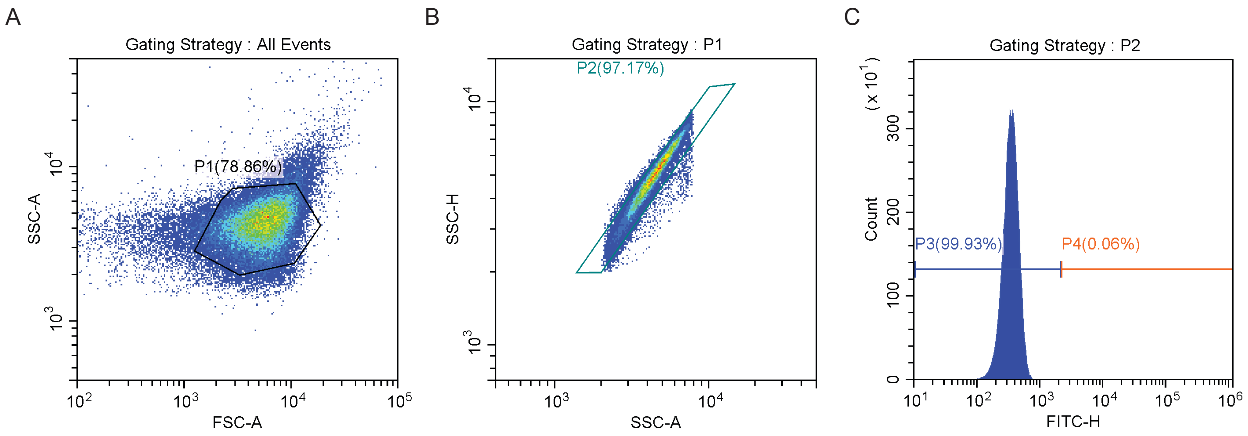

Fig. S18 | Gating strategy for FACS analysis. Representative gating strategy for FACS analysis is shown. **(A)** Cells were first gated by forward scatter area (FSC-A) and side scatter area (SSC-A) (P1). **(B)** Then the population was gated again by side scatter area (SSC-A) and side scatter height (SSC-H) to gate the single cells (P2). **(C)** Lastly, cells are gated by FITC-H value and cell count to measure the cell population based on fluorescence. Cells with FITC-H values lower than $2x10^3$ are regarded as the GFP OFF-state (P3), and those with values higher than $2x10^3$ are regarded as the GFP ON-state (P4).

Supplementary Protocol 1

Biohackathon Protocol: *4P2* Preliminary Security System

Overview. The blue team has engineered a biological security system such that the asset is represented as a simple genetic circuit composed of a constitutive promoter, genetic insulator, RBS, gene of interest (GoI), and terminator. For the purpose of this exercise, a circuit expressing green fluorescent protein (GFP) will serve as the asset. Said asset is protected *via* a permutation lock – *i.e.*, GFP cannot be expressed until the correct authentication code is supplied.

In this preliminary exercise you (the red team) are tasked with identifying the correct authentication code for a security system composed of n objects ($n = 4$) in which you are required to choose r entries ($r = 2$) to unlock the system, such that the order of said entries is distinct – *i.e.*, objectively represented as a nPr permutation lock (**equation S1**).

$$nPr = \frac{n!}{(n-r)!} \qquad \textbf{(S1)}$$

This ethical hacking exercise is structured as a "gray box" challenge in which the red team is provided with partial information regarding the security system to facilitate testing and learning. Namely, the red team will be informed of the number of objects n that compose the search space and information regarding the length of the permutation string r. However, the chemical identities of the objects n (and cognate entries r) and genetic details will not be disclosed by the blue team. In addition, there are constraints on: (i) the number of personnel – *i.e.*, no more than two people, (ii) methodologies used during the ethical hacking exercise – *i.e.*, no part of the system can be sequenced, tampered with, or modified – however, limited automation is acceptable, and (iii) the time to identify the authentication cognate to the *4P2* lock (composed of 12 permutations) is restricted to 14 days. *In other words, the objective is to unlock the asset via the correct authentication code alone, within 14 days, using no more than two people.*

The preliminary exercise is intended to be tractable and is designed to introduce the red team to the general protocol that will be used in the full-scale exercise. Upon completion of this exercise the red team will have an opportunity to discuss any technical, logistical, our communication issues identified while conducting the preliminary exercise during a post-exercise debriefing. Along with this set of instructions you (the red team) will find a material kit containing: (i) the encrypted biological asset, and (ii) 4 cognate object entries, identities not disclosed (see **fig. S19**). Expression of the asset can be quantified using standard spectroscopy techniques (*i.e.*, micro-well plate reader, FACS or the like). *However, the objective is to identify the correct authentication code such that GFP expression is observed in > 80% of the chassis cell population determined by FACS or the like, with statistical significance based on $n \geq 3$.*

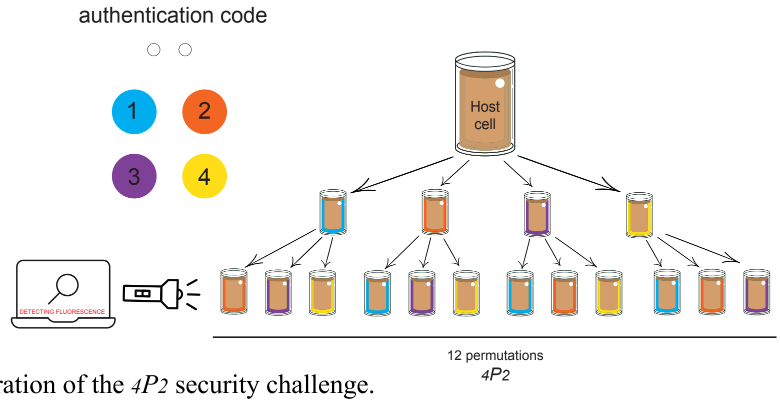

Fig. S19 | Illustration of the *4P2* security challenge.

Material Kit

The material kit (**Fig. S20**) contains:

1. physical host cell colony (agar stab)
2. four objects – *i.e.*, undisclosed chemical entries (concentrated stock solutions)
3. pre-mixed antibiotics (100x concentrated stock)
4. an experimental protocol for the ethical hacking exercise

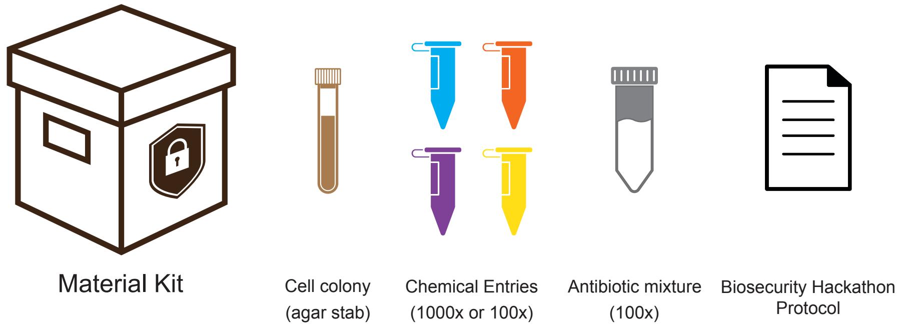

Fig. S20 | Summary of the contents of the 4P2 material kit.

1. NOTE 1: Red team personnel conducting the ethical hacking exercise is restricted to no more than 2 people. The system is designed to be used by a single person; accordingly, the security exercise does not require more than one person.

2. NOTE 2: No part of the system can be sequenced, tampered with, or modified to realize the authentication code.

3. Media Recipes

   a. LB Broth Media

   Use LB Miller Medium and follow product preparation instructions.

   b. M9 Minimal Media

   1) Prepare Stock Solutions (sterile, solvent: DI H_2O)

   5x M9 Salts (Autoclave)
   0.1M $CaCl_2$ (Autoclave)
   0.1M $MgSO_4$ (Autoclave)
   2.5% (w/v) Casamino acid (Autoclave)
   40% (w/v) D-glucose (Sterile filter)
   0.1M thiamine hydrochloride (Sterile filter)
   DI H_2O (Autoclave)

   2) To make 1L of 5x M9 salts,

   Mix:
   15 g/L KH_2PO_4
   2.5 g/L NaCl
   64.0 g/L Na_2HPO_4-$7H_20$
   5 g/L NH_4Cl
   800mL DI H_2O

   > Adjust to 1L DI H_2O and autoclave

3) To make 25mL of 2x M9 minimal media,

Mix:
10mL of 5x M9 Salts
50μL of 0.1M $CaCl_2$
1mL of 0.1M $MgSO_4$
4mL of 2.5% (w/v) Casamino acid
1mL of 40% (w/v) D-glucose
0.5mL of 0.1M thiamine hydrochloride
8.5mL of autoclaved DI H_2O

4) To make the final 10mL of 1x M9 minimal media with object entry,

Mix:
2x M9 minimal media (5mL)
Concentrated entry solution (x mL) (if applicable)
100x Antibiotics Mix (0.1mL)
Autoclaved DI H_2O ((5–x) mL)

<u>c. Phosphate-buffered Saline (PBS Buffer)</u>

To make 1L of PBS buffer,

Mix:
8 g of NaCl
0.2 g KCl
1.44 g Na_2HPO_4
0.245 g KH_2PO_4
800mL DI H_2O

> Adjust to 1L DI H_2O and autoclave.
*Adjust solution to desired pH 7.4 with HCl or NaOH.

4. Metrics used to determine completion of the ethical hacking exercise. Successful completion of the penetration test should satisfy the following criteria: (i) entry of the putatively correct authentication code must result in the expression of GFP in at least 80% of the chassis cell population on average, measured using a flow cytometer with statistical significance ($n \geq 3$). (ii) The red team must provide the exact authentication code that resulted in the expression of the asset. (iii) The *4P2* exercise must be completed within 14 days.

Decryption testing protocol:

Refer to the instructions below for the assay protocol to test each authentication code. All steps should be conducted under sterile conditions. A visual representation is also provided (see **fig. S21**).

1. Take the agar stabbed colonies and pre-culture them into LB Broth Media supplemented with antibiotics (for a minimum time of 1 hour, at 37ºC, while shaking at 750 RPM) and then streak the cells on an agar plate supplemented with antibiotics. Incubate the plate overnight at 37ºC.
2. Pick a colony from the agar plate and start a pre-culture in 200 uL LB Broth media supplemented with antibiotics, in a 96-well plate. Repeat this for desired number of biological replicates Seal the plate with a Breath Easier membrane. Culture them for 8 hours at 37°C, while shaking at 300 RPM.
3. Entry 1.1: After 8 hours, the cells should be diluted at a 1:200 ratio into M9 minimal media with the first entry (1uL of cell culture + 199uL of M9 minimal media with the selected entry).

This should be in a 96-well plate and sealed with a Breath Easier membrane. Grow the cells for 12 hours at 37°C, while shaking at 300 rpm.

4. Entry 1.2: After 12 hours of growth, dilute the cell cultures at a 1:200 ratio into fresh M9 Minimal Media with the same entry used in step 3 (1uL of cell culture + 199uL of M9 minimal media with the selected entry). Seal the 96-well plate with a Breath Easier membrane and grow the cells for an additional 12 hours. NOTE: Cells should be diluted in fresh minimal media at a 1:200 ratio every 12 hours for ideal cellular growth – each entry exposure should be 12 hours x 2 = 24 hours (*i.e.*, object exposure twice with the same entry) for best performance.
5. Entry 2.1: For the second entry, dilute the grown cells from step 4 at a 1:200 ratio into fresh M9 Minimal Media with the second entry (1uL of cell culture + 199uL of M9 minimal media with the selected entry). Seal the 96-well plate with a Breath Easier membrane and grow the cells for an additional 12 hours. NOTE: Authentication codes do NOT use the same object entry more than once.
6. Entry 2.2: After 12 hours of growth, dilute the cell cultures at a 1:200 ratio into fresh M9 minimal media with the same entry used in step 5 (1uL of cell culture + 199uL of M9 minimal media with the selected entry). Seal the 96-well plate with a Breath Easier membrane and grow the cells for an additional 12 hours.
7. After the entry exposure steps are completed, dilute the cells from step 6 into fresh M9 minimal media without entry, at a 1:200 ratio (1uL of cell culture + 199uL of M9 minimal media without entry). Seal the 96-well plate with a Breath Easier membrane and grow the cells for an additional 12 hours.
8. After 12 hours, prepare the cells for FACS (see FACS Protocol).

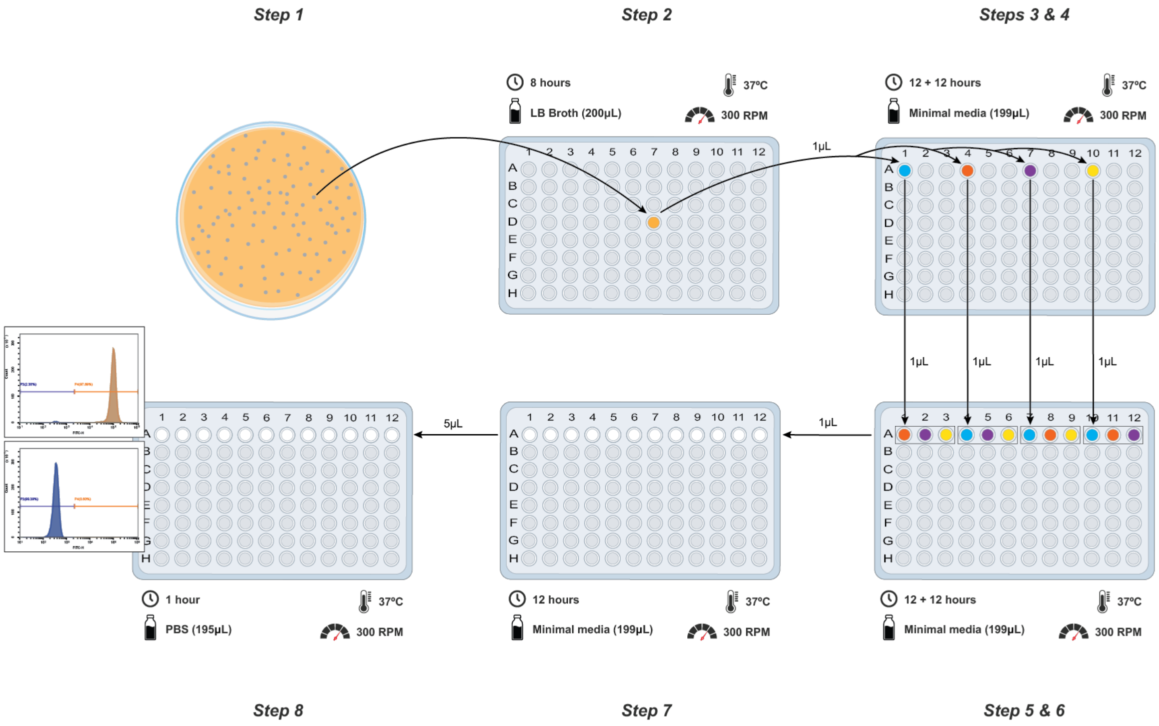

Fig. S21 | Illustration of the assay for *4P2* decryption testing.

FACS protocol:
FACS is required to quantify the population of cells that are expressing GFP. The instructions for preparing your cells for FACS are as follows (also see **fig. S22**):

1. After the 12-hour outgrowth in M9 minimal media without entries, dilute the cells at a 1:40 ratio into PBS with 2 mg/ml kanamycin in a 96-well plate (5uL of cell culture + 195uL of PBS) under sterile conditions. Next, incubate the plate for at least 1 hour at room temperature.
2. Set the flow rate for the flow cytometer between 10-30 ul/min. Next, the cells should be monitored through the FITC channel for GFP expression. Events should be gated by forward scatter area versus side scatter area to eliminate debris, and then gated by side scatter height versus side scatter area to discriminate doublets.
3. More than 50,000 events must be collected for the final analysis.

Biohackathon Penetration Test Timeline (2-ENTRY passcode)

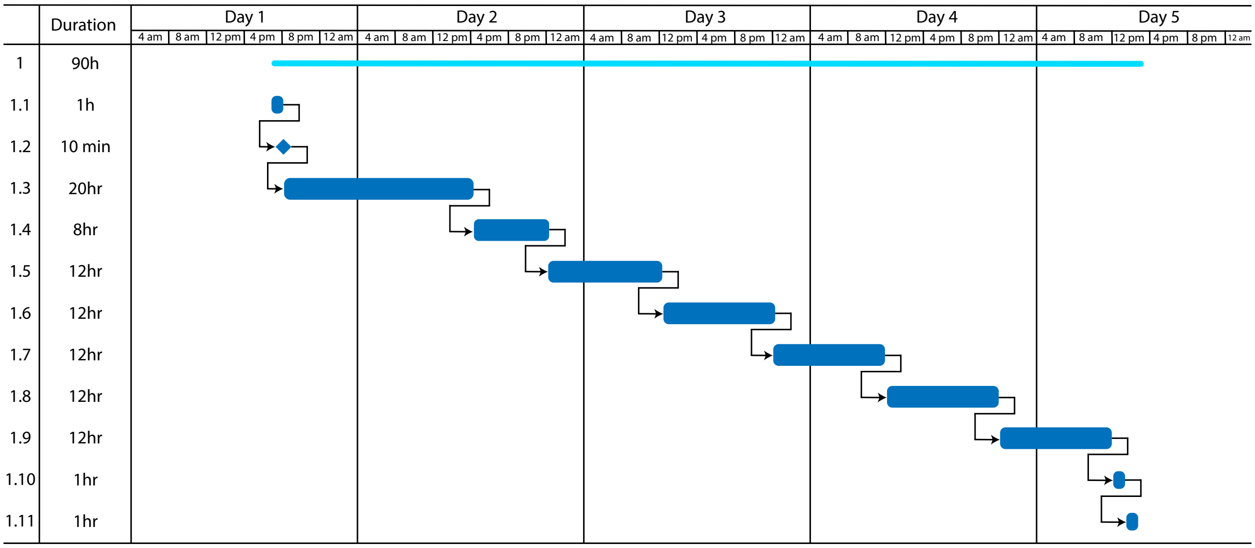

Legend

1	Biohackathon Penetration Test Timeline (Total)
1.1	Pre-culture agar-stabbed colony in LB broth
1.2	Streak pre-culture on agar plate
1.3	Incubate the plate overnight
1.4	Pre-culture individual colonies
1.5	Passage cells to minimal media with first digit inducers (ENTRY 1.1)
1.6	Passage cells to minimal media with first digit inducers (ENTRY 1.2)
1.7	Passage cells to minimal media with second digit inducers (ENTRY 2.1)
1.8	Passage cells to minimal media with second digit inducers (ENTRY 2.2)
1.9	Passage cells to minimal media with no inducers (Outgrowth)
1.10	Dilute cells into PBS for flow cytometry
1.11	Check fluorescence expression of cells with a flow cytometer

Fig. S22 | Instructions and illustration for preparing the *4P2* preliminary security system

Supplementary Protocol 2

Biohackathon Protocol: *11P3* Security System (iteration 1)

Overview. The blue team has engineered a biological security system such that the asset is represented as a simple genetic circuit composed of a constitutive promoter, genetic insulator, RBS, gene of interest (GoI), and terminator. For the purpose of this exercise, a circuit expressing green fluorescent protein (GFP) will serve as the asset. Said asset is protected *via* a permutation lock – *i.e.*, GFP cannot be expressed until the correct authentication code is supplied.

In this exercise you (the red team) are tasked with identifying the correct authentication code for a security system composed of n objects (n = 11) in which you are required to choose r entries (r = 3) to unlock the system, such that the order of said entries is distinct – *i.e.*, objectively represented as a nPr permutation lock (**equation S1**).

This ethical hacking exercise is structured as a "gray box" challenge in which the red team is provided with partial information regarding the security system to facilitate testing and learning. Namely, the red team will be informed of the number of objects n that compose the search space and information regarding the length of the permutation string r. However, the chemical identities of the objects n (and cognate entries r) and genetic details will not be disclosed by the blue team. In addition, there are constraints on: (i) the number of personnel – *i.e.*, no more than two people, (ii) methodologies used during the ethical hacking exercise – *i.e.*, no part of the system can be sequenced, tampered with, or modified – however, limited automation is acceptable, and (iii) the time to identify the authentication cognate to the *11P3* lock (composed of 990 permutations) is restricted to 30 days. *In other words, the objective is to unlock the asset via the correct authentication code alone, within 30 days, using no more than two people.*

This full-scale exercise is intended to be tractable (but not trivial). The full-scale ethical hacking exercise is composed of two parts – a testing phase and a validation phase. The testing phase will be conducted using a chassis cell that contains the *11P3* encrypted asset without penalties. In turn, the validation phase will be conducted *via* a chassis cell containing the same *11P3* encrypted asset with penalties. The red team should conduct the exercise first using the testing chassis cell, followed by confirmation of the final authentication code(s) using the validation chassis cell.

Along with this set of instructions you (the red team) will find a material kit containing: (i) the encrypted biological asset – testing strain and validation strain, and (ii) 11 cognate object entries, identities not disclosed. Expression of the asset can be quantified using standard spectroscopy techniques (*i.e.*, micro-well plate reader, FACS or the like). *However, the objective is to identify the correct authentication code such that GFP expression is observed in > 80% of the chassis cell population determined by flow cytometry or the like, with statistical significance based on n ≥ 3.*

Material Kit

The material kit (**fig. S23**) contains:

1. physical host cell colony – 1 testing strain and 1 validation strain (agar stab)
2. four objects – *i.e.*, undisclosed chemical entries (concentrated stock solutions)
3. pre-mixed antibiotics (100x concentrated stock)
4. an experimental protocol for the ethical hacking exercise

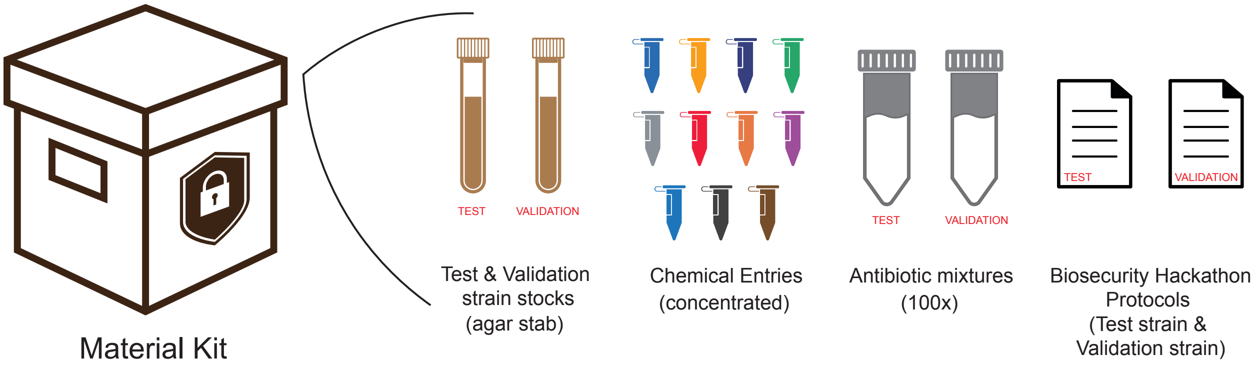

Fig. S23 | Summary of the contents of the material kit for the *11P3* challenge.

1. NOTE 1: red team personnel conducting the ethical hacking exercise is restricted to no more than two people. The system is designed to be used by a single person; accordingly, the security exercise does not require more than one person.

2. NOTE 2: No part of the system can be sequenced, tampered with, or modified to realize the authentication code.

3. Media Recipes
<u>a. LB Broth Media</u>
Use LB Miller Medium and follow product preparation instructions.

<u>b. M9 Minimal Media</u>
1) Prepare Stock Solutions (sterile, solvent: DI H_2O)
5x M9 Salts (Autoclave)
0.1M $CaCl_2$ (Autoclave)
0.1M $MgSO_4$ (Autoclave)
2.5% (w/v) Casamino acid (Autoclave)
40% (w/v) D-glucose (Sterile filter)
0.1M thiamine hydrochloride (Sterile filter)
DI H_2O (Autoclave)

2) To make 1L of 5x M9 salts,
Mix:
15 g/L KH_2PO_4
2.5 g/L NaCl
64.0 g/L Na_2HPO_4-$7H_20$
5 g/L NH_4Cl
800mL DI H_2O

> Adjust to 1L DI H_2O and autoclave

3) To make 25mL of 2x M9 minimal media,
Mix:
10mL of 5x M9 Salts
50µL of 0.1M $CaCl_2$
1mL of 0.1M $MgSO_4$

4mL of 2.5% (w/v) Casamino acid
1mL of 40% (w/v) D-glucose
0.5mL of 0.1M thiamine hydrochloride
8.5mL of autoclaved DI H_2O

4) To make the final 10mL of 1x M9 minimal media with object entry,
Mix:
2x M9 minimal media (5mL)
Concentrated entry solution (x mL) (if applicable)
100x Antibiotics mix (0.1mL)
Autoclaved DI H_2O ((5–x) mL)

c. Phosphate-buffered Saline (PBS Buffer)
To make 1L of PBS buffer,
Mix:
8 g of NaCl
0.2 g KCl
1.44 g Na_2HPO_4
0.245 g KH_2PO_4
800mL DI H_2O

> Adjust to 1L DI H_2O and autoclave.
*Adjust solution to desired pH 7.4 with HCl or NaOH.

4. Metrics used to determine completion of the ethical hacking exercise. Successful completion of the penetration test should satisfy the following criteria: (i) entry of the putatively correct authentication code must result in the expression of GFP in at least 80% of the chassis cell population on average, measured using a flow cytometer with statistical significance ($n \geq 3$). (ii) The red team must provide the exact authentication code that resulted in the expression of the asset. (iii) The *11P3* exercise must be completed within 30 days – including validation testing.

Additional Instructions

1. When you (the red team) receive the strains, prepare a cell stock immediately, and store the samples in the freezer at -80ºC.
2. Prepare a working sample from a cell stock as follows: streak cells from said stock onto an agar plate with the provided antibiotics. Agar plates > 1 week old should be re-streaked on an agar plate the with freshly prepared antibiotics. Pre-culture the colony into LB media.

Decryption testing protocol:
Refer to the instructions below for the assay protocol to test authentication codes. All steps should be conducted under sterile conditions. A visual representation is also provided (see **fig. S24**).

1. Take the agar stabbed colonies and pre-culture them into LB broth media supplemented with antibiotics (for a minimum time of 1 hour, at 37ºC, while shaking at 750 rpm) and then streak the cells on an agar plate supplemented with antibiotics. Incubate the plate overnight at 37ºC.
2. Pick a colony from the agar plate and start a pre-culture in 200 uL LB Broth media supplemented with antibiotics, in a 96-well plate. Repeat this for desired number of biological replicates Seal the plate with a Breath Easier membrane. Culture cells for 8 hours at 37°C, while shaking at 300 RPM.

3. Entry 1.1: After 8 hours, the cells should be diluted at a 1:200 ratio into M9 minimal media with the first entry (1uL of cell culture + 199uL of M9 minimal media with the selected entry). This should be in a 96-well plate and sealed with a Breath Easier membrane. Grow the cells for 12 hours at 37°C, while shaking at 300 rpm.
4. Entry 1.2: After 12 hours of growth, dilute the cell cultures at a 1:200 ratio into fresh M9 minimal media with the same entry used in step 3 (1uL of cell culture + 199uL of M9 minimal media with the selected entry). Seal the 96-well plate with a Breath Easier membrane and grow the cells for an additional 12 hours. NOTE: Cells should be diluted in fresh minimal media at a 1:200 ratio every 12 hours for ideal cellular growth – each entry exposure should be 12 hours x 2 = 24 hours (*i.e.*, expose twice with same object entry) for best performance.
5. Entry 2.1: For the second entry, dilute the grown cells from step 4 at a 1:200 ratio into fresh M9 minimal media with the second entry (1uL of cell culture + 199uL of M9 minimal media with the selected entry). Seal the 96-well plate with a Breath Easier membrane and grow the cells for an additional 12 hours. NOTE: Authentication codes do NOT use the same object entry more than once.
6. Entry 2.2: After 12 hours of growth, dilute the cell cultures at a 1:200 ratio into fresh M9 minimal media with the same entry used in step 5 (1uL of cell culture + 199uL of M9 minimal media with the selected entry). Seal the 96-well plate with a Breath Easier membrane and grow the cells for an additional 12 hours.
7. Entry 3.1: For the third object entry, dilute the grown cells from step 6 at a 1:200 ratio into fresh M9 minimal media with the third entry (1uL of cell culture + 199uL of M9 minimal media with selected entry). Seal the 96-well plate with a Breath Easier membrane and grow the cells for an additional 12 hours. NOTE: Authentication codes do NOT use the same object entry more than once.
8. Entry 3.2: After 12 hours of growth, dilute the cell cultures at a 1:200 ratio into fresh M9 minimal media with the same entry used in step 7 (1uL of cell culture + 199uL of M9 minimal media with said entry). Seal the 96-well plate with a Breath Easier membrane and grow the cells for an additional 12 hours.
9. After the entry steps are completed, dilute the cells from step 8 into fresh M9 minimal media without any entries, at a 1:200 ratio (1uL of cell culture + 199uL of M9 minimal media without entries). Seal the 96-well plate with a Breath Easier membrane and grow the cells for an additional 12 hours.
10. After 12 hours, prepare the cells for FACS (see FACS Protocol).

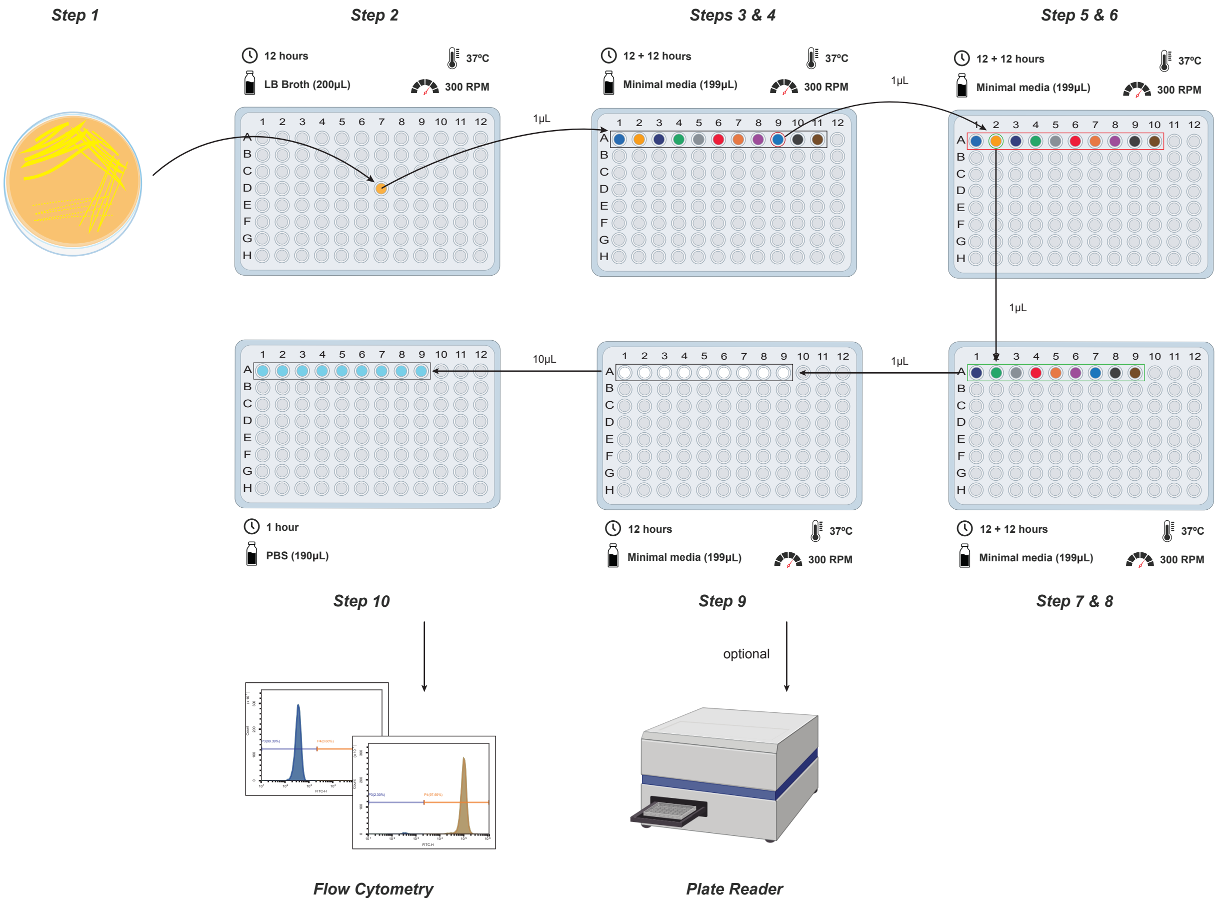

Fig. S24 | Illustration of the assay for *11P3* decryption testing.

FACS protocol:

FACS is required to quantify the population of cells that are expressing GFP. The instructions for preparing your cells for FACS are as follows (also see **fig. S25**):

1. After the 12-hour outgrowth in M9 minimal media without entries, dilute the cells at a 1:40 ratio into PBS with 2 mg/ml kanamycin in a 96-well plate (5uL of cell culture + 195uL of PBS) under sterile conditions. Next, incubate the plate for at least 1 hour at room temperature.
2. Set the flow rate for the flow cytometer between 10-30 ul/min. Next, the cells should be monitored through the FITC channel for GFP expression. Events should be gated by forward scatter area versus side scatter area to eliminate debris, and then gated by side scatter height versus side scatter area to discriminate doublets.
3. More than 50,000 events must be collected for the final analysis.

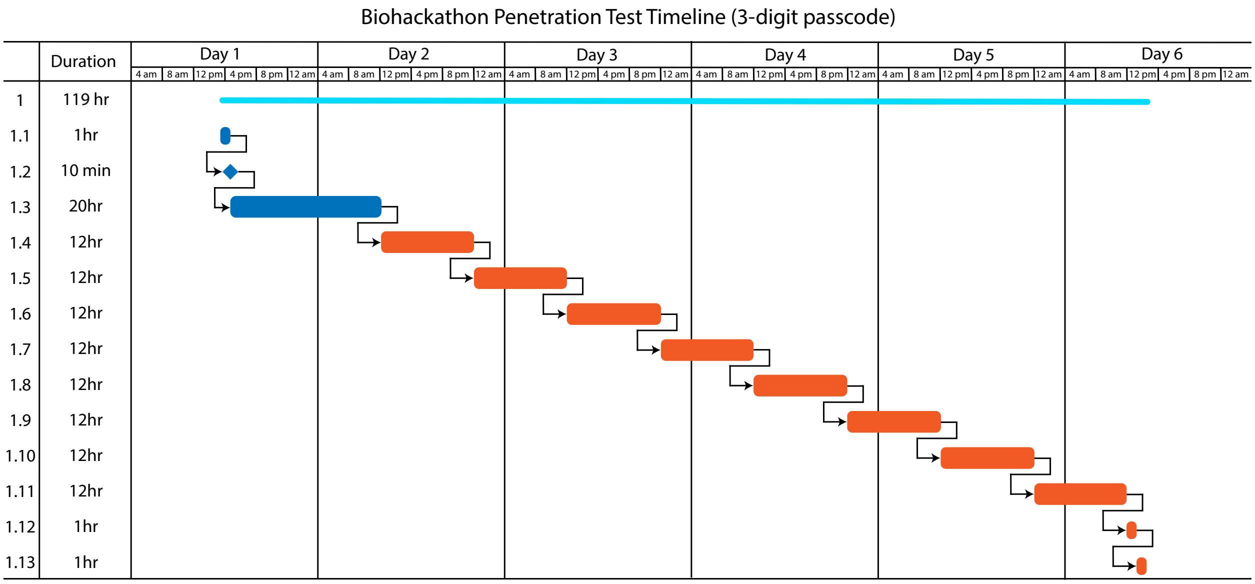

Legend

1	Biohackathon Penetration Test Timeline (Total)
1.1	Pre-culture agar-stabbed colony in LB broth
1.2	Streak pre-culture on agar plate
1.3	Incubate the plate overnight
1.4	Pre-culture individual colonies
1.5	Passage cells to minimal media with first digit inducers (ENTRY 1.1)
1.6	Passage cells to minimal media with first digit inducers (ENTRY 1.2)
1.7	Passage cells to minimal media with second digit inducers (ENTRY 2.1)
1.8	Passage cells to minimal media with second digit inducers (ENTRY 2.2)
1.9	Passage cells to minimal media with third digit inducers (ENTRY 3.1)
1.10	Passage cells to minimal media with third digit inducers (ENTRY 3.2)
1.11	Passage cells to minimal media with no inducers (Outgrowth)
1.12	Dilute cells into PBS for flow cytometry
1.13	Check fluorescence expression of cells with a flow cytometer

Fig. S24 | Instructions and illustration for preparing the *11P3* security system

Biohackathon Protocol: ***11P3*** **Security System (iteration 2)**

The protocol for iteration 2 remained the same as the protocol from iteration 1, except for the way the biological asset (testing strain) was prepared for the ethical hacking exercise. To ensure a pristine sample of the encrypted biological asset (*i.e.* no unpremeditated inversions and population heterogeneity), the sample was verified genotypically through the following steps:

1. Co-transform recombinase-transcription factor pairs plasmid and relevant encrypted biological asset plasmid and plate on LB agar with corresponding antibiotics (chloramphenicol and kanamycin). Incubate the plate overnight at 37ºC.
2. Pick 14 colonies and conduct 50 uL colony PCRs using primers that bind to the kanamycin resistance gene (which is expressed only in the encrypted biological asset plasmid).
3. Pre-culture the colonies in LB broth media supplemented with the antibiotics (at 37ºC, while shaking at 750 rpm) until sequencing is verified.
4. Gel extract the 14 colony PCRs and verify their sequences by nanopore linear sequencing.
5. Based on which sample showcases the correct sequence of the encrypted biological asset plasmid, use its pre-culture and streak the cells on an agar plate supplemented with the antibiotics. Incubate the plate overnight at 37ºC.
6. Begin the **Decryption testing protocol** from step 2.

Table S1| Source data for main text figures. This table contains the source data for main text figures: **Fig. 2**, **Fig. 3**, **Fig. 4**, **Fig. 5**, **Fig. 6**, **Fig. 7**, and **Fig. 8**

Table S2| Source data for supplementary information figures. This table contains the source data for supplementary information figures: fig. S5, fig. S6, fig. S7, fig. S8, fig. S9, fig. S10, fig. S11, fig. S12, fig. S13, fig. S14, and fig. S16
